# Genome-wide detection of CRISPR editing in vivo using GUIDE-tag

**DOI:** 10.1038/s41467-022-28135-9

**Published:** 2022-01-21

**Authors:** Shun-Qing Liang, Pengpeng Liu, Jordan L. Smith, Esther Mintzer, Stacy Maitland, Xiaolong Dong, Qiyuan Yang, Jonathan Lee, Cole M. Haynes, Lihua Julie Zhu, Jonathan K. Watts, Erik J. Sontheimer, Scot A. Wolfe, Wen Xue

**Affiliations:** 1 RNA Therapeutics Institute, University of Massachusetts Medical School, Worcester, MA USA; 2 Department of Molecular, Cell and Cancer Biology, University of Massachusetts Medical School, Worcester, MA USA; 3 Department of Molecular Medicine, University of Massachusetts Medical School, Worcester, MA USA; 4 Program in Bioinformatics and Integrative Biology, University of Massachusetts Medical School, Worcester, MA USA; 5 Li Weibo Institute for Rare Diseases Research, University of Massachusetts Medical School, Worcester, MA USA

**Keywords:** Biologics, CRISPR-Cas9 genome editing, Genetic engineering

## Abstract

Analysis of off-target editing is an important aspect of the development of safe nuclease-based genome editing therapeutics. in vivo assessment of nuclease off-target activity has primarily been indirect (based on discovery in vitro, in cells or via computational prediction) or through ChIP-based detection of double-strand break (DSB) DNA repair factors, which can be cumbersome. Herein we describe GUIDE-tag, which enables one-step, off-target genome editing analysis in mouse liver and lung. The GUIDE-tag system utilizes tethering between the Cas9 nuclease and the DNA donor to increase the capture rate of nuclease-mediated DSBs and UMI incorporation via Tn5 tagmentation to avoid PCR bias. These components can be delivered as SpyCas9-mSA ribonucleoprotein complexes and biotin-dsDNA donor for in vivo editing analysis. GUIDE-tag enables detection of off-target sites where editing rates are ≥ 0.2%. UDiTaS analysis utilizing the same tagmented genomic DNA detects low frequency translocation events with off-target sites and large deletions in vivo. The SpyCas9-mSA and biotin-dsDNA system provides a method to capture DSB loci in vivo in a variety of tissues with a workflow that is amenable to analysis of gross genomic alterations that are associated with genome editing.

## Abstract (web summary)

In vivo assessment of nuclease off-target activity has primarily been indirect or through ChIP-based detection of double-strand break DNA repair factors, which can be cumbersome. Here, the authors show that GUIDE-tag, enables one-step off-target genome editing analysis in mouse liver and lung.

## Introduction

Understanding the safety profile of each genome editing system is a critical aspect in the development of effective therapeutics. One critical characteristic of any nuclease system is its specificity. Unwanted off-target editing can generate local mutations^1,2^, larger deletions^3^, or genomic rearrangements^4,5^ between two different double strand breaks (DSBs). Knowledge of any off-target sites for a nuclease provides an avenue to mitigate collateral damage to the genome through the use of engineered Cas9 variants^6–13^, sgRNA modifications^14–16^, or alternate approaches^17^. Defining off-target sites within the genome currently relies on both computational prediction^18,19^ and unbiased assessments of nuclease activity^20^. Robust methods for off-target assessment are available both in vitro^21–25^ and in cell culture systems^4–6,26,27^. However, in vivo assessments of nuclease activity have typically been indirect employing in vitro or in cells to identify candidate off-target sites and then performing a survey of these sites by amplicon deep sequencing from nuclease-treated tissue (e.g., Verification of In Vivo Off-targets: VIVO^28^). Direct detection of off-target editing in vivo has been achieved by ChIP-based detection of DSB DNA repair factors (Discovery of in situ Cas off-targets and verification by sequencing: DISCOVER-seq^26^), but this method is somewhat cumbersome and captures DSBs in a temporally restricted window. The development of a sensitive, in vivo DSB detection method, would have utility for the evaluation of a variety of nucleases and delivery systems that are of interest for therapeutic application.

GUIDE-seq^4^ has been a mainstay for genome-wide analysis of off-target sites in primary cells^21,29^ and transformed cell lines due to its high validation rate relative to most in vitro methods. GUIDE-seq relies on the co-delivery of a linear double-strand DNA with the nuclease of interest to tag DSBs that are generated throughout the genome. Following genomic DNA fragmentation and unique molecular identifier (UMI)-containing adaptor ligation, PCR-based amplification using a tag-specific primer allows identification and counting of genomic regions neighboring nuclease cleavage sites thereby providing an assessment of nuclease specificity. DSB DNA tag integration is thought to be mediated by the nonhomologous end joining (NHEJ) pathway, which can insert linear duplex DNAs or DNA excised from plasmid at the site of a nuclease-induced DSB with modest efficiency in cells or in vivo^30–33^.

Tethering the Cas9-sgRNA complex to a donor DNA has proven to be an effective method for template-directed genome modification by increasing the local concentration of the donor at the site of a DSB. Several methods for recruiting the DNA template to the Cas9-sgRNA complex to enhance homology-directed repair (HDR) have been reported in cells or mouse zygotes: (1) high-affinity biotin-streptavidin interactions^34^, (2) direct or indirect covalent tethering, or (3) chemical modification^35–39^. The majority of these studies focus on employing tethered donors to increase the rate of HDR, but the same rationale should apply to the use of Cas9 tethered dsDNA donors to increase the efficiency of insertion via NHEJ at DSBs in adult animals.

In this study, we have adapted SpyCas9-mSA (monomeric streptavidin)^34^ and biotinylated duplex DNAs to increase the efficiency of targeted DNA insertion at DSB in vivo. In addition, we have utilized Uni-Directional Targeted Sequencing (UDiTaS^40^) library construction methods to incorporate UMI-containing adaptors to increase the efficiency of library construction thereby reducing the required input DNA. Using this “GUIDE-tag” system, we have achieved efficient duplex DNA capture at nuclease-induced DSBs at off-target sites within the mouse liver and lung via two different delivery modalities with the capture of sites where the editing rate is ≥0.2%. Complementary UDiTaS analysis allows the detection of additional genomic modifications (large deletions or translocations) in vivo that are the consequence of programming SpyCas9 with promiscuous guides. Consequently, GUIDE-tag provides a direct and sensitive strategy for the identification of off-target editing sites in vivo.

## Results

### SpyCas9-mSA with biotin-dsDNA donor increases the efficiency of DNA insertion in cell culture systems

**Fig. 1:**
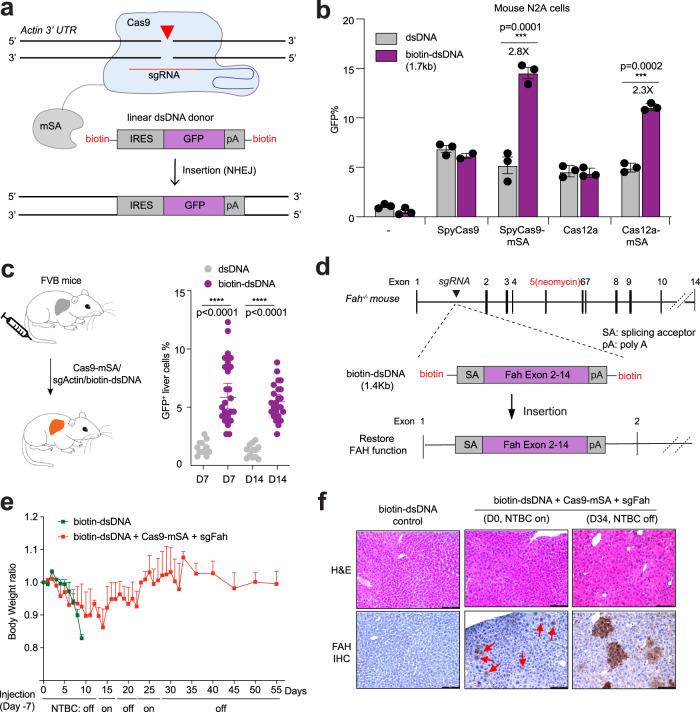
Tethering Cas9-mSA with biotin-dsDNA donor increases DNA cassette insertion rate in mouse liver. **a** Schematic of the Cas9-mSA fusion protein with an sgRNA directing a DSB to the *Actb* (Actin) locus. The “IRES-GFP-pA” biotinylated linear dsDNA donor is tethered to Cas9 via the mSA–biotin interaction to facilitate its insertion via non-homologous end joining (NHEJ). GFP expression reports integration of the IRES-GFP cassette in the forward orientation. pA, polyA. **b** Mouse N2A cells were transfected with IRES-GFP donor (dsDNA or biotin-dsDNA) and various nuclease plasmids: SpyCas9/sgRNA, SpyCas9-mSA/sgRNA, enAsCas12a/crRNA, or enAsCas12a-mSA/crRNA. Flow cytometry analysis was performed 4 days after transfection. Biotin-dsDNA with SpyCas9-mSA or enAsCas12a-mSA increases GFP^+^ cells 2.8 fold or 2.3 fold compared to unmodified dsDNA, respectively. Results were obtained from three independent experiments and presented as mean ± SEM. ***P < 0.001 by two-way ANOVA with Tukey’s multiple comparisons test. “-” denotes donor only controls. **c** SpyCas9-mSA/sg*Actb* expression vector plus IRES-GFP donor (dsDNA or biotin-dsDNA) were delivered to FVB mouse liver by hydrodynamic tail-vein injection (left). At the indicated timepoint the fraction of GFP^+^ cells in the liver was quantified by immunohistochemistry (IHC) (right). Each dot represents the quantification of a 20× IHC image (4 mice per group). Results were obtained from six independent experiments and presented as mean ± SD. ***P < 0.001 by one-way ANOVA with Tukey’s multiple comparisons test. **d** Schematic overview of gene repair in *Fah*-deficient mice. Fah^−/−^ mice harbor a neomycin cassette in exon 5, which abolishes FAH function. Insertion of a repair cassette (*Fah* exon 2 to 14 with splicing acceptor, SA) into intron 1 will restore FAH function. **e** Fah^−/−^ mice were injected with either biotin-dsDNA alone (control), or biotin-dsDNA plus Cas9-mSA+sgFah expression plasmid (*n* = 6) seven days prior to intial NTBC withdrawal. Two cycles of NTBC withdrawal (D0, D17, and D27) and reintroduction (D13 and D22) were performed to allow expansion of FAH^+^ hepatocytes. Body weight was normalized to pre-injection weight. Error bars are SD. **f** Hematoxylin and eosin (H&E), and FAH IHC analysis (*n* = 3 mice for each group). Arrows denote FAH^+^ hepatocytes. The scale bar is 100 µm. Source data are provided as a Source Data file.

We first tested whether biotin-dsDNA donors enable targeted NHEJ-mediated insertion in an easy-to-transfect mouse neuroblastoma cell line, N2A. We chose to insert an IRES-GFP fragment (1.7 Kb) into the 3′ UTR of *Actb (beta-actin)* at a previously described SpyCas9 target site, such that the insertion in the forward orientation leads to co-expression of GFP from the *Actb* promoter^41^ (Fig. 1a, Supplementary Fig. 1a, b, and Supplementary Table 1). Using one-step PCR with either unmodified primers or 5′ biotinylated primers from a plasmid donor template^32,41^, we generated unmodified linear dsDNA or biotin-dsDNA donor cassette, respectively (Supplementary Table 2). We transfected either dsDNA or biotin-dsDNA with or without expression plasmids for SpyCas9-mSA^34^ (or SpyCas9) and sgRNA into N2A cells. After 4 days we determined the cassette insertion efficiency by quantifying the percentage of GFP^+^ cells by flow cytometry (Fig. 1b). We observed a low frequency of GFP^+^ cells when delivering dsDNA (1.1 ± 0.1%) or biotin-dsDNA donor (0.5 ± 0.2%) alone, likely due to random insertion. In the presence of SpyCas9, we observed a higher fraction of GFP^+^ cells for both dsDNA (6.9 ± 0.3%) and biotin-dsDNA (6.2 ± 0.2%), demonstrating that biotinylated dsDNA generated by PCR is competent for homology-independent insertion in cells. Notably, biotin-dsDNA produces a 2.8-fold increase in insertion efficiency (14.5 ± 0.6%) relative to unmodified dsDNA in the presence of SpyCas9-mSA (5.2 ± 0.8%) (*P* < 0.001). To confirm targeted insertion, we amplified the 5′ junction site and 3′ junction site by PCR (Supplementary Fig. 1c). A similar enhancement in donor DNA insertion by the SpyCas9-mSA/biotin-dsDNA system was observed in human cells. We compared the efficiency of insertion of an IRES-GFP fragment (1.7Kb) into the 3′ UTR of *GAPDH*. We observed a higher fraction of GFP^+^ cells when SpyCas9-mSA (9.9 ± 0.6%) was co-introduced with biotin-dsDNA than dsDNA (Supplementary Fig. 1d). These data indicate that tethering SpyCas9-mSA with biotin-dsDNA donor promotes increased homology-independent cassette insertion at the DSB in cells in culture.

To explore the translatability of the biotin-dsDNA–mSA facilitated insertion to other nuclease systems, we generated vectors expressing either enhanced *Acidaminococcus sp*.Cas12a variant^42^ fused with mSA (enAsCas12a-mSA) or *Staphylococcus aureus* Cas9^43^ fused with mSA (SauCas9-mSA). To quantify the insertion efficiency, we again targeted an IRES-GFP fragment (1.7 kb) into the 3′ UTR of *Actb* of N2A cells. We transfected either dsDNA or biotin-dsDNA with or without expression plasmids for SauCas9-mSA or enAsCas12a-mSA and the corresponding sgRNA or crRNA into N2A cells. Similar to Spy9Cas9, we observed a higher fraction of GFP^+^ cells for both enAsCas12a-mSA (11.1 ± 0.2%) and SauCas9-mSA (8.9 ± 0.1%) with biotin-dsDNA than dsDNA (Fig. 1b and Supplementary Fig. 1e). These data indicate that biotin-mSA tethering between the donor and a co-delivered nuclease can provide a general method to promote increased cassette insertion in cells.

### biotin-dsDNA donor mediates insertion of reporter gene and rescue cassette in mouse liver

Next, we tested the efficiency of biotinylated dsDNA cassette insertion mediated by SpyCas9-mSA in adult mouse liver. We delivered the 1.7 kb IRES-GFP donor either as linear dsDNA or biotin-dsDNA with SpyCas9-mSA/sg*Actb* expression plasmid to wildtype FVB mice (n = 3 for each group) via hydrodynamic tail vein injection (HTVI), a method for in vivo gene delivery to hepatocytes^44^ (Fig. 1c). In mice treated with SpyCas9-mSA/sg*Actb* and dsDNA (7-days post injection), only 1.5 ± 0.7% of liver cells expressed GFP by immunohistochemistry staining (IHC) at day 7 (Fig. 1c), suggesting that unmodified dsDNA is inefficiently inserted via plasmid-expressed SpyCas9 in hepatocytes using HTVI delivery. By contrast, in mice treated with SpyCas9-mSA/sg*Actb* and biotin-dsDNA, 6.4%±2.7% (p < 0.001) of liver cells expressed GFP at day 7 and the GFP signal was comparable at 14 days (5.5 ± 1.5%, Fig. 1c), suggesting that the GFP insertion is stable. To test whether there are detectable GFP insertions in other organs when delivering editing components by HTVI. We performed IHC staining in the following tissue: heart, kidney, lung, brain, colon, muscle, and spleen. No GFP expression was observed in any tissue other than liver (Supplementary Fig. 1f). Thus, utilization of a biotin dsDNA donor in the context of SpyCas9-mSA increases the efficiency of DNA insertion in the liver relative to an unmodified dsDNA donor.

To directly assess the efficiency of targeted DNA insertion, we utilized a mouse model of hereditary tyrosinemia type I (HT-I)^45^. In this liver disease, loss of fumarylacetoacetate hydrolase (Fah) leads to accumulation of toxic metabolites in hepatocytes, and severe liver damage. Current treatments include consuming a tyrosine-restricted diet and taking an inhibitor of the tyrosine catabolic pathway called 2-(2-nitro-4-trifluoromethylbenzoyl)-1,3-cyclohexanedione (NTBC)^46^. More than 95 different pathologic mutations in the *FAH* gene (which contains 14 exons, spanning 35 kb of DNA) have been identified^47,48^. We and others have shown that CRISPR-mediated HDR can correct a splicing site mutation in the Fah mutant mice^49,50^. However, each HDR donor/sgRNA can only correct mutations in a narrow genomic region. Alternately a *Fah* repair cassette has been introduced via nuclease-mediated Microhomology-mediated end joining (MMEJ) in intron 4 to correct downstream disruptions to *Fah* with modest efficiency^51^. We examined whether SpyCas9-mSA can efficiently insert a biotinylated wildtype *Fah* exon 2–14 cDNA cassette into intron 1, which should rescue any *Fah* mutations downstream of the intron 1 insertion site.

We first screened four sgRNAs targeting intron 1 of the Fah gene in N2A cells. By Tracking of indels by decomposition (TIDE) analysis^52^, sgFah1.3 produced the highest editing efficiency (41 ± 0.3%), and so was selected for in vivo experiments (Supplementary Fig. 2a and Supplementary Table 1). We generated a *Fah* exon 2–14 cDNA donor flanked by a splice acceptor and polyA signal (1.4 kb in length) using PCR with biotinylated primers (Fig. 1d). Donor insertion in the forward orientation in intron 1 should produce a functional FAH protein by capturing transcription from the endogenous *Fah* promoter (Fig. 1d). To test the efficacy of the repair cassette, we used a Fah^−/−^ mouse model that harbors a neomycin cassette insertion in exon 5, which causes a loss of FAH protein^45^. Adult Fah^−/−^ mice on NTBC water were treated with biotin-dsDNA donor alone (*n* = 6) or with an sgFah and SpyCas9-mSA expression plasmid (n = 6) via HTVI. NTBC water was removed 7 days post injection (defined as NTBC on, D0) to assess the functional correction of *Fah*.

We performed two cycles of NTBC reintroduction and withdrawal following gene repair to allow the proliferation of Fah^+^ hepatocytes in treated mice^49^. As expected, Fah^−/−^ mice treated with biotin-dsDNA donor alone rapidly lost 20% of body weight by 17 days post-NTBC withdrawal and were sacrificed. Fah^−/−^ mice treated with biotin-dsDNA + sgFah + SpyCas9-mSA displayed a more moderate loss of body weight at day 17 (14%). By Day 34 off NTBC, these mice regained their initial body weight (Fig. 1e). In Day 0 livers prior to NTBC withdrawal, IHC analysis revealed 3.8 ± 0.4% Fah^+^ hepatocytes in mice treated with biotin-dsDNA + sgFah + Cas9-mSA but not in biotin-dsDNA alone controls (Fig. 1f, left and center panels). At day 34, we observed clusters of Fah^+^ hepatocytes, consistent with the proliferation of Fah^+^ hepatocytes underlying the restoration of body weight (Fig. 1f, right panel). We also detected the insertion of the repair cassette within the *Fah* locus by PCR (Supplementary Fig. 2b) and the inclusion of exon 5 within the Fah transcript by RT-PCR (Supplementary Fig. 2c). Collectively, these results demonstrate the fidelity of SpyCas9-mSA mediated insertion in mouse liver of a biotinylated repair cassette at a target locus via rescue of the lethal phenotype of *Fah*^−/−^ mice.

### One step quantification of on-target insertion and genome-wide off-target sites in vivo

**Fig. 2:**
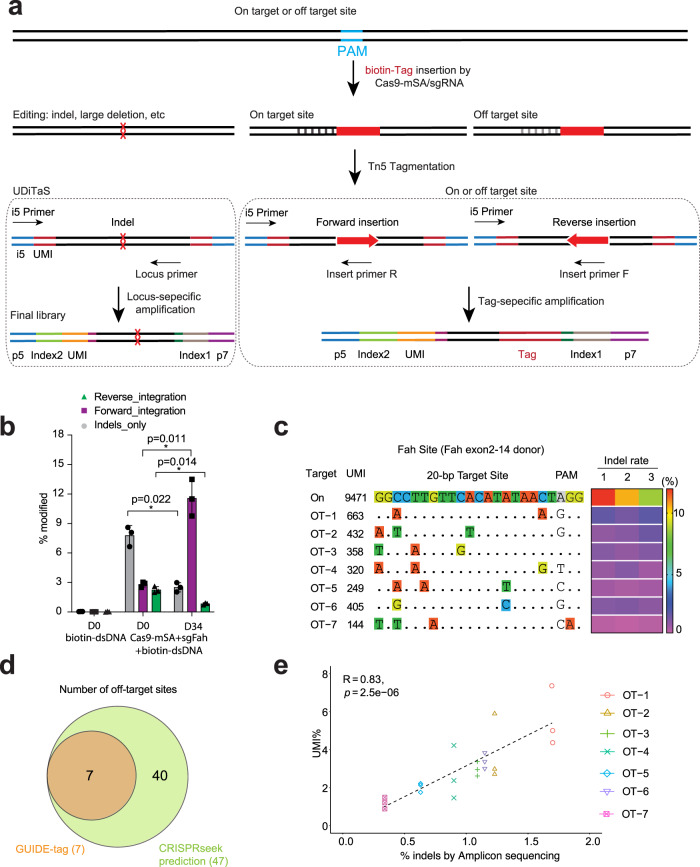
GUIDE-tag enables genome-wide off-target analysis in vivo. **a** Schematic of GUIDE-tag editing outcomes and strategies to create UDiTaS or GUIDE-tag Illumina sequencing libraries, where insertion is the DNA tag. Tn5 enzyme tagments genomic DNA and adds UMI, pooling barcode, and i5 primer sites. Desired genomic regions are amplified with i5 Primer and a viewpoint specific primer: for UDiTaS a locus-specific genomic forward or reverse (Locus_F or R) and for GUIDE-tag a tag-specific forward or reverse (Insert_F and R). A second round of PCR adds the i7 adaptor sequence. **b** Quantification forward/reverse donor insertion and indels (Locus_F) at the *Fah* target site in biotin-dsDNA plus Cas9-mSA+sgFah treated animals by UDiTaS. Genomic DNA was collected from NTBC on D0 and D34 mice in Fig. 1. Values are mean ± SD (*n* = 3 mice each group). * *P* < 0.05 by unpaired, two-tailed Student’s t-test. **c** In vivo off-target (OT) sites identified by GUIDE-tag in biotin-dsDNA plus Cas9-mSA+sgFah treated animals. Mismatches relative to the target site (On) are shown with colored boxes. UMI numbers for each site are shown (average of three mice). 1,2,3 are three different mice with indel rates indicated as a heatmap. **d** Venn diagram of sgFah off-target sites identified by GUIDE-tag or by CRISPRseek prediction. **e** Scatter plot shows correlation between unique UMI% and indel rates at the 7 sgFah off-target sites (average of 3 mice). Dashed lines represent the linear regression fit (Pearson correlation calculated). The *p*-value for Pearson’s correlation coefficient was determined by the two-tailed t-distribution table. Dots on *Y-*axis represent UMIs for each OT for each of 3 mice. Source data are provided as a Source Data file.

The efficient tagging of Cas9-directed DSBs by SpyCas9-mSA with biotinylated donor DNA presented the opportunity to explore the potential for this system to identify off-target editing in vivo. GUIDE-seq^4^ or variants thereof^6,53^ utilize linear duplex DNA co-delivered with a nuclease to tag DSBs within the genome in cells. We endeavored to assess if our SpyCas9-mSA biotin-DNA tagging strategy would facilitate the identification of off-target editing sites in vivo. We adapted the UDiTaS method^40^ to generate “GUIDE-tag” libraries where Tn5 transposase loaded with the Illumina forward adapter (i5) containing a sample barcode and a unique molecule identifier (UMI) is used to fragment the gDNA. As in the original GUIDE-seq protocol, the incorporation of UMIs permits both quantitative sequence analysis (alleviating the PCR amplification bias) and detection of rare integration events. The utilization of Tn5 allows much more efficient incorporation of the UMI-containing adaptors relative to traditional end repair approaches after shearing of the DNA. Tagmentation was performed on genomic DNA from Fah^−/−^ mice treated with SpyCas9-mSA and the Fah exon2–14 donor. Four different sequencing libraries were prepared from each genomic DNA sample amplifying with a viewpoint specific primer in conjunction with an adaptor specific primer (Fig. 2a): two UDiTaS-based “locus-specific” viewpoints with primers flanking the target site to provide information on the editing outcomes at the target locus, and two GUIDE-seq-based “insert-specific” viewpoints with primers anchored within the donor DNA to identify repair cassette integration sites throughout the genome (Supplementary Fig. 3a,b and Supplementary Table 3). Together these four libraries allow the detection of on-target and off-target DNA insertions, as well as large-scale chromosomal aberrations at the target locus.

Based on the UDiTaS analysis, we observed Fah biotin-dsDNA integration rates at the target site of 2.8 ± 0.2% in the forward orientation and 2.3 ± 0.2% in the reverse orientation at the *Fah* target site at day 0 (*n* = 3 livers) (Fig. 2b and Supplementary Table 4), which is consistent with the expectation that NHEJ integration will insert donors in both orientations^54^. A similar distribution of integration orientations was observed by UDiTaS from the other viewpoint at the *Fah* locus (Supplementary Fig. 4a). The observed rate of forward integrations is consistent with the day 0 IHC data on Fah^+^ cells in the liver (~3.8%, Fig. 1f). As expected, the percentage of repair cassette insertions in the forward orientation increased significantly at D34 (11.6 ± 1.9%) due to the proliferation of Fah^+^ hepatocytes with functional cassettes (Fig. 2b). The percentage of cassette insertions in the reverse orientation (0.8 ± 0.1%) and indels (2.5 ± 1.5%) decreased at D34 relative to D0 (Fig. 2b). The majority of forward orientation insertions exhibit imprecise repair at both junction sites (D34), with the fraction of precise insertions of ~3% at each junction (Supplementary Fig. 4b, c).

Using the GUIDE-tag (donor centric) sequencing of libraries from *Fah*^−/−^ mice injected with Fah biotin-dsDNA + SpyCas9-mSA (NTBC off D0, *n* = 3 mice), we mapped donor integration sites throughout the genome and identified 7 top-ranking OT sites, all of which harbor 1 to 3 nt mismatches within the guide region (Fig. 2c and Supplementary Data 1). Using the CRISPRseek software package^55^—forty seven potential off-target sites were predicted with the Fah sgRNA with 3 or fewer mismatches (Supplementary Data 2). Seven of these sites overlapped with the GUIDE-tag identified off-target sites (Fig. 2d and Supplementary Fig. 5a). To verify nuclease activity at these seven potential off-target sites, we measured the indel rate by targeted amplicon deep sequencing (*n* = 3 livers). Indels were detected at all seven of the off-target sites ranging from 0.98% to 2.28% (Supplementary Data 1). Encouragingly, the rate of mutagenesis at each off-target site positively (*R* = 0.83) correlated with the observed rate of DNA tag insertion (Fig. 2e and Supplementary Fig. 5b). To examine the efficiency of GUIDE-tag employing short double-strand oligonucleotides as a donor typically used for GUIDE-seq, we co-delivered iGUIDE duplex oligos (46 bp)^53^ with or without biotin with the sgFah and SpyCas9-mSA expression plasmid by HTVI to B6 mice. GUIDE-tag analysis successfully recovered the previously identified seven off-target sites plus two additional potential off-target sites (Supplementary Fig. 5c, d) demonstrating that short biotinylated double-strand oligonucleotides can efficiently discover off-target sequences in vivo. Of the top 9 identified sgFah OT sites, 4 sites are intragenic falling within introns. None of these occur within known cancer genes (723 cancer genes in COSMIC v92) (Supplementary Fig. 5e). Whole exome sequencing of liver DNA from hepatocytes in *Fah* repaired livers (D34) showed that bio-dsDNA+sgFah+SpyCas9-mSA did not induce additional SNV compared to bio-dsDNA control (Supplementary Fig. 5f).

To examine the generality of the GUIDE-tag approach, we prepared libraries from FVB mice injected with biotin-IRES-GFP donor with either SpyCas9-mSA or SpyCas9-mSA*, an improved version of SpyCas9-mSA with additional nuclear-localization sequences (NLS) to increase its cellular activity (Supplementary Fig. 6a–c). SpyCas9-mSA* increased integration rates of IRES-GFP donors of different lengths (1.7–4.5 kb) in N2A cells (Supplementary Fig. 6d–f). SpyCas9-mSA* modestly increased the rate of DNA tag incorporation in vivo at the target locus (2.0% versus 2.4%) (Supplementary Data 3), which is consistent with the higher rates of GFP + N2A cells observed with this nuclease (Supplementary Fig. 6d–f). We identified 12 top-ranking sgActin off-target sites from the livers of FVB mice, all of which harbor 0 to 3 mismatches in the guide regions (Supplementary Fig. 7a, b and Supplementary Data 4). We were able to validate off-target editing at 10 of these sites by amplicon deep sequencing with indel percentages from 0.28 to 5.73% (Supplementary Data 4). As observed for the Fah sgRNA, with the *Actb* sgRNA there was a positive linear correlation (*R* = 0.67) between the tag integration rate and editing rate at the identified off-target sites (Supplementary Fig. 7c, d). Together, these data suggest that GUIDE-tag enables quantification of genome-wide off-target sites in vivo.

### Comparing GUIDE-tag with VIVO and DISCOVER-Seq

**Fig. 3:**
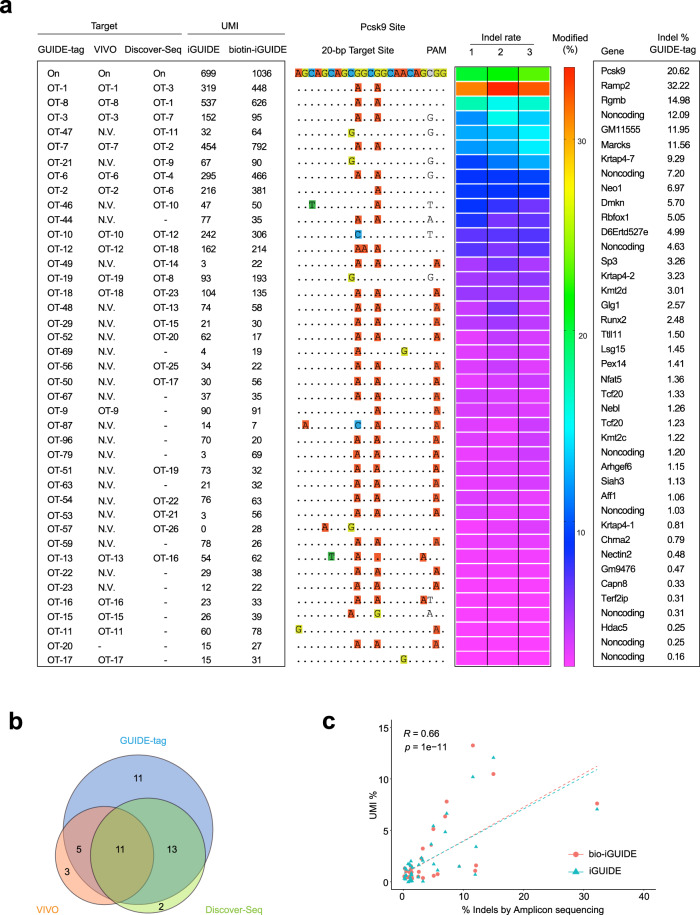
Comparisons of off-target sites discovered by GUIDE-tag, VIVO, and Discover-seq at *Pcsk9* locus. **a** Comparison of off-target site detection for a promiscuous sgRNA targeting *Pcsk9* (Pcsk9-gP or sgPcsk9) by GUIDE-tag, VIVO, and Discover-seq. For GUIDE-tag analysis genomic DNA was isolated from the liver of mice injected with plasmid expressing sgPcsk9-SpyCas9-mSA* plus iGUIDE or biotin-iGUIDE donors (*n* = 3). UMI numbers recovered for each site are shown (Average of three mice). Mismatches relative to the on-target site are shown with colored boxes. Indel frequencies determined by targeted amplicon sequencing from the liver of three mice are presented as a heat map (1,2,3 are three mice) and average indel% (right). Forty of 52 tested GUIDE-tag discovered off-target sites show statistically significant indels (*p* < 0.05) (ranked by average indel%). N.V, not verified, identified by CIRCLE-seq but not chosen for validation in the VIVO study. Dash, off-target site not identified by VIVO or DISCOVER-seq. **b** Venn diagram of the overlap of validated off-target cleavage sites discovered by GUIDE-tag (40 validated loci), VIVO (19 validated loci), and DISCOVER-seq (26 loci) for sgPcsk9. Note only a subset of potential off-target sites were tested in each study. **c** Scatter plots of average indel frequency (*x*-axis, targeted amplicon deep sequencing) and average UMI% (*y*-axis, *n* = 3 mice) for verified sgPcsk9 off-target sites identified by GUIDE-tag. Dashed lines represent the linear regression fit (Spearman’s correlation calculated). The *p*-value for Pearson’s correlation coefficient was determined by the two-tailed t-distribution table. Source data are provided as a Source Data file.

Next, we evaluated in vivo GUIDE-tag in mouse liver at a promiscuous target site in *Pcsk9* (Pcsk9-gP) that has been previously characterized by VIVO^28^ and DISCOVER-seq^26^. For this target site, we co-delivered iGUIDE duplex oligos (dsDNA or biotin-dsDNA) together with expression vectors for Cas9-mSA* and the sgRNA by HTVI. We then performed GUIDE-tag on mice sacrificed at day 7 post injection. GUIDE-tag libraries were prepared from genomic DNA and sequenced. Using a conservative (filter 1: UMIs ≥ 5 and total reads ≥ 50 in at least 4 mice of 6 iGUIDE treated mice) or a more relaxed (filter 2: UMIs ≥ 1 in at least 4 mice of 6 iGUIDE treated mice) threshold, we identified 70 (filter 1) or 89 (filter 2) off-target sites (Supplementary Fig. 8 and Supplementary Data 5). We observed a modest increase in the tag integration rate when employing biotinylated iGUIDE duplex (1.25-fold), but the enrichment is less pronounced than observed with the 1.7 kb GFP donor DNA at the *Actb* locus (Supplementary Fig. 9a, b). Putative off-target sites identified by GUIDE-tag include 16 of 19 validated off-target sites captured by VIVO and 24 of 26 validated off-targets captured by DISCOVER-Seq (Fig. 3a, b and Supplementary Data 5). We chose 52 sites (16 overlapping sites with VIVO, 24 overlapping sites with DISCOVER-Seq, 17 sites identified by CIRCLE-seq but not validated by VIVO^28^ and 6 sites identified only by GUIDE-tag) for amplicon deep sequencing to verify the presence of indels. Importantly, we were able to validate editing at 40 of the 52 tested sites based on statistical analysis. The indel rates range from 0.16 to 32.2% (Fig. 3a and Supplementary Data 5). Similar to observations at the *Actb* and *Fah* sites, PCR amplicon deep sequencing across forty off-target sites indicates that there is a positive linear correlation (*R* = 0.66) between the fraction of UMIs associated with each locus and the observed indel rate (Fig. 3c). Editing rates at the common off-target sites that were assessed appear to be similar across all three studies (Supplementary Fig. 9c, d). Furthermore, we repeated the in vivo GUIDE-tag experiment at the Pcsk9 target site (HTVI delivery) with the original GUIDE-seq donor (34 bp) and biotinylated-GUIDE-seq donor. The GUIDE-seq donor results in slightly more integrations than the iGUIDE donor based on UMI counts (Supplementary Fig. 9e and Supplementary Data 6). These data demonstrate that GUIDE-tag is compatible with both types of short donors in vivo. Notably, biotinylated donors are significantly better than non-biotin/end-protected donors based on the recovered UMI counts for top 50 OTs. Overall, these data demonstrate that GUIDE-tag provides a straightforward approach to detect genome editing at off-target sites in vivo.

### in vivo analysis of large-scale genomic alterations by UDiTaS

**Fig. 4:**
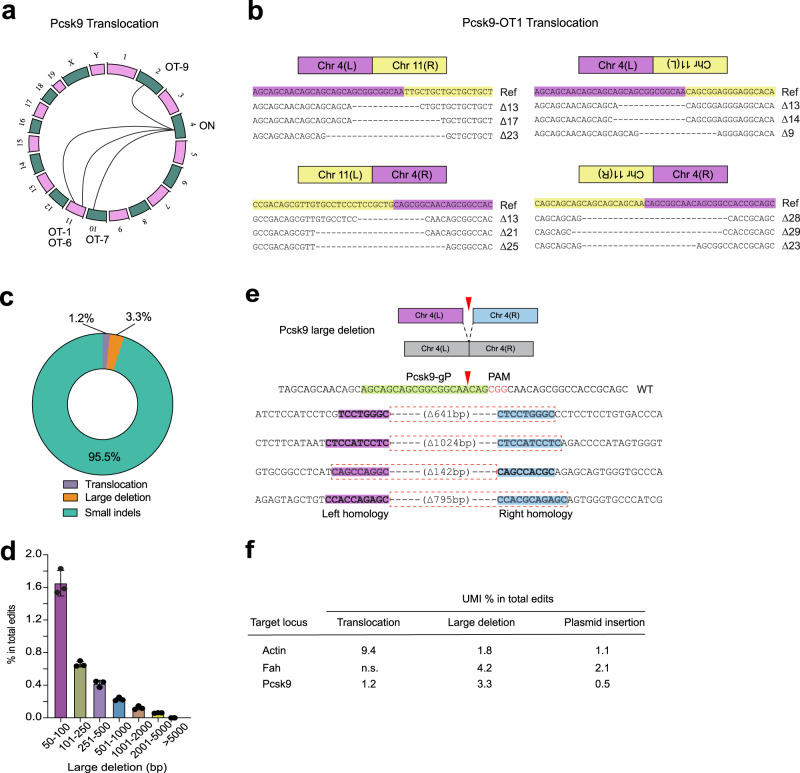
Analysis of in vivo translocations and large deletions by UDiTaS. **a** Circos plots of statistically significant translocations identified by UDiTaS at *Pcsk9* locus. Translocations are shown as arcs between the *Pcsk9* site and the chromosome position of each off-target site. **b** Examples of ligation junctions of translocations between the Pcsk9 site on chromosome 4 and the OT1 off-target site on chromosome 11. Reference (Ref) sequence is the predicted junction of the translocation for a precise ligation between the anticipated cleavage positions for the on-target and off-target site. The sequences below show representative small deletions for different translocation junctions that were observed. L and R denote left and right chromosome arms. **c** Fraction of edits that are small indels (<50 bp), large deletions (>50 bp) and translocations at Pcsk9 site by UDiTaS. Numbers are average of 3 mice. **d** Length distribution of large deletions (>50 bp) at Pcsk9 site. Values are mean ± SD (*n* = 3 mice). **e** Examples of large deletions at Pcsk9 site. Short homology arms present at the deletion junction are labeled magenta and blue, where the segment in the dotted red box is lost (including one of the homology arms) likely due to MMEJ-based repair. **f** Summary table of translocations and large deletion frequency detected by UDiTaS among all edited (non-wild type) sequences at three target sites. Vector insertion indicates the presence of plasmid sequence at the target site. n.s., not significant. Source data are provided as a Source Data file.

Utilization of the UDiTaS tagmentation approach with locus-specific primers for library construction^40^ affords the opportunity to assess the rate of large deletions^40^ or other types of genomic rearrangements^5,56^ in vivo, which remain relatively unexplored (Supplementary Fig. 10a). Genomic rearrangements between the target site and off-target site (Translocations or inversions/deletions when on the same chromosome) serve as an independent verification of off-target site activity^5^. UDiTaS analysis at the Pcsk9 locus revealed translocations between the target site and four off-target sites (Fig. 4a, b and Supplementary Data 7). Amplicon deep sequencing across off-target sites indicates that there is a positive correlation (*R* = 0.86) between the fraction of UMIs associated with translocation events and the observed indel rate at each off-target site (Supplementary Fig. 10b). Importantly, translocation between Pcsk9 locus and Ramp2 locus (OT-1) was validated by junction primers and sanger sequencing (Supplementary Fig. 10c, d). At the Pcsk9 locus more than 95% of the editing observed at the target site consists of small indels (<50 bp, Fig. 4c). We also observed 3.3% of the edited alleles contain large deletions (>50 bp) (Fig. 4c, d) and some large deletions harbor short homologous sequences at the deletion junction^57^ (Fig. 4e). Using UDiTaS, we were able to identify large deletions and translocations at the other two target sites (*Actb* & *Fah*) characterized in this study (Fig. 4f). UDiTaS analysis at the *Actb* locus reveals the presence of translocations between the target locus and three different off-target sites (Supplementary Fig. 10e). The high translocation frequency at the sg*Actb* target site is likely due to OT sites with the same sequence as the target site (Supplementary Fig. 7b). Together, these data suggest that UDiTaS enables the detection of nuclease-driven low frequency large-scale genomic alterations that occur in vivo.

### Genome-wide off-target analysis in mouse lung with SpyCas9-mSA RNP

Efficient genome editing in the lung would provide an opportunity to target the genetic causes of a number of disorders. McCray and colleagues have demonstrated the feasibility of editing the lung airway epithelia through intratracheal delivery of Cas9 or Cas12a RNPs with a shuttle peptide^58^. We examined the feasibility of RNP-based editing through intratracheal delivery when formulated with poly-L-glutamic acid (PGA), which can increase the efficiency of co-delivery of Cas9 RNPs and an HDR donor to primary cells via electroporation^59^. We delivered enAsCas12a protein plus a crRNA targeting the LoxP sequence formulated in PGA intratracheally in LSL-Tomato (Ai9) reporter mice^60^ to excise the stop cassette. Three days after 3 sequential deliveries of Cas12a RNP, IHC staining showed 10.0 ± 1.0% Tomato^+^ cells in the large airway surface of the lung (Supplementary Fig. 11).

**Fig. 5:**
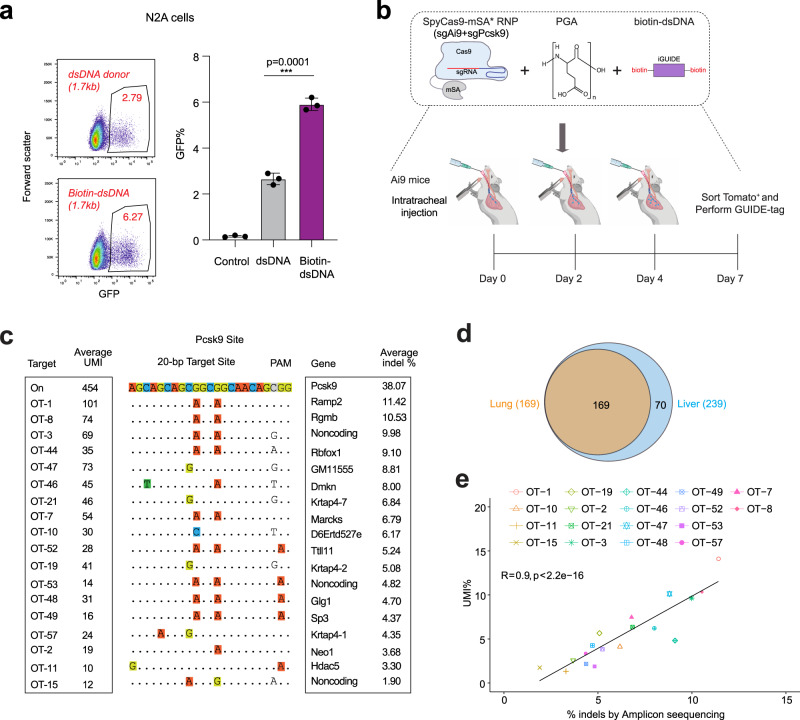
SpyCas9-mSA RNP delivery enables off-target editing discovery in the lung. **a** Delivery of RNP and donor DNA in vitro. Mouse N2A cells were transfected by Lipofectamine CRISPRmax with SpyCas9-mSA* sgActin (UTR) RNP and IRES-GFP donor (dsDNA or biotin-dsDNA). Flow cytometry analysis for GFP^+^ cells was performed 4 days after transfection. Results from three independent experiments are presented as mean ± SEM. ***P < 0.001 by one-way ANOVA with Tukey’s multiple comparisons test. **b** For GUIDE-tag analysis in lung, Ai9 mice were injected intratracheally with polymer-stabilized sgAi9-SpyCas9-mSA* RNP, sgPcsk9 SpyCas9-mSA* RNP plus biotin-iGUIDE donor (*n* = 2). GUIDE-tag libraries were prepared with gDNA isolated from sorted tdTomato^+^ lung cells. **c** List of validated OT sites in the lung. Average UMI numbers and average indel frequencies determined by targeted amplicon sequencing of sorted Tomato+ cells are shown (ranked by indel%, *n* = 2 mice). Mismatches relative to the on-target site are shown with colored boxes. **d** Venn diagram comparing potential OT sites discovered in the lung and liver by GUIDE-tag. **e** Scatter plots of average indel frequency and average UMI% for validated sgPcsk9 off-target sites in the lung. Dashed lines represent the linear regression fit (Spearman’s correlation calculated). The *p*-value for Pearson’s correlation coefficient was determined by the two-tailed t-distribution table. Source data are provided as a Source Data file.

To demonstrate the feasibility of targeted DNA insertion by SpyCas9 RNP within the lung, we purified SpyCas9-mSA* protein. Transfection of N2A cells with SpyCas9-mSA* RNP containing sgActin (UTR) and IRES-GFP donor resulted in increased insertion rate for a biotin-dsDNA donor compared to unmodified dsDNA donor (Fig. 5a). Consistent with the increased DNA insertion rate, native electrophoretic mobility shift assays (EMSA) demonstrate an association between biotinylated-donor DNA and SpyCas9-mSA* protein (Supplementary Fig. 12a). To demonstrate that tethering the donor template to Cas9 through mSA is an important element for the increased insertion efficiency, we co-transfected plasmids with separate expression cassettes for NLS-mSA and SpyCas9 or the fused system with biotin-IRES-GFP donor into N2A cells. The highest rate of GFP^+^ cells were observed when the SpyCas9-mSA fusion was used (Supplementary Fig. 12b). These data suggest that simple nuclear localization of the DNA donor is not the only important feature that is afforded by the Cas9-mSA system.

Having established the functionality of the SpyCas9-mSA* RNP for DNA insertion in vitro, we co-delivered SpyCas9-mSA*+sgPcsk9 RNP with bio-iGUIDE donor intratracheally in Ai9 reporter mice three times over a four day span. We also included SpyCas9-mSA*+ Ai9 sgRNA^61^ RNP in the delivery under the assumption that Ai9 edited Tomato positive cells would also have successful delivery of the Pcsk9 targeting complex, which would allow enrichment of the edited population (Fig. 5b and Supplementary Fig. 12c). We employed the GUIDE-tag protocol on genomic DNA from sorted Tomato positive cells to assess editing at the target site and throughout the genome. This analysis confirmed the incorporation of the iGUIDE donor at the Pcsk9 target site (Fig. 5c and Supplementary Figs. 13, 14). With gDNA from sorted cells, we identified 169 potential off-target sites in the lung (Fig. 5d and Supplementary Data 8). All of the potential off-target sites recovered in the lung overlap with off-target sites that were identified from editing in the liver (Fig. 5d). We chose 18 off-target sites for amplicon deep sequencing and validated editing at the tested sites (indel rates range from 1.9 to 11.4%) (Fig. 5c and Supplementary Data 8). Notably, there is a positive linear correlation (*R* = 0.9) between the fraction of UMIs observed at each of the off-target sites and the observed indel rate (Fig. 5e).

### Examining alternate forms of biotinylated donors for GUIDE-tag analysis

We observed a modest increase in the tag integration rate when employing synthetic biotinylated GUIDE-seq donor relative to the unbiotinylated donor in mice (Supplementary Fig. 9e). This is in contrast to the >3-fold improved integration rate observed for the PCR-generated biotinylated IRES-GFP donor relative to the unbiotinylated donor in mice (Fig. 1c). To analyze integration rates for different types of synthetic biotinylated donors (dsODN), we employed Hepa1–6 cells, where we tested integration rates of Guide-seq donors with different types and number of tethered biotins (Supplementary Fig. 15a). We first performed a dose response for editing with 3xNLS-SpCas9^62^ RNP with or with the mSA fusion targeting the PCSK9 locus to define subsaturating editing conditions (Supplementary Fig. 15b). Similarly, we titrated the concentration of an internally biotinylated Guide-seq donor to define subsaturation conditions to measure Cas9-mediated dsODN insertion rates for comparison of different donors (Supplementary Fig. 15c). Unlike our in vivo analysis, Guide-seq donors with two 5′ terminal Biotins (GS1-5′Bio + GS2-5′Bio) under sub-saturating editing conditions displayed lower insertion rates than 5′ phosphorated donors (GS1 + GS2) (Supplementary Fig. 15d–f). By contrast, Guide-seq donors with internal biotin (Biotin dT, IDT) displayed higher insertion rates with SpyCas9 RNP under subsaturating conditions (2pmol RNP and 5pmol dsODN) and further improvement when delivered with SpyCas9-mSA RNP (Supplementary Fig. 15d–f). These data suggest that Guide-seq donors with an internal Biotin may provide an improved donor for GUIDE-tag in vitro in some contexts, and they should be compatible with the RNP delivery method for in vivo off-target editing analysis.

## Discussion

Our DSB tagging methodology leverages the previously described SpyCas9-mSA system that can increase the efficacy of HDR in mouse embryos via tethering a biotinylated donor DNA^34^. We have extended this system to adult animals, where we employed the SpyCas9-mSA–biotin DNA tethering system to insert large linear dsDNA donors (>1 kb) containing a reporter gene or rescue cassette in the mouse liver. We demonstrated proof-of-concept for in vivo rescue of the disease phenotype of HT-I mice by inserting a donor dsDNA in the 1st intron of *Fah (*Fig. 1*)*. Although the correct forward insertion rate is low (3–6%) due to the modest efficiency of hepatocytes editing by HTVI, the insertion rate is comparable with our previous study^49^ using nanoparticle delivery of Cas9 mRNA and AAV delivery of HDR donor (~6% corrected hepatocytes) and is substantially higher than an MMEJ-based integration approach (~0.2%) also utilizing HTVI^51^. None of the 9 identified sgFah OT sites occur within known cancer genes or DNA repair genes (Supplementary Fig. 5e), and we did not observe any liver tumors or genetic evidence of genome instability (based on WES) after two months proliferation under selective pressure. The functional consequence of off-target editing events in vivo warrants further investigation as therapeutic translation of editing approaches are envisioned for specific disorders, but our chosen target sites were not optimized for the minimization of off-target events, and we are not employing high fidelity versions of SpyCas9 that can attenuate undesired editing events^13^. Future work can also evaluate the efficacy and safety of alternate Cas9-DNA tethering systems for NHEJ-mediated dsDNA insertion efficiency, as well as the delivery of dsDNA larger than 4.8 kb (AAV capacity^63^) in cells and mouse models, as a potential therapeutic approach for gene repair.

The increased insertion rate of large linear dsDNA donors at Cas9 DSBs in vivo by the SpyCas9-mSA tethering system spurred the development of the in vivo GUIDE-tag platform for identifying off-target sites. Current strategies for the in vivo assessment of nuclease activity have been indirect (e.g., VIVO^28^) or involve more cumbersome approaches for off-target identification (DISCOVER-seq^26^ or ITR-seq^64^). We demonstrated the feasibility of the identification of Cas9 off-target sites by GUIDE-tag when delivering components through HTVI. GUIDE-tag was able to detect genuine nuclease off-target sites with an indel frequency as low as 0.2%, which is approaching the typical limit of detection (0.1%) by NGS. In direct comparisons at the *Pcsk9* locus with VIVO and DISCOVER-Seq, GUIDE-tag successfully captured 16 of 19 validated off-target sites by VIVO, and 24 of 26 validated off-targets by DISCOVER-Seq. In addition, GUIDE-tag identified a small number of sites that were not captured by the other two methods. These data suggest that GUIDE-tag provides a sensitive, genome-wide method for identifying potential off-target sites in vivo. Tn5 tagmentation of the treated genome allows the same input genomic DNA to be used for UDiTaS analysis to identify large deletions and translocations at nuclease target sites. The ability to identify translocations anchored at the target site provides independent validation of potential off-target sequences beyond the traditional amplicon deep sequencing analysis^5^.

While the SpyCas9-mSA system provided a substantial increase in integration rates (4.3 fold) for large biotinylated donors (GFP cassettes) relative to 5′ large phosphorylated donors, the integration rate for short synthetic duplex oligos delivered by HTVI was only modestly improved by biotin-mediated tethering. One notable difference between these donor types is that the GUIDE-seq donor contains phosphorothioates on the 5′ and 3′ ends of the duplex, whereas the PCR-generated IRES-GFP donor lacks these modifications. 5′ modifications can interfere with NHEJ-mediated DNA donor insertion into the genome^37,39^, and the presence of phosphorothioates at the ends of the DNA may impede the removal of the 5′ biotin from the synthetic donors and thereby reduce insertion rates. Indeed, shifting the biotin from a 5′ terminal position to an internal position within the donor significantly enhanced insertion efficiency for both SpyCas9 RNP and SpyCas9-mSA RNP in cell culture, suggesting that dsODN with internal biotin may represent an improved modification for GUIDE-tag by leaving the 5′-ends of the donor accessible for NHEJ-mediated insertion. Under HTVI conditions, an additional difference between long and short donors in vivo is the molar amount of donor that is delivered: ~250-fold more short synthetic duplex is delivered than the large GFP cassettes on a molar basis, and consequently capture of a short synthetic duplex at the DBS site may be less dependent on Cas9 tethering. Thus, when duplex DNA can be efficiently delivered to cells in a target tissue in vivo or in cell culture, the SpyCas9-mSA system is unlikely to provide an appreciable advantage over standard SpyCas9 systems for tagging DSBs. In some instances, the amount of Cas9 RNP and donor DNA delivered in vivo may be limiting and subject to dilution by diffusion. Under these circumstances, the association of the Cas9-mSA and biotin-donor could be advantageous for labeling DSBs.

Intratracheal delivery of polymer-stabilized SpyCas9-mSA ribonucleoprotein complexes and biotin-dsDNA donor demonstrated a proof of concept for off-target analysis in vivo using an approach with potential therapeutic application. While enrichment of edited cells via fluorescent cell sorting was required for the assessment of off-target editing rates in the lung, it demonstrates the feasibility of this approach. We anticipate adapting this DSB tagging system to other DNA delivery methods such as nanoparticle or naked dsDNA-bearing chemical conjugates to facilitate tissue-selective uptake^65^. These donors could be used in conjunction with Cas9 nuclease delivered through a therapeutic compatible modality (AAV, nanoparticle, RNP) in additional tissue types. This envisioned next-generation GUIDE-tag system would permit the evaluation of in vivo editing in the context of a therapeutically relevant disease mouse model. While this system would not provide information on the specificity of editing in the context of the human genome, it would provide data on differences in off-target editing rates in the context of relevant cell types or organ systems that will have differences in actively transcribed genes, chromatin accessibility and differentiation state, which are difficult to mimic in cell culture. In principle, such a system could be translated to non-human primates, other representative large animal models, or mouse xenograft of human tissue^66^ to provide additional insights into the safety of therapeutic genome editing in humans.

## Methods

### Generation of plasmid

pX330 SpyCas9-mSA was a gift from Janet Rossant (Addgene plasmid # 113096). sgRNAs targeting *Actb* gene, *Fah* gene, or *Pcsk9* gene (gP from VIVO) were cloned into plasmid pX330 SpyCas9-mSA^67^. pET-45b(+)-Tn5 was a gift from Frank Pugh (Addgene plasmid # 112112) Sequences of all sgRNAs are listed in Supplementary Table 1. The modified SpyCas9-mSA* construct was generated through Gibson assembly, by combining the following four DNA fragments: (i) PCR amplified 3xc-Myc NLS fragment, (ii) PCR-amplified mammalian codon-optimized Cas9 cassette, (iii) a DNA gblock (linker-mSA-2xNLS), and (iv) AgeI/EcoRI-digested pX330 SpyCas9-mSA backbone. The SauCas9-mSA* construct was generated through Gibson assembly, by combining the following two DNA fragments: (i) a DNA gblock (linker-mSA-2xNLS), and (ii) EcoRI-digested customized pmax SauCas9 expression vector. The enAsCas12a-mSA* construct was generated through Gibson assembly, by combining the following two DNA fragments: (i) a DNA gblock (linker-mSA-2xNLS), and (ii) EcoRI-digested customized pmax enAsCas12a expression vector.

### Donors for GUIDE-tag

To generate IRES-GFP donors for *Actb* gene, a vector (Addgene plasmid # 83575) containing IRES-GFP was used as a template for PCR amplification using Phusion master mix (ThermoFisher Scientific) with either biotinylated primers or standard primers (Supplementary Table 3). To generate exon 2–14 donors for the Fah gene, a gene block (IDT) containing a 3′ splice acceptor and Fah cDNA of exon 2–14 was used as a donor for PCR amplification with either biotinylated primers or standard primers (Supplementary Table 3). iGUIDE donor^53^ was prepared by annealing the following two oligos:

5′-P-G*C*TCGCGTTTAATTGAGTTGTCATATGTTAATAACGGTATACGC*G*A and 5′-P-T*C*GCGTATACCGTTATTAACATATGACAACTCAATTAAACGCGA*G*C.

GUIDE-seq donor^4^ was prepared by annealing the following two oligos:

5′-P-G*T*TTAATTGAGTTGTCATATGTTAATAACGGT*A*T and

5′-P-A*T*ACCGTTATTAACATATGACAACTCAATTAA*A*C.

Biotin-iGUIDE donor was prepared by annealing:

5′-biotin-G*C*TCGCGTTTAATTGAGTTGTCATATGTTAATAACGGTATACGC*G*A and 5′-biotin-T*C*GCGTATACCGTTATTAACATATGACAACTCAATTAAACGCGA*G*C.

Different Biotin-GUIDE donor combinations were prepared by annealing the following oligos:

GS1: 5′-P-G*T*TTAATTGAGTTGTCATATGTTAATAACGGT*A*T,

GS2: 5′-P-A*T*ACCGTTATTAACATATGACAACTCAATTAA*A*C,

GS1-5′Bio: 5′-biotin-G*T*TTAATTGAGTTGTCATATGTTAATAACGGT*A*T, GS2-5′Bio: 5′-biotin-A*T*ACCGTTATTAACATATGACAACTCAATTAA*A*C, GS1-IntBio: 5′-P-G*T*TTAATTGAGTTGTCAT(biotin)ATGTTAATAACGGT*A*T and GS2-IntBio: 5′-P-A*T*ACCGTTATTAACATAT(biotin)GACAACTCAATTAA*A*C P is 5′ phosphorylation and * indicates phosphorothioate bond.

### Cell culture and transfection

Neuro 2A (N2A) cells were purchased from ATCC, and cells were maintained in Dulbecco’s Modified Eagle’s Medium supplemented with 10% FBS at 37° and 5% CO_2_.

For transfection-based editing experiments in N2A cells, cells were plated 30,000 cells per well in a 12-well plate. 24 h later, the cells were co-transfected with the indicated dose of SpyCas9-mSA plasmid, and biotinylated dsDNA donors. Lipofectamine 3000 (for plasmids) or CRISPRmax (for RNP) was used for the transfection according to the manufacturer’s instructions. FACS analysis was performed 4 days after transfection, and genomic DNA was isolated for PCR analysis.

For editing experiments in Hep1-6 cells, 25k Hepa1-6 cells were electroporated with 2 pmol of 3xNLS-SpyCas9 sgPcsk9 RNP or 3xNLS-SpyCas9-mSA sgPcsk9 RNP (sgRNA from IDT) and 5pmol of each different GUIDE-seq donor duplex DNA (except in the case of the RNA and donor titration experiments, where the dose range delivered is indicated in the figure legend). gDNA were isolated 3 days after transfection from each group and the insertion and indel percentages were measured by deep sequencing PCR amplicons spanning the Pcsk9 target site.

### Animal studies

All animal experiments were authorized by the Institutional Animal Care and Use Committee (IACUC) at UMASS medical school. All DNA vectors were prepared by EndoFreeMaxi kit (Qiagen).

For in vivo *Actb* gene editing, FVB/NJ (Strain #001800) mice were purchased from Jackson Laboratories. Eight-week-old mice were injected with 2–2.5 ml 0.9% saline containing 20 μg sg*Actb*-SpyCas9-mSA or sg*Actb*-SpyCas9-mSA* and 4 μg of dsDNA donor or biotinylated dsDNA donor (IRES-GFP) into the tail vein in 5–7 s.

For in vivo *Fah* gene editing, Fah^−/−^ (deltaExon5) mice were a gift from Dr. Markus Grompe (Oregon Health Science University) and were kept on 10 mg/L NTBC water. Eight-week-old mice were injected with 2–2.5 ml 0.9% saline containing 20 μg sgFah-SpyCas9-mSA and 4 μg of dsDNA donor or biotinylated dsDNA donor (Fah exon 2–14). NTBC water was removed 7 days post injection (defined as NTBC on, D0) to assess the functional correction of *Fah*. Two cycles of NTBC withdrawal (D0, D19, and D26) and reintroduction (D17 and D22) were performed to allow expansion of FAH^+^ hepatocytes.

For in vivo Pcsk9 gene editing, C57BL/6J (Strain #000664) mice were purchased from Jackson Laboratories. 15 Eight-week-old mice were randomally allocated into five groups. Mice were injected with 2–2.5 ml 0.9% saline containing (i) 1nmol of iGUIDE donor, or (ii) 1nmol of biotinylated iGUIDE donor, or (iii) 30 μg sgPcsk9 gP-SpyCas9-mSA*, or (vi) 30 μg sgPcsk9 gP-SpyCas9-mSA* and 1nmol of iGUIDE donor, or (v) 30 μg sgPcsk9 gP-SpyCas9-mSA* and 1nmol of biotinylated iGUIDE donor.

Animals were sacrificed at the end of each experiment (7 days for the *Actb* and *Pcsk9* editing). Livers were fixed with formalin or stored at −80 °C until further analyses. No animals were excluded from the analyses. No sample size calculation was performed and each group consisted of at least three mice for statistical analysis.

### Lung RNP delivery

Alt-R sgRNA (sgPcks9 and sgAi9) or Alt-R crRNA were chemically synthesized by Integrated DNA Technologies (IDT), resuspended in IDT duplex buffer at a concentration of 100 µM, and stored in aliquots at −80 °C (Supplementary Table 1). Ai9 mice were purchased from Jackson Laboratories (strain #007909). Fresh SpyCas9-mSA* RNPs or enAsCas12a RNPs were generated as previously described^59^. For each mouse, 4.5 nmol sgRNA was first mixed 1:0.8 v/v with 15–50 kDa PGA (100 mg/ml, Sigma-Aldrich) prior to complexing with 3 nmol Cas9-mSA* or Cas12a proteins for a final volume ratio of sgRNA:PGA:Cas9 of 1:0.8:1. For donor co-delivery, 1nmol bio-iGUIDE donor was mixed with RNPs (after 10 min) to form complexes. The RNP complexes were then delivered to mouse lung through intratracheal injection. tdTomato positive cells were sorted from dissociated mouse lung as previously described^68^. gDNA was isolated from ~200,000 sorted cells from each treated mouse and GUIDE-tag libraries were prepared as below.

### Whole exome sequencing (WES) and variant calling

The livers of 3 control mice that received biotin-Fah-dsDNA by HTLV and 3 mice received with sgFah-SpyCas9-mSA and biotin-Fah-dsDNA by HTLV were analyzed to determine the rate of genome-wide variants after hepatocyte expansion (at D34). 1.5ug of gDNA per mouse was used for library preparation and an average of 120 Gb of deep sequencing data (~1000×) was generated per mouse (GENEWIZ). The original downstream analysis procedure has been described previously^69^. In brief, raw reads were processed with fastqc (Version 0.11.9) and trim_galore (Version 0.6.5) (https://www.bioinformatics.babraham.ac.uk/projects/) to remove reads with low quality and trim adapters. Then processed reads were mapped to Mouse GRCm38/mm10 with BWA-mem (v0.7.15)^70^. Picard (v1.119) (https://github.com/broadinstitute/picard) was used to mark duplicated reads. Genome Analysis Toolkit (GATK; version 4.1.6.0)^70^ was used for variant calling of low-frequency SNVs and INDELS with default parameters. Final SNVs in 3 treatment mice were extracted and filtered by 3 control mice.

### Electrophoretic mobility shift assays (EMSA)

An iGUIDE sense oligonucleotide with 5′biotin and 3′Cy3 terminal modifications was purchased from IDT. To make biotin-iGUIDE-Cy3 donor duplex, 5′-biotin and 3′-Cy3 labeled iGUIDE sense oligonucleotide and anti-sense 5′-biotin iGUIDE oligonucelotide were mixed 1:1 mol/mol ratio and annealed. Ten pmol SpyCas9-mSA* (or SpyCas9 lacking mSA) in 7.5 µL was mixed at an equal molor ratio with 7.5 µL of sgRNA and incubated at room temperature for 10 min. Next, the 15 µL of Cas9-mSA* RNP was incubated with biotin-iGUIDE-Cy3 donor or a control Cy3-labeled duplex DNA lacking biotin at a 5:1 molor ratio unless otherwise indcated in a total volume of 30 µL of EMSA buffer [50 mM Tris-HCl, pH 7.0, 20 mM KCl, 1 mM MgCl_2_, 0.1% NP-40, 0.1% Tween20, 6% glycerol and 5 mM tris(2-carboxyethyl) phosphine (TCEP)]. Samples were analyzed by electrophoresis on a 4% native PAGE and DNA visualized by Cy3 fluorescence.

### Protein purification

Protein purification followed a previously described protocols for Cas9-based proteins^62^ and Cas12a-based proteins^71^. Tn5 purification utilized a modified protocol^72^ that includes the addition of PEI and (NH_4_)_2_SO_4_ precipitations. pET-45b(+)-Tn5 (for Tn5 protein, a gift from Frank Pugh - Addgene plasmid # 112112) or pET-21a-SpyCas9-mSA* (for Cas9-mSA* protein) or pET-21a-3xNLS-SpyCas9-mSA (for 3xNLS-SpyCas9-mSA protein) or pET-21a-3xNLS-SpCas9 (Plasmid #114365) or pET-21a-enAsCas12a (for enAsCas12a protein) were introduced into *E. coli* Rosetta2(DE3)pLysS cells (EMD Millipore) for protein overexpression. Cells were grown at 37 °C to an OD600 of ~0.2, then shifted to 18 °C and, at OD600 of ~0.4, induced for 16 h with IPTG (0.7 mM final concentration). Following induction, cells were pelleted by centrifugation and then resuspended with Nickel-NTA buffer (20 mM TRIS + 1 M NaCl + 20 mM imidazole + 1 mM TCEP, pH 7.5) supplemented with HALT Protease Inhibitor Cocktail, EDTA-Free (100X) [ThermoFisher] and lysed with LM-20 Microfluidizer (Microfluidics) following the manufacturer’s instructions. For Tn5 purification prior to Ni-NTA purification, the nucleic acids were removed by precipitation with 0.25% w/v PEI and centrifuged @10,000 × *g* for 10 min. The PEI was removed by precipitating the protein with 70% (NH_4_)_2_SO_4_ at 4 °C and centrifuged @12,000 × *g* for 15 min. The protein pellet was resuspended in Nickel-NTA buffer and purified with Ni-NTA resin and eluted with elution buffer (20 mM TRIS, 500 mM NaCl, 250 mM Imidazole, 10% w/v glycerol, pH 7.5). Tn5 protein was dialyzed overnight at 4 °C in 20 mM HEPES, 500 mM NaCl, 1 mM EDTA, 10% w/v (8% v/v) glycerol, pH 7.5. Subsequently, Tn5 protein was step dialyzed from 500 mM NaCl to 200 mM NaCl (Final dialysis buffer: 20 mM HEPES, 200 mM NaCl, 1 mM EDTA, 10% w/v glycerol, pH 7.5). Next, the Tn5 protein was purified by cation exchange chromatography (Column = 5 ml HiTrap-S, Buffer A = 20 mM HEPES pH 7.5 + 1 mM TCEP, Buffer B = 20 mM HEPES pH 7.5 + 1 M NaCl + 1 mM TCEP, Flow rate = 5 ml/min, CV = column volume = 5 ml). The primary protein peak from the CEC was dialyzed to 2xTn5 buffer (100 mM HEPES-KOH at pH 7.2, 0.2 M NaCl, 0.2 mM EDTA, 2 mM DTT, 0.2% Triton X-100, 20% glycerol) and concentrated in an Ultra-15 Centrifugal Filters Ultracel −30K (Amicon) to a concentration of 63.5 µM. Finally, 0.827 volumes of 100% glycerol was added for a final concentration of 55% glycerol and then the Tn5 is stored at −20 °C until needed for transposome assembly.

### Synthesis of oligonucleotides

Biotinylated oligonucleotides generated in house were synthesized at 1 µmole scale on a Biolytic Dr. Oligo 48 synthesizer. Standard phosphoramidites were purchased from ChemGenes. 5′ biotin (10–5950) and internal biotin (Biotin dT; 10–1038) phosphoramidites were purchased from Glen Research. Oxidation to phosphodiester linkages was accomplished with 0.05 M Iodine in 90% pyridine/10% water. Sulfurization to phosphorothioate linkages was accomplished with 0.1 M DDTT solution (ChemGenes). Oligonucleotides were deprotected with 30% NH_3_ in water (16 h at 55 °C), and then the ammonia was removed under vacuum. The oligonucleotides were then desalted (3x RNase-free water wash, 14 K rpm, 15 min) using Amicon Ultra 0.5 mL 3 K filters (Millipore, Billerica, MA), and resuspended in 400 μL RNase-free water. Oligonucleotides were analyzed on an Agilent 6530 Q-TOF LC/MS system with electrospray ionization and time-of-flight ion separation in negative ionization mode. Liquid chromatography was performed using a 2.1 × 50 mm AdvanceBio oligonucleotide column (Agilent Technologies, Santa Clara, CA). The data were analyzed using Agilent Mass Hunter software. Buffer A: 100 mM hexafluoroisopropanol with 9 mM triethylamine in water; Buffer B: 100 mM hexafluoroisopropanol with 9 mM trimethylamine in methanol. Samples were resolved over an elution gradient from 0 to 100% Buffer B over 5 min.

### Tn5 tagmentation and library preparation for GUIDE-tag and UDiTaS

25 mg of frozen liver tissue was lysed to isolate ~25 μg genomic DNA using DNeasy blood & tissue kits (Qiagen). Adaptor oligonucleotides were synthesized by IDT (Supplementary Table 3). Transposon assembly was done by incubating 158ug Tn5 with 1.4 nmol annealed oligo (contains the full-length Illumina forward (i5) adapter, a sample barcode, and UMI)^40^ at room temperature for 60 min.

For tagmentation, 200 ng of genomic DNA was incubated with 2 μl of assembled transposome at 55° for 7 min, and the product was cleaned up (20 μl) with a Zymo column (Zymo Research, #D4013). Tagmented DNA was used for the 1st PCR using PlatinumTM SuperFi DNA polymerase (Thermo) with i5 primer and gene-specific primers (Supplementary Table 3). Four different libraries were prepared for gDNA from each mouse with different combinations of primers (i5+Locus_F [UDiTaS], i5+Locus_R [UDiTaS], i5+Insert_F [GUIDE-tag] and i5+Insert_R [GUIDE-tag]). The i7 index was added in the 2nd PCR and the PCR product was cleaned up with Ampure XP SPRI beads (Agencourt, 0.9X reaction volume). Completed libraries were quantified by Tapestation and Qubit (Agilent), pooled with equal mole, and sequenced with 150 bp paired-end reads on an Illumina MiniSeq instrument.

### GUIDE-tag and UDiTaS data analysis

The GUIDE-tag and UDiTaS analysis pipeline was built using python code. Code is available at https://github.com/locusliu/GUIDESeq-Preprocess_from_Demultiplexing_to_Analysis and as we previously reported^72^. Briefly, it consists of the following steps:

i. Demultiplexing and UMI extraction. Raw BCL files were converted and demultiplexed using the appropriate i5 and i7 sequencing barcodes, allowing up to one mismatch in each barcode. UMIs for each read were extracted into UMI.fastq files after filtering out the UMIs containing ‘N’ for further downstream analysis.
ii. Raw reads were processed with fastqc (Version 0.11.9) and trim_galore (Version 0.6.5) (https://www.bioinformatics.babraham.ac.uk/projects/) to remove reads with low quality and trim adapters (regular Illumina adapter sequences), inserted tag (GUIDE-seq, iGUIDE) sequences, locus-specific sequences in UDiTaS (gene-specific primers) or IRES-GFP and FAH repair cassette for GUIDE-tag.
iii. For UDiTaS create a reference sequence based on the UDiTaS locus-specific primer position and donor map separately. Build index files for the reference using bowtie2-index, version 2.4.0.
iv. Alignment analysis. Paired reads were then globally aligned (end-to-end mode) to mouse genome (mm10) and all the reference amplicons using bowtie2’s very sensitive parameter. Finally, Samtools (version 0.1.19) was used to create an index-sorted bam file.
v. Data anlaysis:
  - For UDiTaS analysis at each target site, locus-specific primers were used to construct UDiTaS libraries, precise editing or small indels were analyzed as previously described^73^. Pindel (version 0.2.5b8) was used to detect breakpoints of large deletions and donor integration. Raw sequencing reads that align to the reference sequence were collapsed to a single read by common UMI and categorized as an exemplar for each UMI to a specific category—for example, Wild Type, precise editing, small indel/substitution (<50 bp), and Large Deletions/Insertions (>50 bp). Then the number of UMIs assigned per category was determined to define the ratio of each event.
  - For GUIDE-Tag, iGUIDE-Tag or long donor-based (IRES-GFP and FAH repair cassette) analysis for off-targets identification the analysis pipeline that was used is dependent on the sequence of the DNA donor that was used. For synthetic duplex donors containing the iGUIDE sequence, the data was preprocessed using iGUIDE package^53^ to remove mispriming events (https://github.com/cnobles/iGUIDE) before running through the Bioconductor GUIDE-seq analysis pipeline as previously described^74,75^. After these preprocessing steps all data were analyzed for off-target site identification through the Bioconductor GUIDE-seq analysis pipeline. Briefly, for GUIDE-seq analysis processed paired reads were merged (if they overlap) and then globally aligned to the mouse genome (mm10) using bowtie2. Then BAM files and UMI files were used to aggregate unique reads. Default parameters were used for defining peaks composed of unique reads that may represent off-target sites. Potential off-target site identification within these peaks required the presence of a near-cognate recognition sequence for Cas9 with these parameters: the maximum number of allowed mismatches is 6 positions with one DNA/RNA bulge permitted and the presence of an NNG or NGN PAM is required. The peaks that represent potential off-targets sites were extracted from the GUIDE-Tag R package output files, which have the location information, then the header and UMI sequence for each read were extracted from UMI.fastq files. Subsequently, UMI counts within peaks that represent potential off-targets sites were counted, where a UMI is required to have at least three read counts to be included to reduce UMI singletons associated with sequencing errors.

For computational prediction comparison, we used CRISPRseek^55^ to predict potential off-targets sites for sg*Actb* and sg*Fah* sgRNAs, allowing up to three mismatches.

### Targeted amplicon deep sequencing to assess editing rates

Genomic DNA was isolated for indel analysis from the frozen liver of mice injected with sgRNA+SpyCas9-mSA. For the *Actb* and Fah sgRNA, we validated all off-target sites identified by GUIDE-tag. For the Pcsk9 sgRNA, we seleted 52 off-target sites (16 overlapping sites with VIVO, 24 overlapping sites with DISCOVER-seq, 17 sites identified by CIRCLE-seq but not validated by VIVO and 6 sites identified by GUIDE-tag) for amplicon deep sequencing to verify the presence of indels. 200 ng of genomic DNA was used for PCR using Phusion master mix (Thermo) with locus specific primers. All primers used for amplicon sequence are listed in Supplementary Table 3. PCR products were purified with Ampure beads (0.9X reaction volume) and eluted with 25 μl of TE buffer, and were quantified by Tapestation and Qubit. Equal mole of each amplicon was pooled and sequenced using Illumina Miniseq. Amplicon sequencing data were analyzed with CRISPResso (https://crispresso.pinellolab.partners.org/).

### Immunohistochemistry and immunofluorescence

For immunohistochemical studies, formalin-fixed, paraffin-embedded (FFPE) mouse liver samples were sectioned at 4 μm, deparaffinized, and subsequently stained with anti-GFP (1:200, CST, Cat. #2956) or anti-Fah antibody (1:100, Abcam, Cat. #83770). Visualization was performed using the DAB Quanto kit (Fisher Scientific, Cat. # TA-125-QHDX) as instructed by the manufacturer.

For immunofluorescence, N2A cells grown on coverslides were fixed with 4% paraformaldehyde for 15 min at room temperature (RT) and permeabilized with 0.1% Triton X-100/PBS at RT for 15 min. Cells were then incubated overnight at 4 °C with anti-streptavidin antibody (1:100, Vector, Cat. # BA-0500-.5) and 1 h at room temperature with Alexa Fluor 647 Donkey anti-goat IgG (Invitrogen, Cat. #A32849). Nuclei were counterstained with DAPI. Images were acquired on a Leica DMi8 imaging microscope.

### Statistical analysis

Statistical analyses for plotted data were performed using GraphPad Prism 8.4. Sample size was not pre-determined by statistical methods, but rather, based on preliminary data. Group allocation was performed randomly. In all studies, data represent biological replicates (*n*) and are depicted as mean ± s.d. as indicated in the figure legends. Comparison of mean values was conducted with unpaired, two-tailed Student’s t-test; one-way ANOVA; or two-way ANOVA with Tukey’s multiple comparisons test, as indicated in the figure legends. R (version 3.4.3), a system for statistical computation and graphics, was used for the analysis of the significance of Indel and translocation rates^76^. Indel frequency and translocation rate were first arcsine transformed to homogenize the variance. For the experiment with more than two groups (Actin), Levene’s test indicates that the assumption of homogeneity of variances was met for the on-target and all off-targets. Therefore, one-way analysis of variance (ANOVA) with completely randomized design was performed followed by pre-specified contrasts for the on-target and each off-target. For experiments with two groups (Fah and Pcsk9), Welch two sample t-test was performed for the on-target and each off-target. *P* values were adjusted using the Benjamini & Hochberg (BH) method to correct for multiple inferences in each experiment^77^. Correlation coefficient (Spearman and Pearson) were analyzed using R (version 3.4.3). In all analyses, *P* values < 0.05 were considered statistically significant.

### Reporting summary

Further information on research design is available in the Nature Research Reporting Summary linked to this article.

## Supplementary information

Supplementary information

Reporting Summary

Description of Additional Supplementary Files

Supplementary Data 1

Supplementary Data 2

Supplementary Data 3

Supplementary Data 4

Supplementary Data 5

Supplementary Data 6

Supplementary Data 7

Supplementary Data 8

## Data Availability

The next-generation sequencing data have been deposited in the NCBI Sequence Read Archive database under the bioProject accession code PRJNA726835. All other relevant data are available from corresponding authors upon request. Source data are provided as a Source Data file. Source data are provided with this paper.

## Source data

Source data file
