# Supplementary material for: Genome-wide detection of CRISPR editing in vivo using GUIDE-tag
Source: Nat Commun. 2022 Jan 21;13:437. doi: 10.1038/s41467-022-28135-9 (PMC8782884; doi:10.1038/s41467-022-28135-9)

Supplementary Information

Genome-wide Detection of CRISPR editing *in vivo* using GUIDE-tag

Shun-Qing Liang[1¶], Pengpeng Liu[2¶], Jordan L. Smith[1], Esther Mintzer[2], Stacy Maitland[2], Xiaolong Dong[1], Qiyuan Yang[2], Jonathan Lee[1], Cole M. Haynes[2], Lihua Julie. Zhu[2,3,4], Jonathan K. Watts[1], Erik J. Sontheimer[1,3,5], Scot A. Wolfe[2,5*], Wen Xue[1,2,3,5*]

[1]RNA Therapeutics Institute, University of Massachusetts Medical School, Worcester, MA, USA;

[2]Department of Molecular, Cell and Cancer Biology, University of Massachusetts Medical School, Worcester, MA, USA.

[3]Department of Molecular Medicine, University of Massachusetts Medical School, Worcester, Massachusetts, USA.

[4]Program in Bioinformatics and Integrative Biology, University of Massachusetts Medical School, Worcester, Massachusetts, USA.

[5]Li Weibo Institute for Rare Diseases Research, University of Massachusetts Medical School, Worcester, MA, USA

[¶]Equal contribution to this study

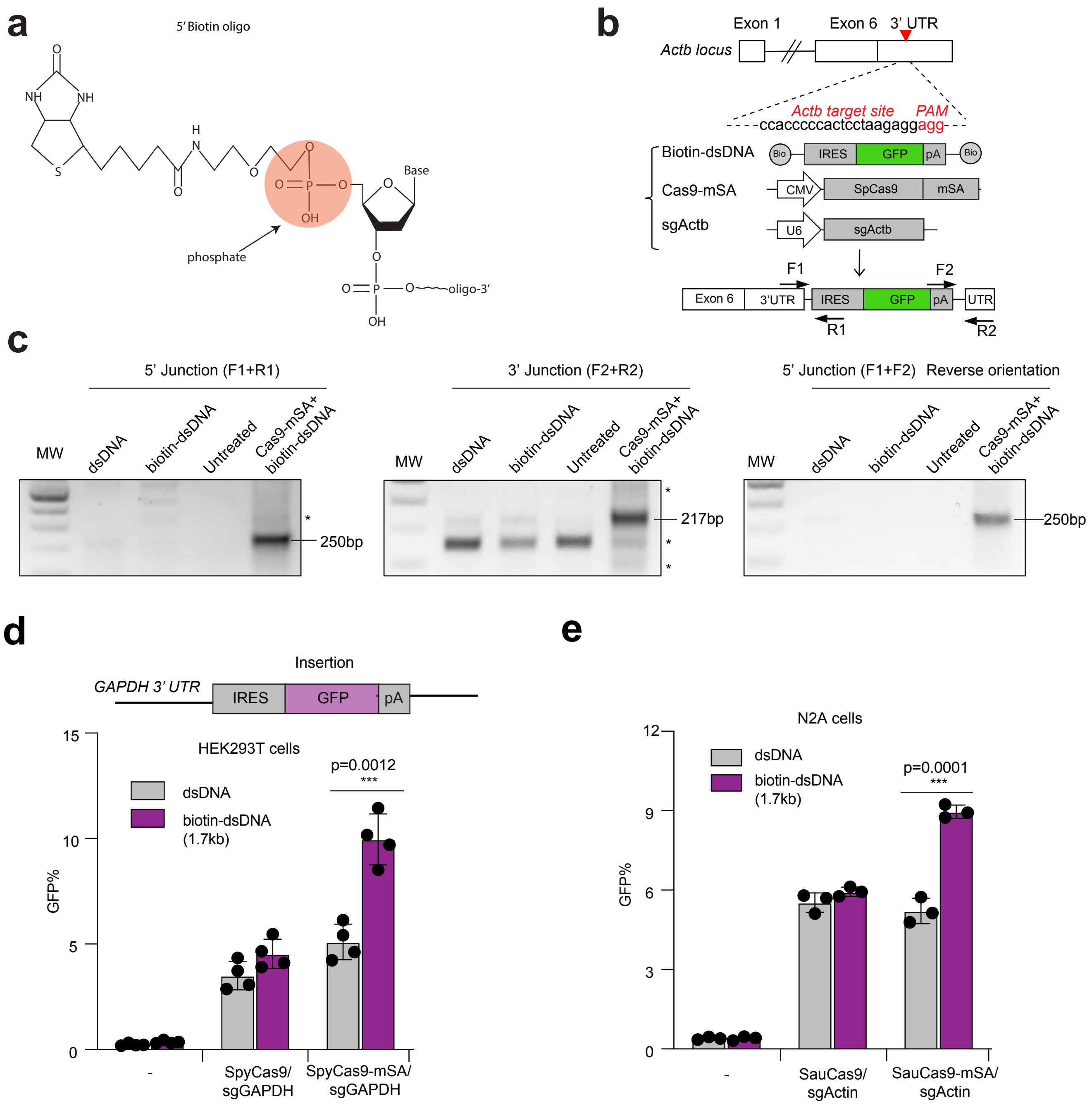
a
5' Biotin oligo
phosphate
Base
oligo-3'
b
Exon 1
Exon 6
3' UTR
Actb locus
Actb target site
PAM
ccacccccactcctaagaggagg
Biotin-dsDNA
Bio
IRES
GFP
pA
Cas9-mSA
CMV
SpCas9
mSA
sgActb
U6
F1
F2
Exon 6
3'UTR
UTR
R1
R2
c
5' Junction (F1+R1)
3' Junction (F2+R2)
5' Junction (F1+F2) Reverse orientation
MW
dsDNA
biotin-dsDNA
Untreated
Cas9-mSA+ biotin-dsDNA
250bp
217bp
d
Insertion
GAPDH 3' UTR
HEK293T cells
dsDNA
biotin-dsDNA (1.7kb)
p=0.0012
***
GFP%
-
SpyCas9/ sgGAPDH
SpyCas9-mSA/ sgGAPDH
e
N2A cells
p=0.0001
SauCas9/ sgActin
SauCas9-mSA/ sgActin

f

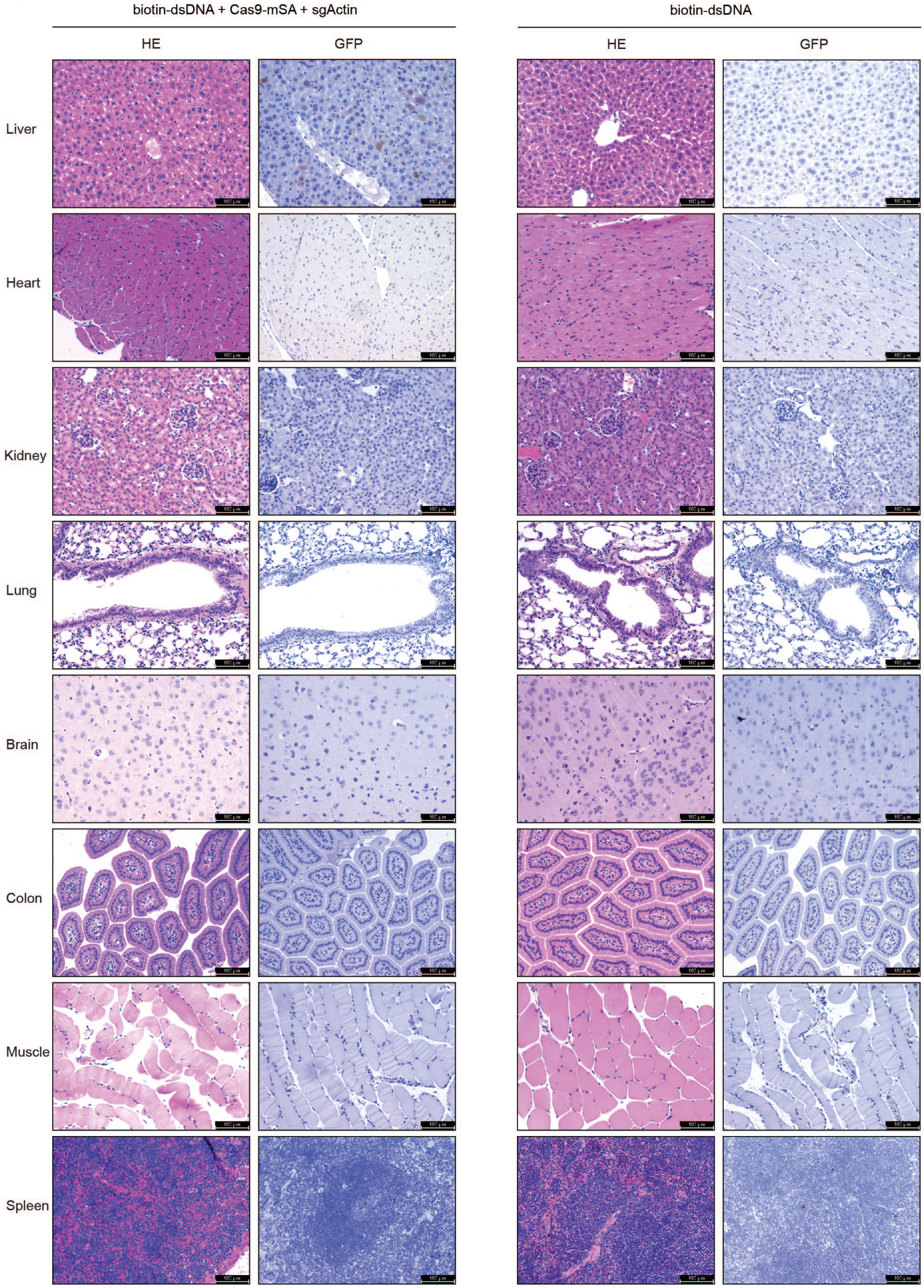
biotin-dsDNA + Cas9-mSA + sgActin
biotin-dsDNA
HE
GFP
HE
GFP
Liver
Heart
Kidney
Lung
Brain
Colon
Muscle
Spleen

Supplementary Figure 1. Tethering SpyCas9-mSA with biotin-dsDNA donor enables efficient cassette insertion in N2A cells.

a, Structure of 5'biotin modified oligo from IDT.

b, Schematic of *Actb* locus target site and editing constructs used for cassette insertion. Bracket indicates expression constructs and tagging DNA introduced by HTVI.

c, Genomic DNA was collected from N2A cells transfected with biotin-donor and Cas9-mSA/sgRNA expression vector or controls. PCR was performed with indicated primers in (b) to amplify the 5' junction sites and 3' junction site for insertions in different orientations. Results were obtained from four independent experiments. The size of expected PCR bands is indicated. * denotes unspecific bands. MW, molecular weight.

d, SpyCas9-mSA increases biotin-dsDNA donor insertion rate at GAPDH 3' UTR. 293T cells were transfected with IRES-GFP donor (dsDNA or biotin-dsDNA) with nucleases (SpyCas9/sgRNA or SpyCas9-mSA/sgRNA expression vector). Flow cytometry analysis was performed 4 days after transfection. Results were obtained from four independent experiments and presented as mean ± SEM. ***P<0.001 by one-way ANOVA with Tukey's multiple comparisons test.

e, Mouse N2A cells were transfected with IRES-GFP donor (dsDNA or biotin-dsDNA) in the absence or presence of various nucleases (SauCas9/sgRNA or SauCas9-mSA/sgRNA expression vector). Flow cytometry analysis was performed 4 days after transfection. Biotin-dsDNA with SauCas9-mSA increases GFP+ cell % compared to unmodified dsDNA. Results were obtained from three independent experiments and presented as mean ± SEM. ***P<0.001 by two-way ANOVA with Tukey's multiple comparisons test. "-" denotes donor only controls.

f, Treatment with Biotin-dsDNA with SpyCas9-mSA increases insertion efficacy compared to unmodified dsDNA in liver. Hematoxylin and eosin, and immunohistochemistry analysis (GFP) of mouse tissue at day 7 after injection. Mice (n=3 per group) were treated with either biotin-dsDNA donor (4μg) or biotin-donor (4μg) with SpyCas9-mSA/sg*Actb* (20μg). Scale bar is 100 μm.

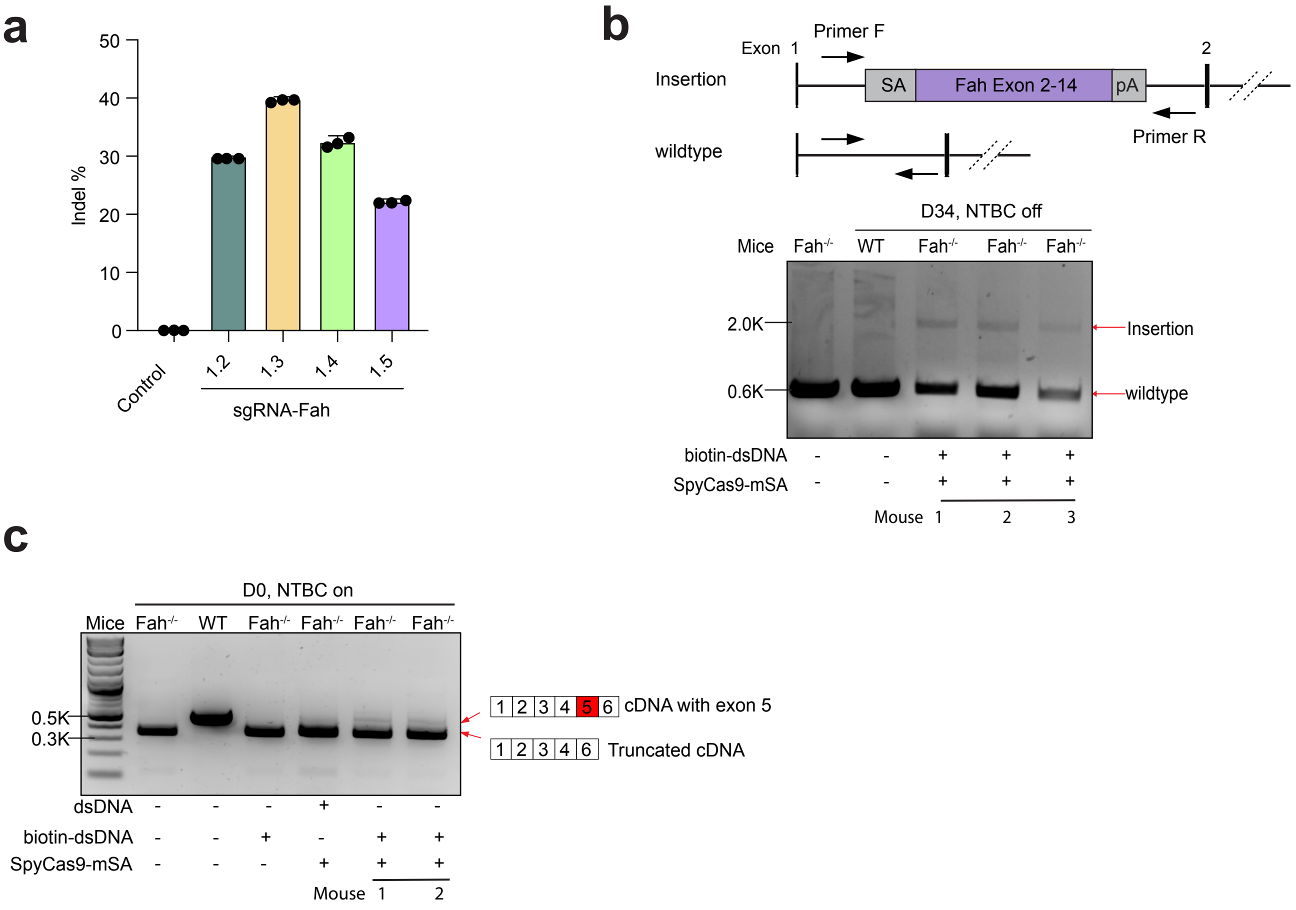
a
Indel %
50
40
30
20
10
0
Control
1.2
1.3
1.4
1.5
sgRNA-Fah
b
Exon 1
Primer F
2
Insertion
SA
Fah Exon 2-14
pA
Primer R
wildtype
D34, NTBC off
Mice Fah-/- WT Fah-/- Fah-/- Fah-/-
2.0K
0.6K
Insertion
wildtype
biotin-dsDNA - - + + +
SpyCas9-mSA - - + + +
Mouse 1 2 3
c
D0, NTBC on
Mice Fah-/- WT Fah-/- Fah-/- Fah-/- Fah-/-
0.5K
0.3K
1 2 3 4 5 6 cDNA with exon 5
1 2 3 4 6 Truncated cDNA
dsDNA - - - + - -
biotin-dsDNA - - + - + +
SpyCas9-mSA - - - + + +
Mouse 1 2

Supplementary Figure 2. Insertion of Fah exon 2-14 repair cassette in mouse liver.

a, Tracking of indels by DEcomposition (TIDE) analysis of SpyCas9 editing efficiency for 4 different sgRNAs targeting intron 1 of *Fah*. sg1.3 was chosen for *in vivo* analysis. Results were obtained from three independent experiments and presented as mean ± SEM.

b, Genomic DNA was collected from NTBC off D34 mice. PCR was perfomed with indicated primers to amplify the genomic locus targeted for cassette insertion. The upper 2kb bands (n=3 mice) represent either the forward or reverse insertion of *Fah* donor.

c, RT-PCR detects a small fraction of *Fah* transcripts containing exon 5 in NTBC on D0 mice (n=2). mRNA was collected from mice injected with indicated reagent combinations. Fah RT-PCR was performed using primers spanning exon 1 to exon 6.

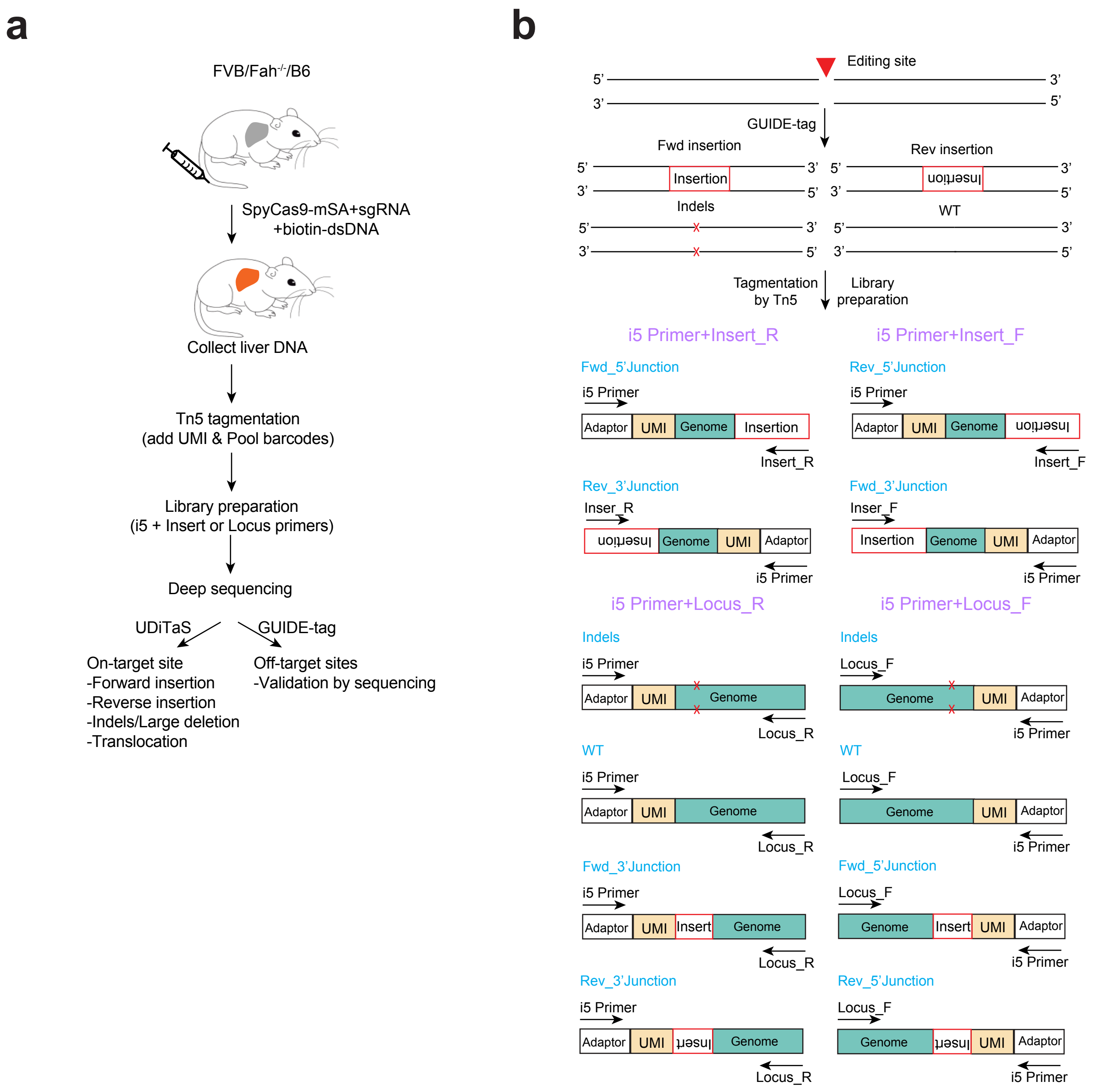

a
FVB/Fah-/-/B6
SpyCas9-mSA+sgRNA
+biotin-dsDNA
Collect liver DNA
Tn5 tagmentation
(add UMI & Pool barcodes)
Library preparation
(i5 + Insert or Locus primers)
Deep sequencing
UDiTaS
GUIDE-tag
On-target site
-Forward insertion
-Reverse insertion
-Indels/Large deletion
-Translocation
Off-target sites
-Validation by sequencing
b
Editing site
GUIDE-tag
Fwd insertion
Rev insertion
Insertion
Indels
WT
Tagmentation
by Tn5
Library
preparation
i5 Primer+Insert_R
i5 Primer+Insert_F
Fwd_5'Junction
Rev_5'Junction
i5 Primer
Adaptor
UMI
Genome
Insert_R
Insert_F
Rev_3'Junction
Fwd_3'Junction
Inser_R
Inser_F
i5 Primer+Locus_R
i5 Primer+Locus_F
Locus_F
Locus_R
Insert

Supplementary Figure 3. Overview of GUIDE-tag and UDiTaS.

a, Schematic of GUIDE-tag and UDiTaS procedure that was performed in three different mouse strains using SpyCas9-mSA with different sgRNAs and different biotinylated donor DNAs. Genomic DNA was isolated and GUIDE-tag or UDiTaS libraries was prepared (see Methods).

b, Schematic overview of GUIDE-tag and UDiTaS amplification methods to create Illumina sequencing libraries, which differ based on the primer viewpoint that is utilized (tag-specific or locus specific). Target regions are amplified with i5 Primer and target-specific primers: Insertion forward or reverse (Insert_F and R) for GUIDE-tag, or locus genomic forward or reverse (Locus_F or R) for UDiTaS.

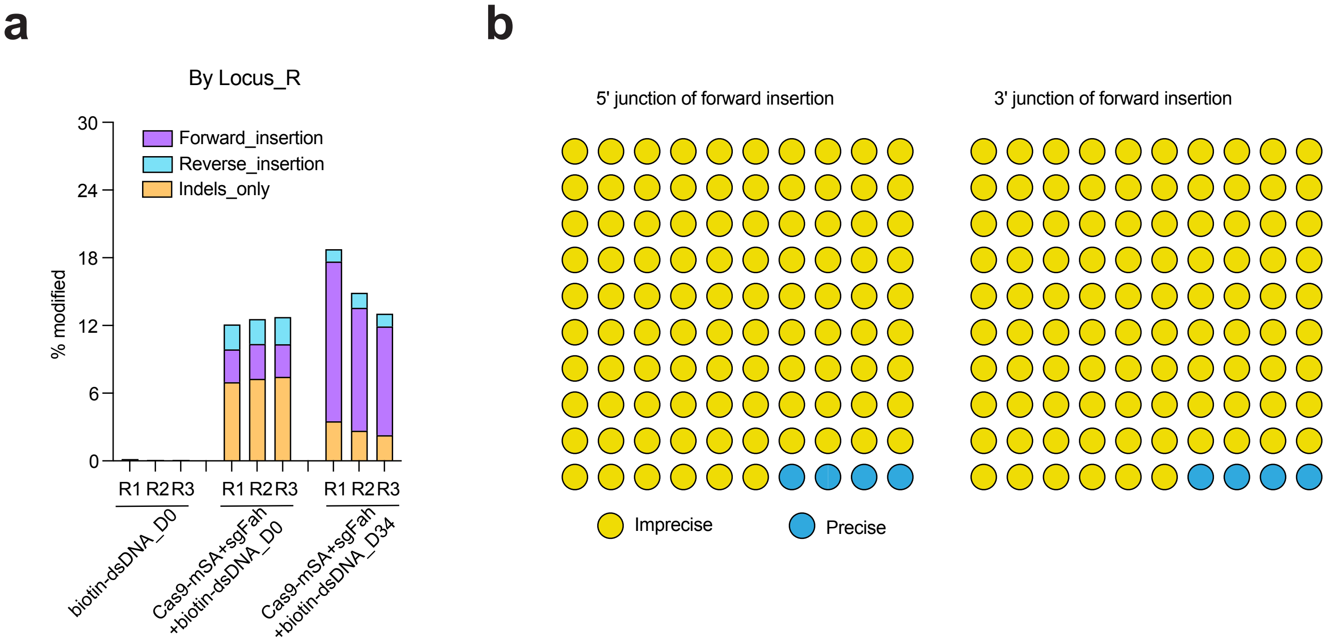

a
By Locus_R
Forward_insertion
Reverse_insertion
Indels_only
% modified
30
24
18
12
6
0
R1 R2 R3
R1 R2 R3
R1 R2 R3
biotin-dsDNA_D0
Cas9-mSA+sgFah +biotin-dsDNA_D0
Cas9-mSA+sgFah +biotin-dsDNA_D34
b
5' junction of forward insertion
3' junction of forward insertion
Imprecise
Precise

c

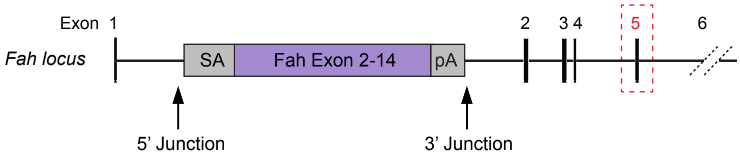

Reference Sequence

5′ AATGACTTAATACAATTGCTAGGCCTTGTTCACATATAACTAGGCAGATAATGCCTATGATCAGGTCAGAGAGGAC 3′

Fah donor sequence

5′ TTGTCTCAAAGGAAACCATGAGTCCCTAAGTATTTGCTGTTATAGTAAATGTG...................TAAACAAGTTAACAACAACAATTGCATTCATTTTATGTTTCAGGTTCAGGGGG 3′

Splice Acceptor

AATGACTTAATACAATTGCTAGGCCTTGTTCACATATAACTAGGCAGATAATGCCTATGATCAGGTCAGAGAGGAC WT 85.3%

Indels

AATGACTTAATACAATTGCTAGGCCTTGTTCACATATA-CTAGGCAGATAATGCCTATGATCAGGTCAGAGAGGAC	-1	2.98%
AATGACTTAATACAATTGCTAGGCCTTGTTCACATATAaACTAGGCAGATAATGCCTATGATCAGGTCAGAGAGGAC	+1	0.95%
AATGACTTAATACAATTGCTAGGCCTTGTTCACATA---CTAGGCAGATAATGCCTATGATCAGGTCAGAGAGGAC	-3	0.77%
AATGACTTAATACAATTGCTAGGCCTTGTTCACATA--ACTAGGCAGATAATGCCTATGATCAGGTCAGAGAGGAC	-2	0.56%
AATGACTTAATACAATTGCTAGGCCTTGTTCAC-------TAGGCAGATAATGCCTATGATCAGGTCAGAGAGGAC	-7	0.36%
AATGACTTAATACAATTGCTAGGCCTTGTTCACATA------GGCAGATAATGCCTATGATCAGGTCAGAGAGGAC	-6	0.33%
AATGACTTAATACAATTGCTAGGCCTTGTTCACA----ACTAGGCAGATAATGCCTATGATCAGGTCAGAGAGGAC	-4	0.28%
AATGACTTAATACAATTGCTAGGCCTTGT---------ACTAGGCAGATAATGCCTATGATCAGGTCAGAGAGGAC	-9	0.21%
AATGACTTAATACAATTGCTAGGCCTTGTTCACATATA--TAGGCAGATAATGCCTATGATCAGGTCAGAGAGGAC	-2	0.18%
AATGACTTAATACAATTGCTAGGCCTTGTTCACATATA----GGCAGATAATGCCTATGATCAGGTCAGAGAGGAC	-4	0.18%
AATGACTTAATACAATTGCTAGGCCTTGTTCACATATAAaaCTAGGCAGATAATGCCTATGATCAGGTCAGAGAGGAC	+2	0.15%
AATGACTTAATACAATTGCTAGGCCTTGTT--------ACTAGGCAGATAATGCCTATGATCAGGTCAGAGAGGAC	-8	0.14%
AATGACTTAATACAATTGCTAGGC---------------------AGATAATGCCTATGATCAGGTCAGAGAGGAC	-21	0.12%
AATGACTTAATACAATTGCTAGGCCTTGTTCA-------------------------------GGTCAGAGAGGAC	-31	0.08%
AATGACTTAATACAATTGCTAGGCCTTGTTCACATATAA------------TGCCTATGATCAGGTCAGAGAGGAC	-12	0.05%

5' junction site

AATGACTTAATACAATTGCTAGGCCTTGTTCACATATATTGTCTCAAAGGAAACCATGAGTCCCTAAGTATTTGCTGTTATAGTAAAT	precise	0.08%
AATGACTTAATACAATTGCTAGGCCTTG-------------TCTCAAAGGAAACCATGAGTCCCTAAGTATTTGCTGTTATAGTAAAT	imprecise	0.73%
AATGACTTAATACAATTGCTAGGCCTTGTTCACATA--TTGTCTCAAAGGAAACCATGAGTCCCTAAGTATTTGCTGTTATAGTAAAT	imprecise	0.51%
AATGACTTAATACAATTGCTAGGCCTTGTTCACATATAagcaatTTGTCTCAAAGGAAACCATGAGTCCCTAAGTATTTGCTGTTA	imprecise	0.44%
AATGACTTAATACAATTGCTAGG-----------------------------TGAGTCCCTAAGTATTTGCTGTTATAGTAAAT	imprecise	0.38%
AATGACTTAATACAATTGCTAGGCCTTGTTCA---------------------GAGTCCCTAAGTATTTGCTGTTATAGTAAAT	imprecise	0.22%
AATGACTTA---TCCCTAAGTATTTGCTGTTATAGTAAAT	imprecise	0.18%
AATGACTTAATACAATTGCTAGGCCTTGTTCACATATAcatTTGTCTCAAAGGAAACCATGAGTCCCTAAGTATTTGCTGTTATAGTAAAT	imprecise	0.16%
AATGACTTAATACAATTGCT----------------------------AAACCATGAGTCCCTAAGTATTTGCTGTTATAGTAAAT	imprecise	0.12%
AATGACTTAATACAATTGCTAGGCCTTGTTCACATATAattaTTGTCTCAAAGGAAACCATGAGTCCCTAAGTATTTGCTGTTATAGTAA	imprecise	0.12%
AATGACTTAATACAATTGCTAGGCCTTG---------------------------------------CTGTTATAGTAAAT	imprecise	0.08%
AATGACTTAATACAATTGCTAGGCCTTGTTCAC------------GGAAACCATGAGTCCCTAAGTATTTGCTGTTATAGTAAAT	imprecise	0.06%
AATGACTTAATACAATTGCTAGGC-----------------------GAAACCATGAGTCCCTAAGTATTTGCTGTTATAGTAAAT	imprecise	0.05%

Splice Acceptor

3' junction site

AACAAGTTAACAACAACAATTGCATTCATTTTATGTTTCAGGTTCAGGGGGACTAGGCAGATAATGCCTATGATCAGGTCAGAGAGGA	precise	0.07%
AACAAGTTAACAACAACAATTGCATTCATTTTATGTTTCAGGTTCAGGGGGactACTAGGCAGATAATGCCTATGATCAGGTCAGAGAGGA	imprecise	0.66%
AACAAGTTAACAACAACAATTGCATTCATTTTATGTTTCAGGTTC------ACTAGGCAGATAATGCCTATGATCAGGTCAGAGAGGA	imprecise	0.41%
AACAAGTTAACAACAACAATTGCATTCATTTTATGTTTCAGGTTCAGGGGacACTAGGCAGATAATGCCTATGATCAGGTCAGAGAGGA	imprecise	0.32%
AACAAGTTAACAACAACAATTGCATTCATTTTATGTTTCAGGTTCAGG---ACTAGGCAGATAATGCCTATGATCAGGTCAGAGAGGA	imprecise	0.26%
AACAAGTTAACAACAACAATTGCATTCATTTTATGTTTCAGGTTCAGG---------------ATAATGCCTATGATCAGGTCAGAGAGGA	imprecise	0.23%
AACAAGTTAACAACAACAATTGCATTCATTTTATGTTTCAGGTTCAGGGtactttACTAGGCAGATAATGCCTATGATCAGGTCAGAGAGGA	imprecise	0.19%
AACAAGTTAACAACAACAATTGCATTCATTTTATGTTTCAGGTTC----------AGGCAGATAATGCCTATGATCAGGTCAGAGAGGA	imprecise	0.15%
AACAAGTTAACAACAACAATTGCATTCATTTTATGTTTCAGGTTCAGGG---------------------------------------CAGAGAGGA	imprecise	0.12%
AACAAGTTAACAACAACAATTGCATTCATTTTATGTTTCAGGTTCAGGGGGgagACTAGGCAGATAATGCCTATGATCAGGTCAGAGA	imprecise	0.08%
AACAAGTTAACAACAACAATTGCATTCATTTTATGTTTCAGGTTCAGGGG------------------------ATGATCAGGTCAGAGAGGA	imprecise	0.06%
AACAAGTTAACAACAACAATTGCATTCATTTTATGTTTCAGGTTCAGGGGGcttaaggaatggACTAGGCAGATAATGCCTATGATCAGG	imprecise	0.05%

Supplementary Figure 4. Deep sequencing analyses of 5’ and 3’ junctions of the insertion sites for Fah exon 2-14.

a, UDiTaS analyses (Locus_R primer) of editing at *Fah* locus. Genomic DNA was collected from NTBC on D0 and off D34 mice. R1, R2 and R3 are three mice.

b, Percentage of imprecise and precise insertion at 5′ and 3′ junction sites at *Fah* intron 1.

c, Sequences of the indels, 5′ and 3′ junction sites in *Fah* gene identified by UdiTaS after Fah exon 2-14 insertion. The sgRNA target sequence is underlined and PAM sequence is labeled in red. The black line indicates the Cas9 cleavage site. The sequence highlighted in pink indicates the donor sequence. The percentage listed to the right of each sequence is the UMI number associated with each sequence divided by total UMIs recovered from the library.

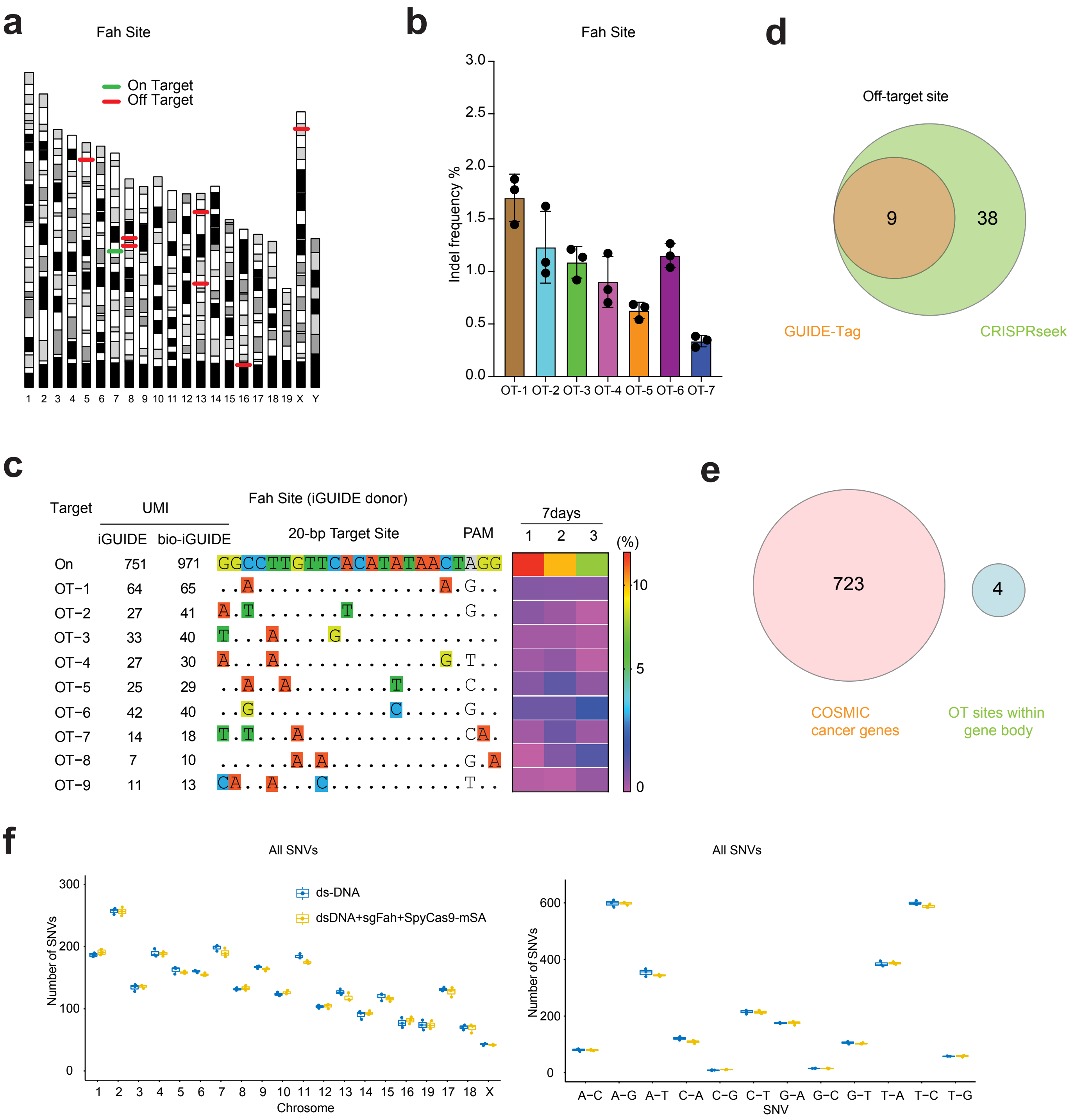
a
Fah Site
On Target
Off Target
1 2 3 4 5 6 7 8 9 10 11 12 13 14 15 16 17 18 19 X Y
b
Fah Site
Indel frequency %
3.0
2.5
2.0
1.5
1.0
0.5
0.0
OT-1 OT-2 OT-3 OT-4 OT-5 OT-6 OT-7
d
Off-target site
9
38
GUIDE-Tag
CRISPRseek
c
Target
UMI
iGUIDE
bio-iGUIDE
Fah Site (iGUIDE donor)
20-bp Target Site
PAM
7days
1 2 3
(%)
On 751 971 GGCCTTGTTCACATATAACTAGG
OT−1 64 65
OT−2 27 41
OT−3 33 40
OT−4 27 30
OT−5 25 29
OT−6 42 40
OT−7 14 18
OT−8 7 10
OT−9 11 13
10
5
0
e
723
4
COSMIC cancer genes
OT sites within gene body
f
All SNVs
ds-DNA
dsDNA+sgFah+SpyCas9-mSA
Number of SNVs
Chrosome
All SNVs
Number of SNVs
SNV
A−C A−G A−T C−A C−G C−T G−A G−C G−T T−A T−C T−G

Supplementary Figure 5. *in vivo* off-target analysis of sgFah by GUIDE-tag.

a, chromosome diagram of GUIDE-tag on-target (green) and off-target (red) cassette insertions for SpyCas9-mSA sgFah with Fah exon 2-14 repair cassette.

b, indels frequency at Fah off-target sites (OT1-7) determined by targeted amplicon deep sequencing. Black dots (n=3) represent individual mice. Error bars are SEM.

c, *in vivo* off-target sites (OT1-9) identified by GUIDE-tag with iGUIDE/biotin-iGUIDE donor at Fah site. Mismatches to the Fah target site are shown in colored boxes. Indel frequencies determined by targeted amplicon sequencing from the liver of three mice are presented as a heat map (1,2,3 are three mice). Indel frequencies determined by targeted amplicon sequencing from the liver of three mice seven days after injection are presented as heat map.

d, Venn diagram of overlapping off-target sites identified by GUIDE-tag (9 validated loci) and CRISPRseek for sgFah.

e, Overlap between identified sgFah OT sites and COSMIC cancer gene list. Of the 9 sgFah OT sites, 4 sites are within gene body (all 4 are in introns). None of the 4 Fah intragenic OT sites overlap with known cancer genes (723 cancer genes in COSMIC v92).

f, Numbers and types of SNVs identified by WES in the biotin-dsDNA and sgFah+biotin-dsDNA+SpyCas9-mSA group. Each dot (n=3) represent individual mice. In the boxes, the top, middle and bottom lines represent the 25, 50 and 75 percentiles, respectively. Whiskers indicated the min and max percentiles and outliers are not shown.

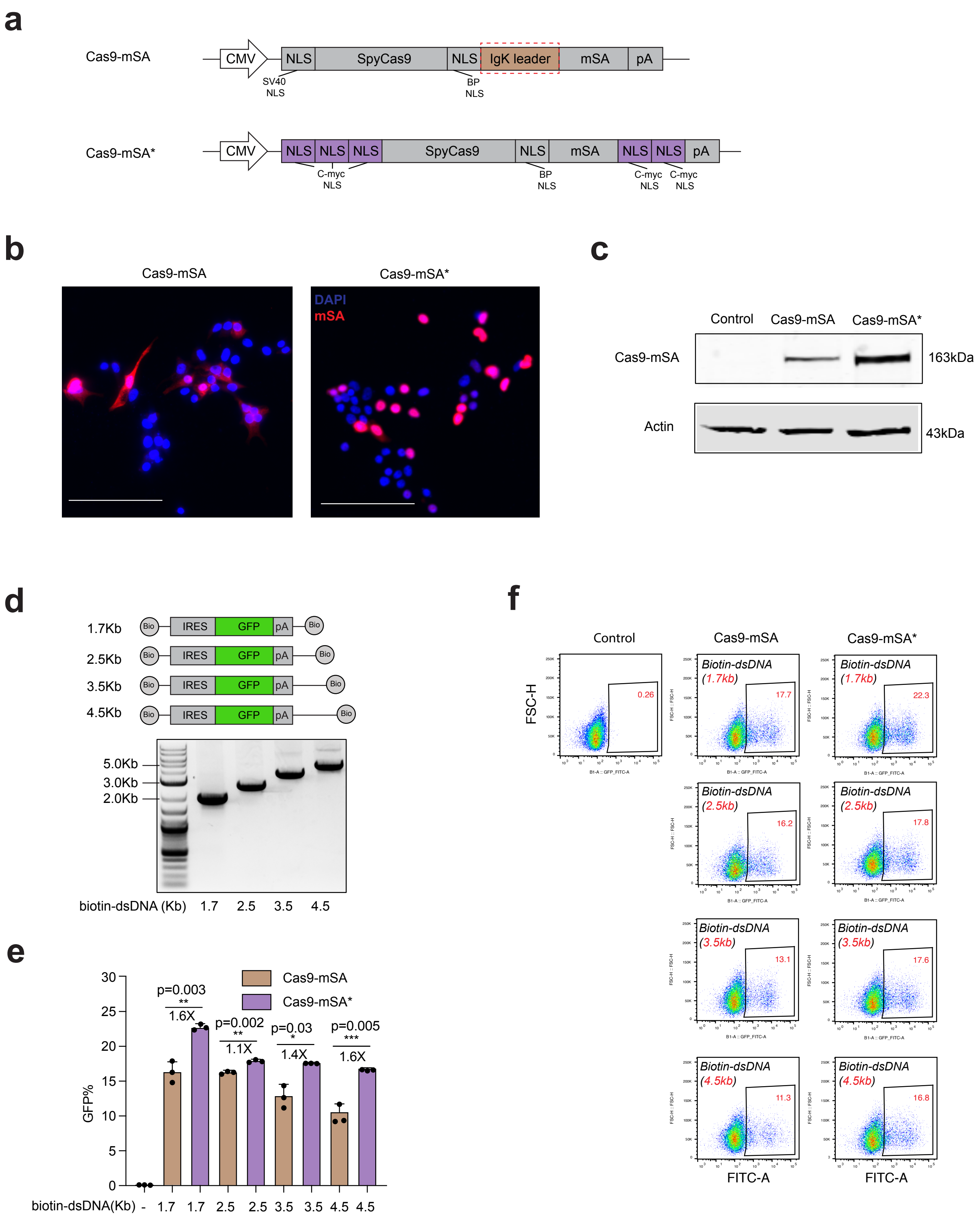

a
Cas9-mSA
CMV
NLS
SpyCas9
NLS
IgK leader
mSA
pA
SV40 NLS
BP NLS
Cas9-mSA*
CMV
NLS NLS NLS
SpyCas9
NLS
mSA
NLS NLS
pA
C-myc NLS
BP NLS
C-myc NLS
C-myc NLS
b
Cas9-mSA
Cas9-mSA*
DAPI
mSA
c
Control
Cas9-mSA
Cas9-mSA*
Cas9-mSA
163kDa
Actin
43kDa
d
1.7Kb
2.5Kb
3.5Kb
4.5Kb
Bio
IRES
GFP
pA
5.0Kb
3.0Kb
2.0Kb
biotin-dsDNA (Kb) 1.7 2.5 3.5 4.5
e
Cas9-mSA
Cas9-mSA*
GFP%
p=0.003 ** 1.6X
p=0.002 ** 1.1X
p=0.03 * 1.4X
p=0.005 *** 1.6X
biotin-dsDNA(Kb) - 1.7 1.7 2.5 2.5 3.5 3.5 4.5 4.5
f
Control
Cas9-mSA
Cas9-mSA*
FSC-H
0.26
Biotin-dsDNA (1.7kb)
17.7
22.3
Biotin-dsDNA (2.5kb)
16.2
17.8
Biotin-dsDNA (3.5kb)
13.1
17.6
Biotin-dsDNA (4.5kb)
11.3
16.8
FITC-A
FITC-A

Supplementary Figure 6. Optimization of SpyCas9-mSA improves insertion efficiency

a, Schematic representation of original SpyCas9-mSA and optimized SpyCas9-mSA*. In SpyCas9-mSA*, the Igk leader sequence was removed. Mammalian codon-optimized SpyCas9 and additional nuclear localization sequences were introduced.

b, N2A cells were transfected with original SpyCas9-mSA (300ng) or SpyCas9-mSA* (300ng). Cells were subsequently stained with antibodies against mSA (Red) and DAPI (blue; nuclei). Scale bar (white): 100 μm. Results were obtained from four independent experiments.

c, Immunoblots of N2A cells transfected with SpyCas9-mSA and SpyCas9-mSA*. mSA antibody was used for detection of SpyCas9-mSA. Results were obtained from four independent experiments.

d, Different lengths of biotinylated IRES-GFP donors were generated by one-step PCR using biotinylated primers (Supplementary table 2). Results were obtained from four independent experiments.

e, SpyCas9-mSA* improved insertion efficiency of biotinylated IRES-GFP donor cassettes. FACS analysis of functional cassette insertion events in N2A cells treated with either biotin-donor + SpyCas9-mSA, or biotin-donor + SpyCas9-mSA* for the *Actb* locus. N2A cells plated in 12-well plate (30000/well) were transfected with 300ng of SpyCas9-mSA or SpyCas9-mSA*, and 270fmol of each biotin-dsDNA. Flow cytometry analysis was performed 4 days after transfection. GFP^{+}% are presented as mean ± s.d. (n=3). ***P*<0.01, ****P*<0.001 by one-way ANOVA with Tukey's multiple comparisons test. Fold change (SpyCas9-mSA* / SpyCas9-mSA) is indicated above the bars for each cassette length.

f, Representative flow cytometry plots in **e**.

a

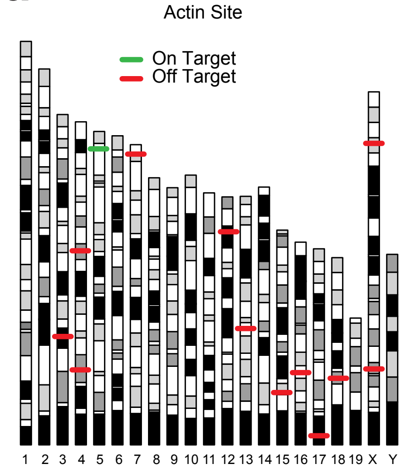

b

Actin Site

Target	UMI Cas9-mSA	UMI Cas9-mSA*	20-bp Target Site / PAM	Indel % Cas9-mSA	Indel % Cas9-mSA*
On	1595	1902	CCACCCCCACTCCTAAGAGGAGG	4.39	5.88
OT–1	1237	1550	....	4.93	5.73
OT–2	1136	1572	....	2.67	3.86
OT–4	94	70	..........–............	0.20	0.28
OT–5	72	64	............A.....A....	1.11	1.39
OT–6	20	23	G...A..................	1.21	1.62
OT–8	7	13	.........T.T...........	0.32	0.60
OT–9	3	5	..............C..–.....	0.25	0.37
OT–10	3	9	............A........A.	0.45	0.72
OT–11	2	4	....T.........T.....CA.	0.26	0.52
OT–12	2	3	.....A........T.....AA.	0.14	0.38

c **d**

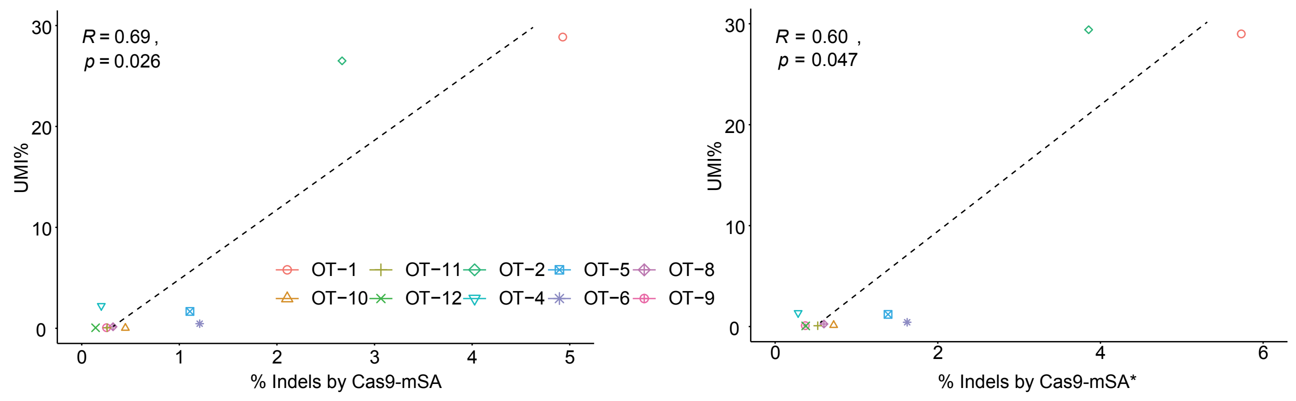

Supplementary Figure 7. *In vivo* off-target analysis of *sgActb*.

a, chromosome diagram of GUIDE-tag on-target (green) and off-target (red) insertions sites determined for SpyCas9-mSA with sg*Actb*.

b, *in vivo* off-target sites (OT1-12) identified by GUIDE-tag with biotin-IRES-GFP donor at *Actb* site that have statistically significant indel rates. Mismatches to the *Actb* target site are shown in colored boxes. Average UMI (n=3 mice) numbers for each site are shown. Indel frequencies determined by targeted amplicon sequencing from the liver of three mice are indicated (average of three mice).

c-d, Scatter plots of UMI% by GUIDE-tag with Cas9-mSA (c) or Cas9-mSA* (d) and indel frequency by amplicon sequencing for off-target cleavage sites. Dashed lines represent the linear regression fit (Spearman's correlation calculated). The p-value for Pearson's correlation coefficient was determined by the two-tailed t-distribution table.

a

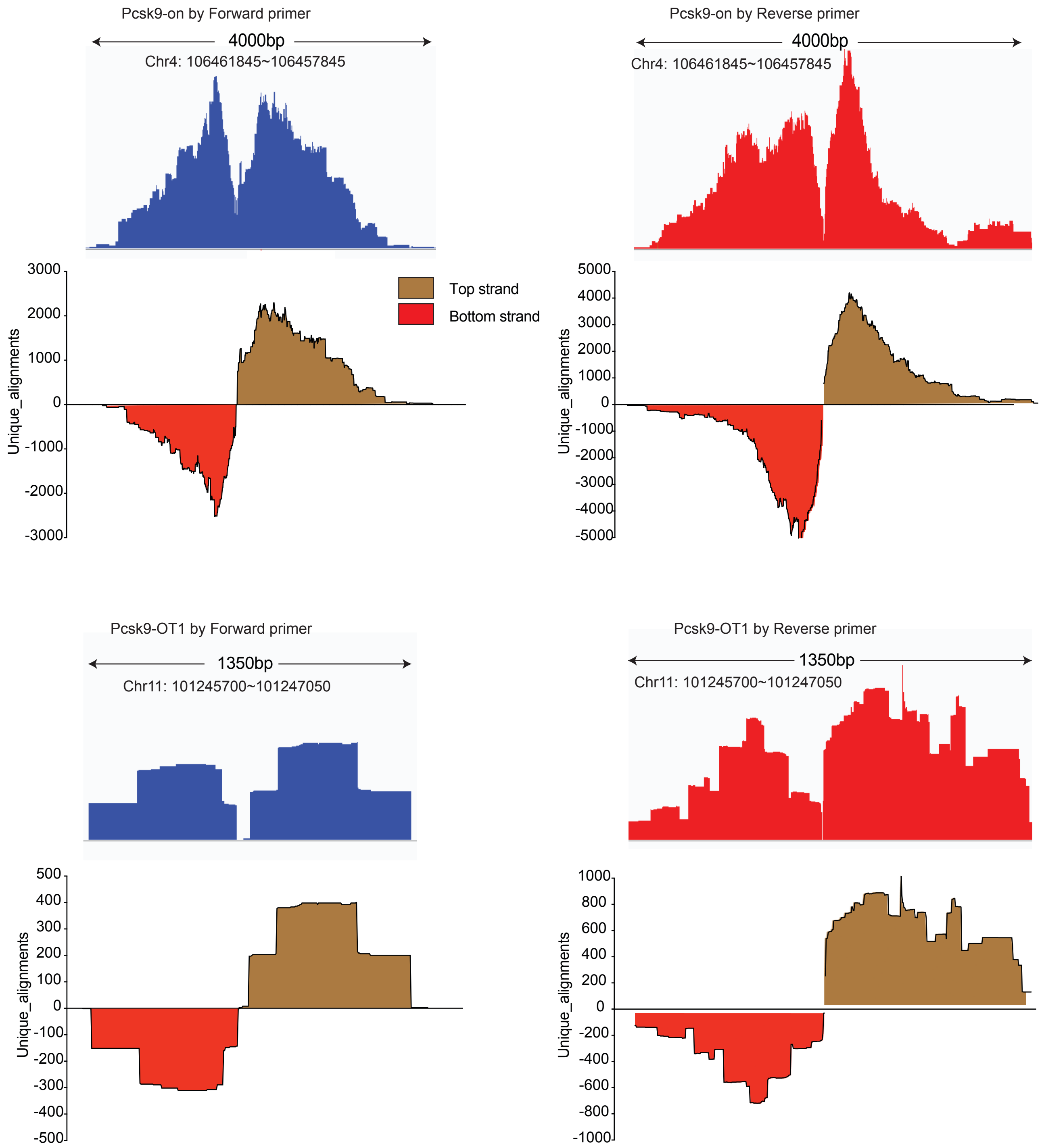

Pcsk9-on by Forward primer
4000bp
Chr4: 106461845~106457845
Unique_alignments
3000
2000
1000
0
-1000
-2000
-3000
Top strand
Bottom strand
Pcsk9-on by Reverse primer
4000bp
Chr4: 106461845~106457845
Unique_alignments
5000
4000
3000
2000
1000
0
-1000
-2000
-3000
-4000
-5000
Pcsk9-OT1 by Forward primer
1350bp
Chr11: 101245700~101247050
Unique_alignments
500
400
300
200
100
0
-100
-200
-300
-400
-500
Pcsk9-OT1 by Reverse primer
1350bp
Chr11: 101245700~101247050
Unique_alignments
1000
800
600
400
200
0
-200
-400
-600
-800
-1000

Supplementary Figure 8. Reads for GUIDE-tag at the Pcsk9 on target-site and off-target site by both primers.

a, Example of read coverage for GUIDE-tag forward (insert primer F) and reverse primers (insert primer R) at the Pcsk9 on-target site (Pcsk9-on) and off-target site 1 (OT1). Top trace in each plot is raw reads mapped to locus. Bottom trace is strand-specific reads.

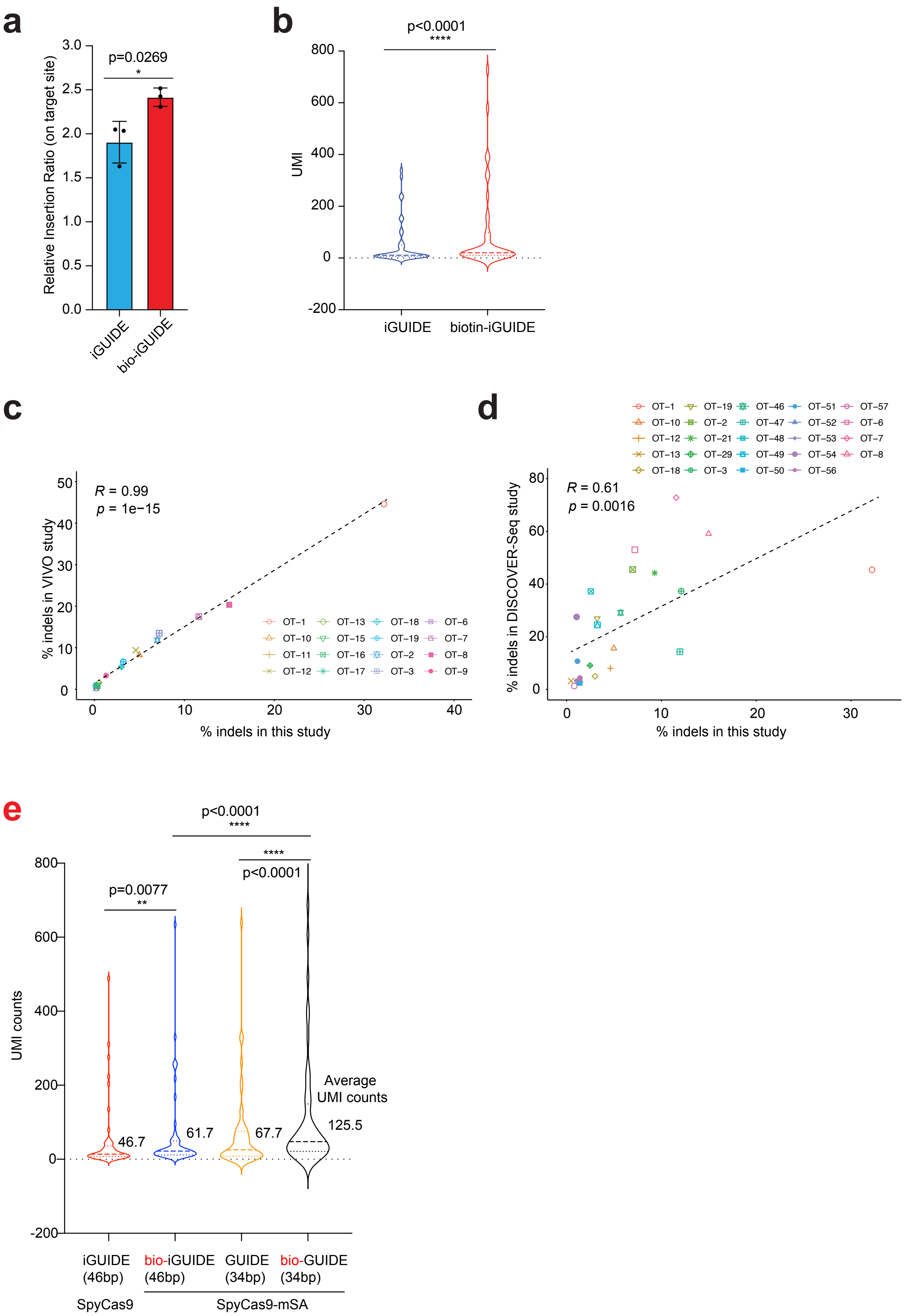
a
Relative Insertion Ratio (on target site)
p=0.0269
*
3.0
2.5
2.0
1.5
1.0
0.5
0.0
iGUIDE
bio-iGUIDE
b
p<0.0001
****
UMI
800
600
400
200
0
-200
iGUIDE
biotin-iGUIDE
c
R = 0.99
p = 1e−15
% indels in VIVO study
% indels in this study
50
40
30
20
10
0
0
10
20
30
40
OT−1
OT−10
OT−11
OT−12
OT−13
OT−15
OT−16
OT−17
OT−18
OT−19
OT−2
OT−3
OT−6
OT−7
OT−8
OT−9
d
R = 0.61
p = 0.0016
% indels in DISCOVER-Seq study
% indels in this study
80
60
40
20
0
0
10
20
30
OT−1
OT−10
OT−12
OT−13
OT−18
OT−19
OT−2
OT−21
OT−29
OT−3
OT−46
OT−47
OT−48
OT−49
OT−50
OT−51
OT−52
OT−53
OT−54
OT−56
OT−57
OT−6
OT−7
OT−8
e
p<0.0001
****
p=0.0077
**
****
p<0.0001
UMI counts
800
600
400
200
0
-200
46.7
61.7
67.7
Average
UMI counts
125.5
iGUIDE
(46bp)
bio-iGUIDE
(46bp)
GUIDE
(34bp)
bio-GUIDE
(34bp)
SpyCas9
SpyCas9-mSA

Supplementary Figure 9. *in vivo* off-target analysis of SpyCas9-mSA with sgPcsk9.

a, Comparison of relative insertion efficiency of the iGUIDE oligo with or without biotin at *Pcsk9* locus by UDiTaS. Relative insertion ratio was normalized to the depth of sequence for each primer and sample. Data are presented as the average of three mice. * $P<0.05$ by unpaired, two-tailed Student's t-test. Error bars are SD.

b, Comparison of the UMIs for iGUIDE insertions recovered at *Pcsk9* and all off-target sites between iGUIDE donor and biotin-iGUIDE donor. The line indicates the median of the data and the box indicates the interquartile range. **** $P<0.0001$ by unpaired, two-tailed Student's t-test.

c-d, Scatter plots of indel frequencies of sgPcsk9 off-target sites identified by this study (GUIDE-tag, x axis) and VIVO [WT mice, day 4 post infection] (c) or DISCOVER-seq (d). Pcsk9 indel frequency was determined by targeted amplicon sequencing (average of three mice). Dashed lines represent the linear regression fit (Pearson correlation calculated). The p-value for Pearson's correlation coefficient was determined by the two-tailed t-distribution table.

e. Comparison of the UMIs of different donor/nuclease at *Pcsk9* off-target sites by GUIDE-tag in mouse liver. Data are presented as the average of three mice. Dashed lines are the average UMI of OT sites. The line indicates the median of the data and the box indicates the interquartile range. **$P<0.01$, ****$P<0.0001$ by two-way ANOVA with Tukey's multiple comparisons test.

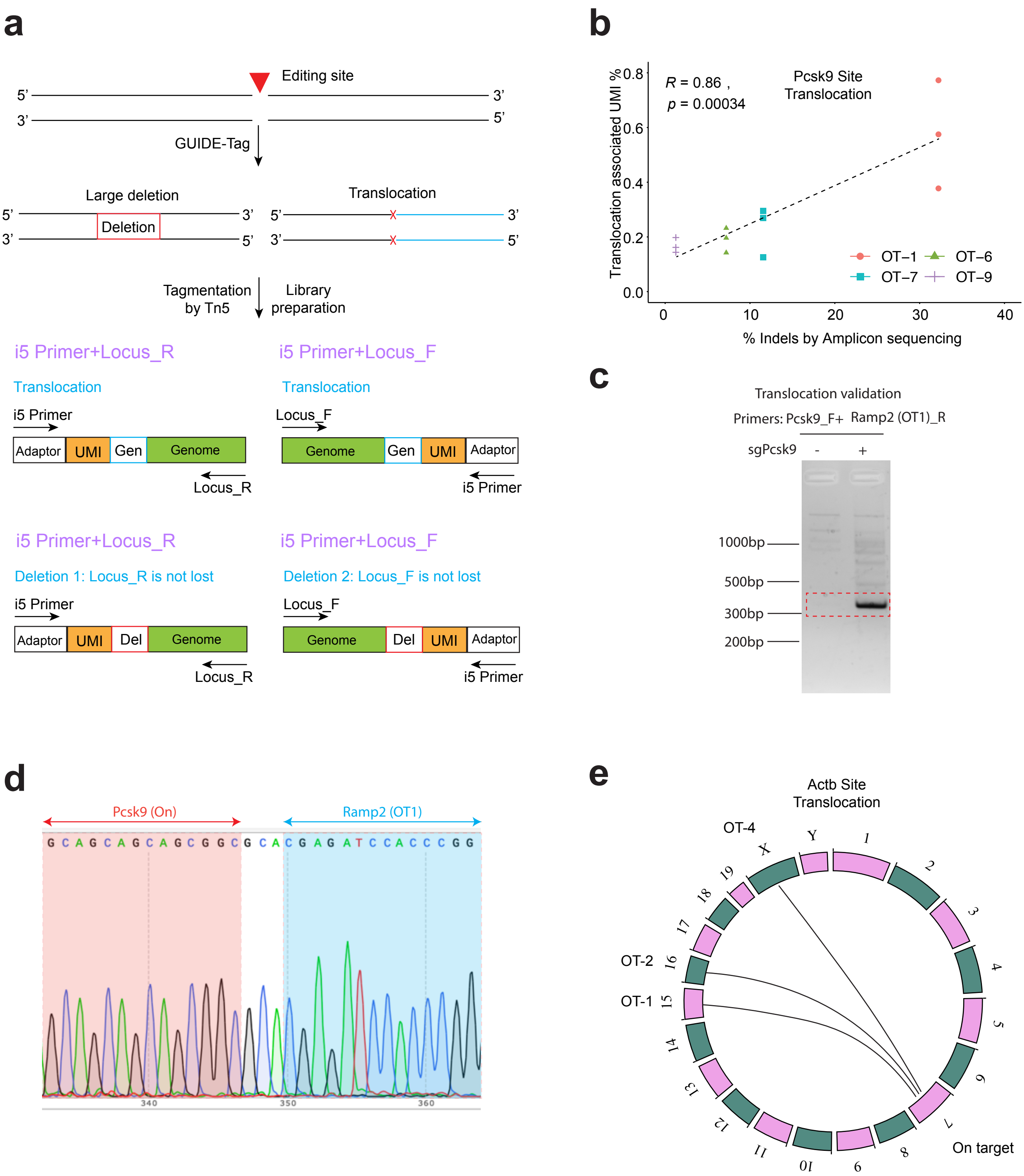
a
Editing site
5'
3'
3'
5'
GUIDE-Tag
Large deletion
Deletion
Translocation
Tagmentation by Tn5
Library preparation
i5 Primer+Locus_R
Translocation
i5 Primer
Adaptor
UMI
Gen
Genome
Locus_R
i5 Primer+Locus_F
Translocation
Locus_F
Genome
Gen
UMI
Adaptor
i5 Primer
i5 Primer+Locus_R
Deletion 1: Locus_R is not lost
i5 Primer
Adaptor
UMI
Del
Genome
Locus_R
i5 Primer+Locus_F
Deletion 2: Locus_F is not lost
Locus_F
Genome
Del
UMI
Adaptor
i5 Primer
b
R = 0.86 ,
p = 0.00034
Pcsk9 Site
Translocation
Translocation associated UMI %
0.0
0.2
0.4
0.6
0.8
0
10
20
30
40
% Indels by Amplicon sequencing
OT−1
OT−6
OT−7
OT−9
c
Translocation validation
Primers: Pcsk9_F+ Ramp2 (OT1)_R
sgPcsk9
-
+
1000bp
500bp
300bp
200bp
d
Pcsk9 (On)
Ramp2 (OT1)
340
350
360
e
Actb Site
Translocation
OT-4
OT-2
OT-1
On target
X
Y
1
2
3
4
5
6
7
8
9
10
11
12
13
14
15
16
17
18
19

Supplementary Figure 10. Detection of translocations and large deletions by UDiTaS.

a, overview of deep sequencing library preparation for detection of translocations and large deletions by UDiTaS. Gen, - alternate genomic locus from translocation fusion.

b, Scatter plots of UMI% for each statistically significant translocation observed by UDiTaS and indel frequency (average of three mice; indel% by amplicon sequencing at sgPcsk9 OT sites associated with each translocation). Dashed lines represent the linear regression fit (Pearson correlation calculated). The p-value for Pearson's correlation coefficient was determined by the two-tailed t-distribution table.

c-d, Translocation between Pcsk9 and Ramp2 (OT-1) was validated by junction primer PCR (c) and sanger sequencing (d).

e, Circos plot of statistically significant translocations identified by UDiTaS for SpyCas9-mSA* and sg*Actb* in mouse liver. Identified translocations are indicated as arcs between two chromsomes.

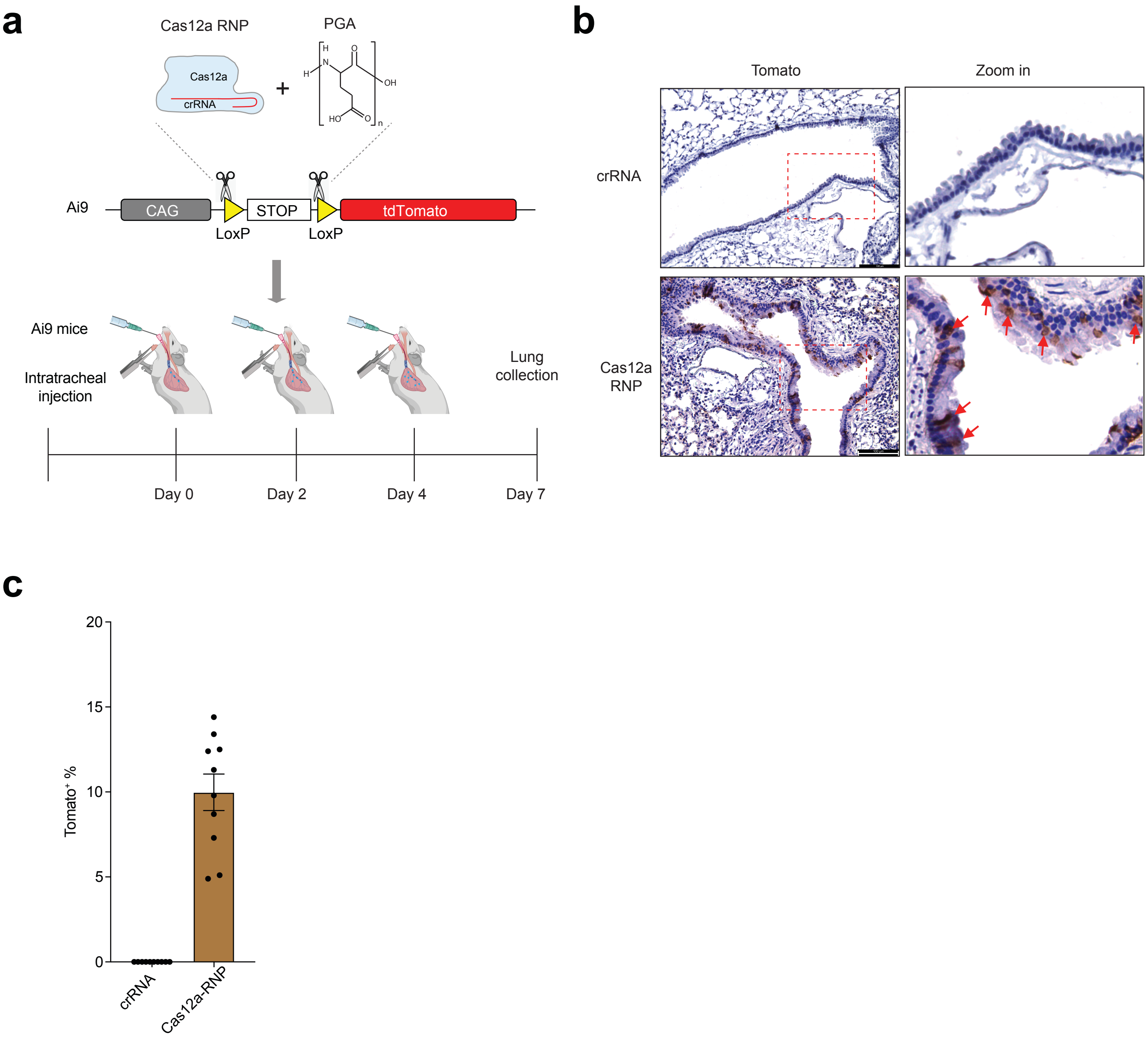
a
Cas12a RNP
PGA
Cas12a
crRNA
+
Ai9
CAG
STOP
tdTomato
LoxP
LoxP
Ai9 mice
Intratracheal injection
Lung collection
Day 0
Day 2
Day 4
Day 7
b
Tomato
Zoom in
crRNA
Cas12a RNP
c
Tomato⁺ %
20
15
10
5
0
crRNA
Cas12a-RNP

Supplementary Figure 11. Genome editing in mouse lung by intratracheal delivery of Cas12a RNP.

a, Deliver PGA with enAsCas12a protein and LoxP crRNA intratracheally in LSL-Tomato reporter mice. **b**, Representative Tomato IHC staining (n=3 mice). crRNA only serves as a negative control. Arrows denote Tomato$^+$ cells. **c**, Quantification of Tomato$^+$ cells in large airways. Each dot is the ratio of Tomato$^+$ / total cells in an airway. Results are mean ± SEM (n=10 airways in 3 mice).

a

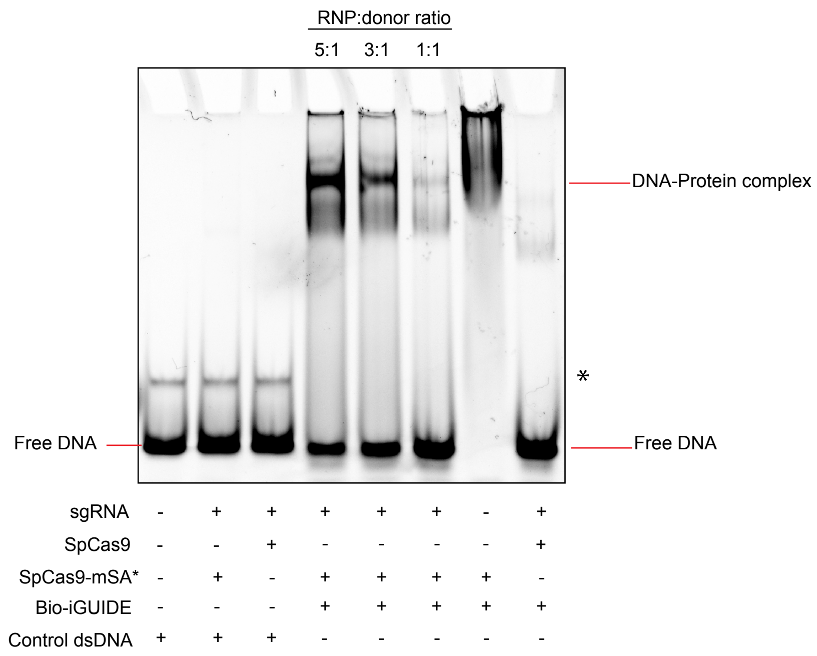

RNP:donor ratio
5:1
3:1
1:1
DNA-Protein complex
*
Free DNA
Free DNA
sgRNA - + + + + + - +
SpCas9 - - + - - - - +
SpCas9-mSA* - + - + + + + -
Bio-iGUIDE - - - + + + + +
Control dsDNA + + + - - - - -

b

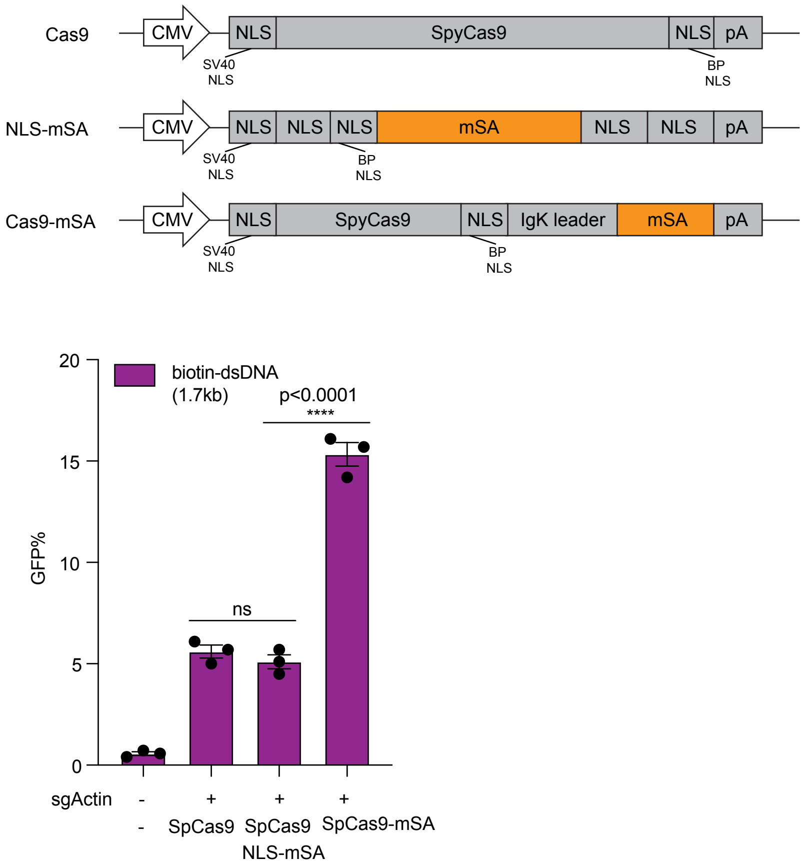

Cas9
CMV
NLS
SpyCas9
NLS
pA
SV40 NLS
BP NLS
NLS-mSA
CMV
NLS
NLS
NLS
mSA
NLS
NLS
pA
SV40 NLS
BP NLS
Cas9-mSA
CMV
NLS
SpyCas9
NLS
IgK leader
mSA
pA
SV40 NLS
BP NLS
biotin-dsDNA (1.7kb)
p<0.0001
****
ns
GFP%
20
15
10
5
0
sgActin - + + +
-
SpCas9
SpCas9 NLS-mSA
SpCas9-mSA

c

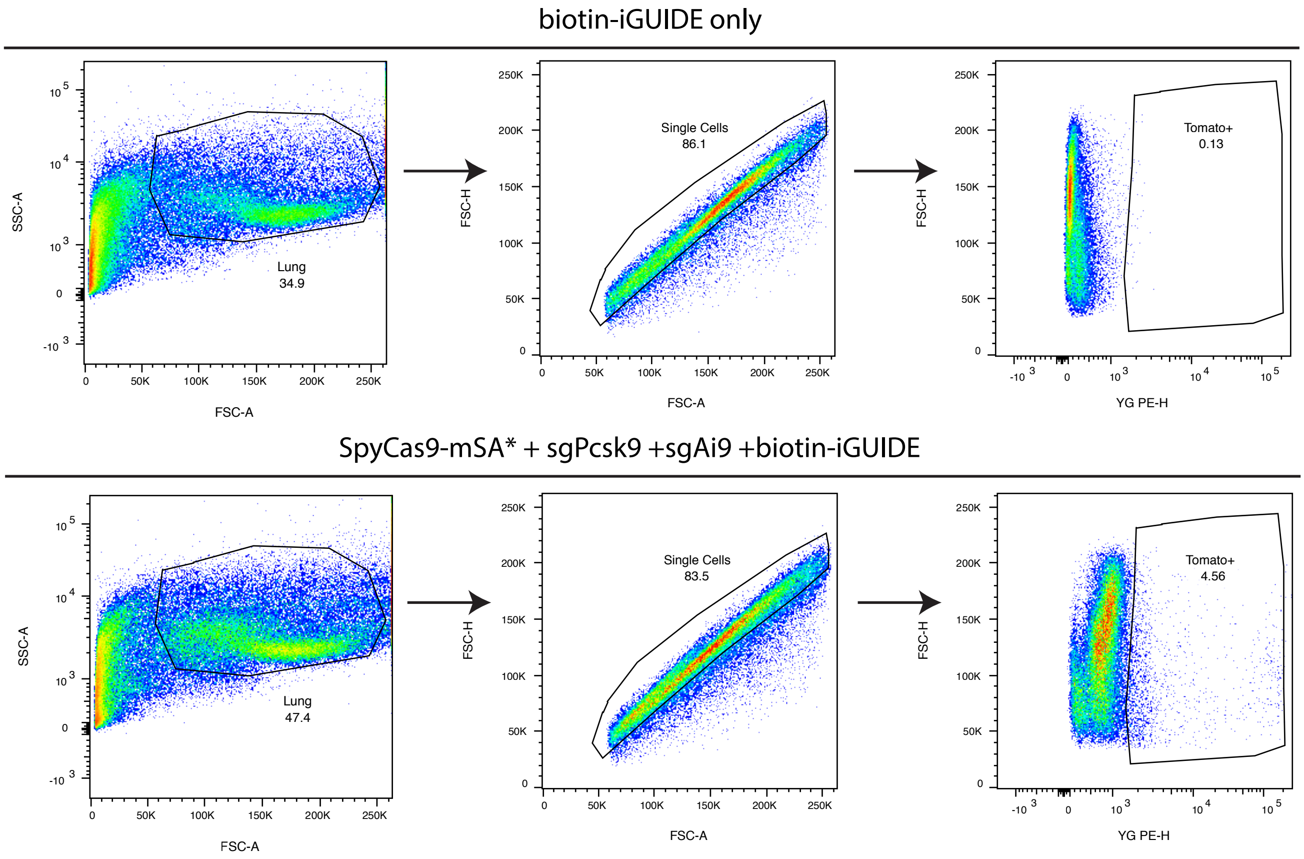

biotin-iGUIDE only
SSC-A
FSC-A
Lung
34.9
FSC-H
Single Cells
86.1
YG PE-H
Tomato+
0.13
SpyCas9-mSA* + sgPcsk9 +sgAi9 +biotin-iGUIDE
Lung
47.4
Single Cells
83.5
Tomato+
4.56

Supplementary Figure 12. Cas9-mSA* binds to biotin-dsDNA.

a, Electrophoretic mobility shift assays (EMSA) analysis of SpyCas9-mSA* protein binding to biotin-dsDNA donor. 10 pmol SpyCas9-mSA* (or SpyCas9 lacking mSA) and sgRNA were mixed at an equal molor ratio and incubated at room temperature for 10mins, then the Cas9-mSA* RNP was incubated with biotin-iGUIDE-Cy3 donor or a control Cy3-labeled DNA lacking biotin at a 5:1 molor ratio unless otherwise indicated in a total volume of 30µL EMSA buffer. Samples were analyzed by native PAGE to visualize Cy3 fluorescence. Cas9-mSA*+donor without sgRNA (lane 7) did not migrate efficiently into the gel. * denotes non-specific bands.

b, N2A cells were transfected with biotin-IRES-GFP donor with various nucleases (SpyCas9/sgRNA, SpyCas9/NLS-mSA/sgRNA or SpyCas9-mSA/sgRNA expression vector). Flow cytometry analysis was performed 4 days after transfection. Biotin-dsDNA with SpyCas9-mSA fusion protein increases %GFP+ cells compared to the unfused SpyCas9 & NLS-mSA constructs. Results were obtained from three independent experiments and presented as mean ± SD. ****P<0.0001 by one-way ANOVA with Tukey's multiple comparisons test. ns, not significant.

c, Ai9 reporter mice were dosed with SpyCas9-mSA*+sgPcsk9 RNP, SpyCas9-mSA*+ Ai9 sgRNA RNP and bio-iGUIDE donor intratracheally three times over a four day span. 7days later, lungs were dissected into single cells and Tomato positive cells were collected by FACS.

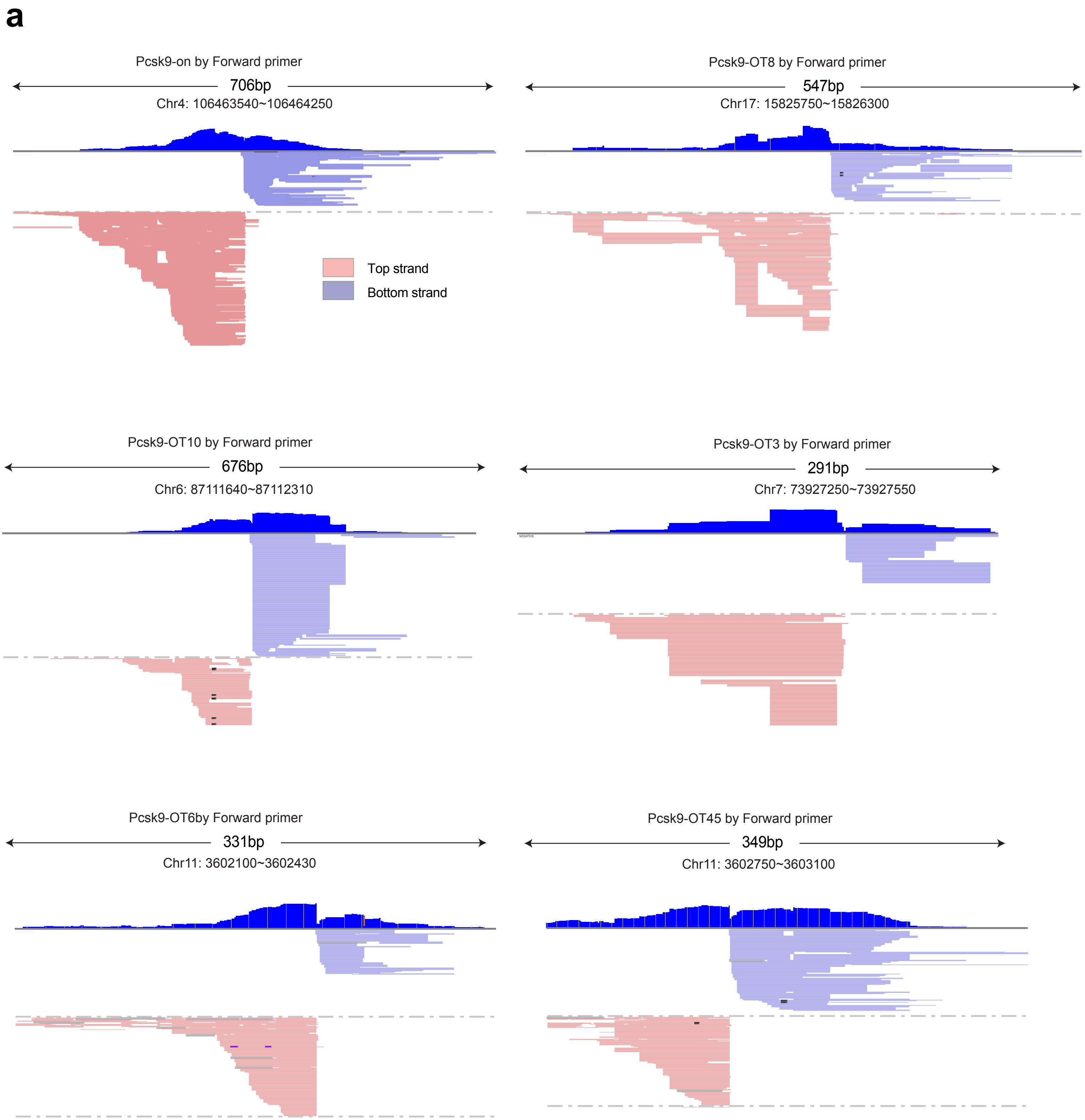
a
Pcsk9-on by Forward primer
706bp
Chr4: 106463540~106464250
Top strand
Bottom strand
Pcsk9-OT8 by Forward primer
547bp
Chr17: 15825750~15826300
Pcsk9-OT10 by Forward primer
676bp
Chr6: 87111640~87112310
Pcsk9-OT3 by Forward primer
291bp
Chr7: 73927250~73927550
Pcsk9-OT6by Forward primer
331bp
Chr11: 3602100~3602430
Pcsk9-OT45 by Forward primer
349bp
Chr11: 3602750~3603100

Supplementary Figure 13. GUIDE-tag reads from the lung for sg*Pcsk9*. Example of read coverage for GUIDE-tag forward primer (insert primer F) at the Pcsk9 on-target site (Pcsk9-on) and off-target sites. Top trace in each plot is raw reads mapped to the locus. Bottom trace is strand-specific reads.

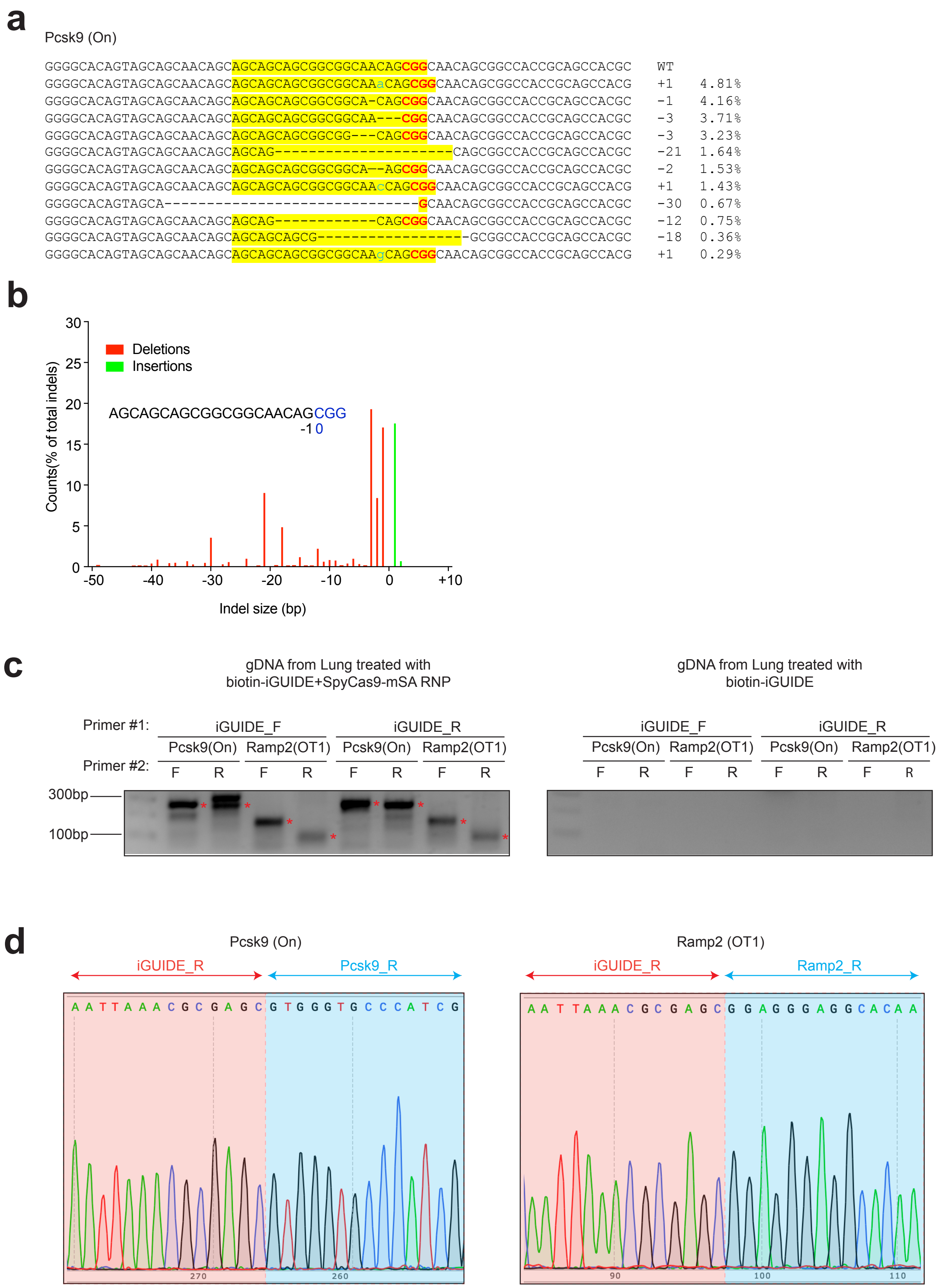
a
Pcsk9 (On)
GGGGCACAGTAGCAGCAACAGCAGCAGCAGCGGCGGCAACAGCGGCAACAGCGGCCACCGCAGCCACGC WT
GGGGCACAGTAGCAGCAACAGCAGCAGCAGCGGCGGCAAaCAGCGGCAACAGCGGCCACCGCAGCCACG +1 4.81%
GGGGCACAGTAGCAGCAACAGCAGCAGCAGCGGCGGCA-CAGCGGCAACAGCGGCCACCGCAGCCACGC -1 4.16%
GGGGCACAGTAGCAGCAACAGCAGCAGCAGCGGCGGCAA---CGGCAACAGCGGCCACCGCAGCCACGC -3 3.71%
GGGGCACAGTAGCAGCAACAGCAGCAGCAGCGGCGG---CAGCGGCAACAGCGGCCACCGCAGCCACGC -3 3.23%
GGGGCACAGTAGCAGCAACAGCAGCAG---------------------CAGCGGCCACCGCAGCCACGC -21 1.64%
GGGGCACAGTAGCAGCAACAGCAGCAGCAGCGGCGGCA--AGCGGCAACAGCGGCCACCGCAGCCACGC -2 1.53%
GGGGCACAGTAGCAGCAACAGCAGCAGCAGCGGCGGCAAcCAGCGGCAACAGCGGCCACCGCAGCCACG +1 1.43%
GGGGCACAGTAGCA------------------------------GCAACAGCGGCCACCGCAGCCACGC -30 0.67%
GGGGCACAGTAGCAGCAACAGCAGCAG------------CAGCGGCAACAGCGGCCACCGCAGCCACGC -12 0.75%
GGGGCACAGTAGCAGCAACAGCAGCAGCAGCG------------------GCGGCCACCGCAGCCACGC -18 0.36%
GGGGCACAGTAGCAGCAACAGCAGCAGCAGCGGCGGCAAgCAGCGGCAACAGCGGCCACCGCAGCCACG +1 0.29%
b
Deletions
Insertions
AGCAGCAGCGGCGGCAACAGCGG
-1 0
Counts(% of total indels)
Indel size (bp)
c
gDNA from Lung treated with biotin-iGUIDE+SpyCas9-mSA RNP
gDNA from Lung treated with biotin-iGUIDE
Primer #1:
iGUIDE_F
iGUIDE_R
Primer #2:
Pcsk9(On)
Ramp2(OT1)
F
R
300bp
100bp
d
Pcsk9 (On)
Ramp2 (OT1)
iGUIDE_R
Pcsk9_R
Ramp2_R
AATTAAACGCGAGCGTGGGTGCCCATCG
AATTAAACGCGAGCGGAGGGAGGCACAA

Supplementary Figure 14. *in vivo* off-target analysis of sgPcsk9 in mouse lung.

a, The percentage of most common sequences at Pcsk9 site in sorted lung cells determined by amplicon sequencing (representative lung of n=2). sgPcsk9 target site is in yellow. PAM sequences are in red. Deleted bases are indicated by dashes.

b, Indel sizes induced by SpyCas9-mSA complex at Pcsk9 site in sorted lung cells.

c, Validation of biotin-iGUIDE insertion at Pcsk9 site (on) and Ramp2 (OT1) in mouse lung. biotin-iGUIDE only mice serve as a negative control. PCR was performed with indicated primers in to amplify the 5' junction sites and 3' junction site for insertions in different orientations. * denotes expected PCR bands. Results were obtained from four independent experiments.

d, Validation of biotin-iGUIDE insertion at Pcsk9 site (on) and Ramp2 (OT1) in mouse lung by TOPO cloning and Sanger sequencing.

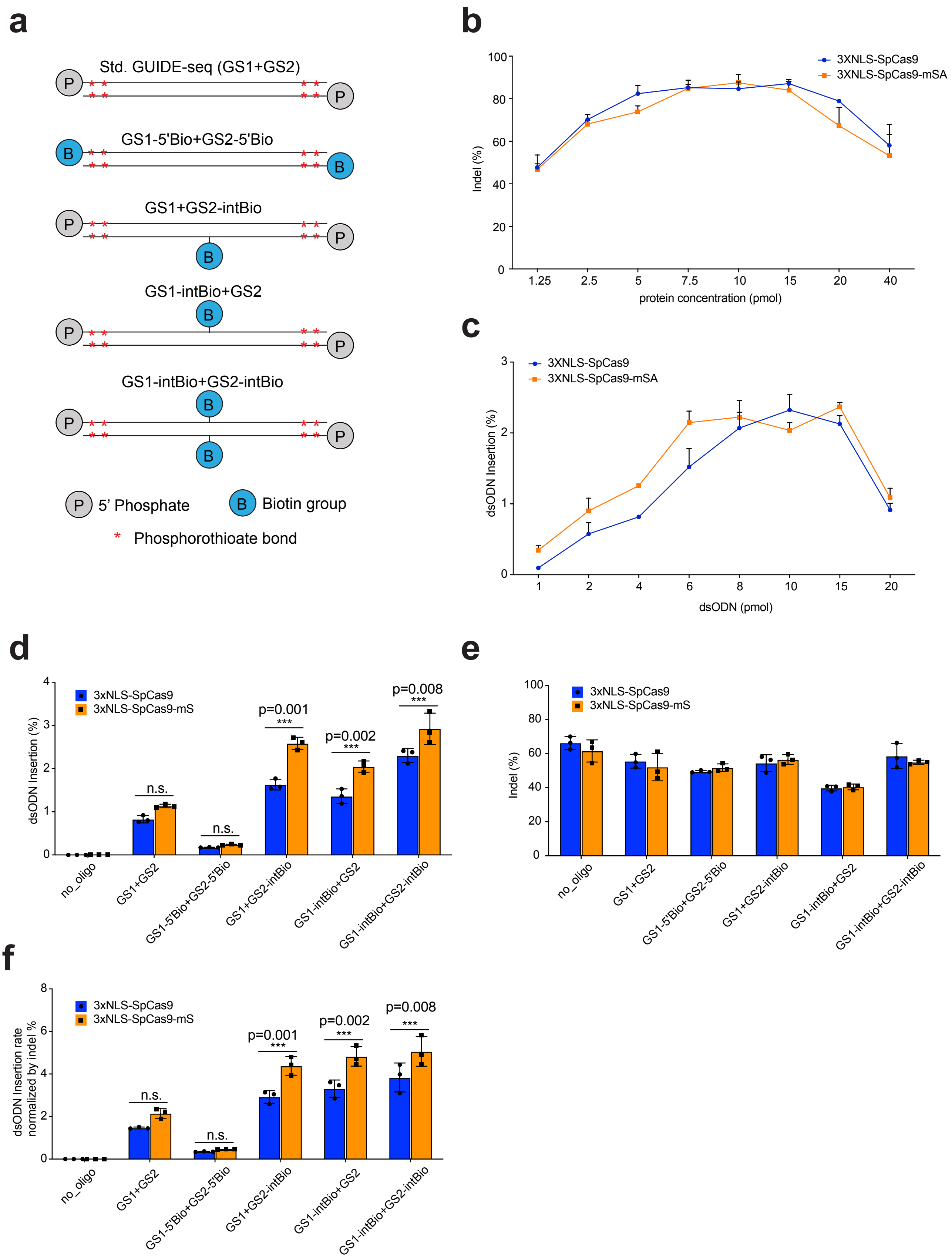
a
Std. GUIDE-seq (GS1+GS2)
GS1-5'Bio+GS2-5'Bio
GS1+GS2-intBio
GS1-intBio+GS2
GS1-intBio+GS2-intBio
P 5' Phosphate
B Biotin group
* Phosphorothioate bond
b
3XNLS-SpCas9
3XNLS-SpCas9-mSA
Indel (%)
protein concentration (pmol)
c
3XNLS-SpCas9
3XNLS-SpCas9-mSA
dsODN Insertion (%)
dsODN (pmol)
d
3xNLS-SpCas9
3xNLS-SpCas9-mS
dsODN Insertion (%)
n.s.
n.s.
p=0.001
p=0.002
p=0.008
no_oligo
GS1+GS2
GS1-5'Bio+GS2-5'Bio
GS1+GS2-intBio
GS1-intBio+GS2
GS1-intBio+GS2-intBio
e
3xNLS-SpCas9
3xNLS-SpCas9-mS
Indel (%)
f
3xNLS-SpCas9
3xNLS-SpCas9-mS
dsODN Insertion rate normalized by indel %
n.s.
n.s.
p=0.001
p=0.002
p=0.008

Supplementary Figure 15. Optimization of GUIDE-seq donor improves tag insertion efficiency in cell culture

a, Schematic representation of different GUIDE-seq donor duplexes. Duplex oligonucleotides were prepared by annealing two chemically synthesized oligonucleotides with biotin tethered at different positions (5' Biotin [5'Bio] or Internal Biotin [intBio; Biotin dT]) within the GUIDE-seq donor sequence.

b, Hepa1-6 cells were electroporated with the indicated concentration of 3xNLS-SpyCas9 sgPcsk9 RNP or 3xNLS-SpyCas9-mSA sgPcsk9 RNP without GUIDE-seq donor. gDNA were isolated 3 days after electroporation from each group and indel percentages were measured by deep sequencing amplicons spanning the Pcsk9 target site. Indel% are presented as mean ± s.d. (n=3).

c, Hepa1-6 cells were electroporated with 2 pmol of 3xNLS-SpyCas9 sgPcsk9 RNP or 3xNLS-SpyCas9-mSA sgPcsk9 RNP and the indicated dose of GUIDE-seq donor (dsODN) containing a single internal biotin (GS1+GS2-intBio). gDNA were isolated 3 days after electroporation from each group and the insertion percentages were measured by deep sequencing of PCR amplicons spanning the Pcsk9 target site. Insertion% are presented as mean ± s.d. (n=3).

d-e, Hepa1-6 cells were electroporated with 2 pmol of 3xNLS-SpyCas9 sgPcsk9 RNP or 3xNLS-SpyCas9-mSA sgPcsk9 RNP and 5pmol of each different GUIDE-seq donor. gDNA were isolated 3 days after electroporation from each group and the insertion (**d**) and indel (**e**) percentages were measured by deep sequencing of PCR amplicons spanning the Pcsk9 target site. Insertion% and indel% are presented as mean ± s.d. (n=3). n.s., not significant. ****P*<0.001 by two-way ANOVA with Tukey's multiple comparisons test.

f, Insertion% from (**d**) was normalized with indel% for each group. Insertion% are presented as mean ± s.d. (n=3). n.s., not significant. ****P*<0.001 by two-way ANOVA with Tukey's multiple comparisons test.

Supplementary Figure 1C

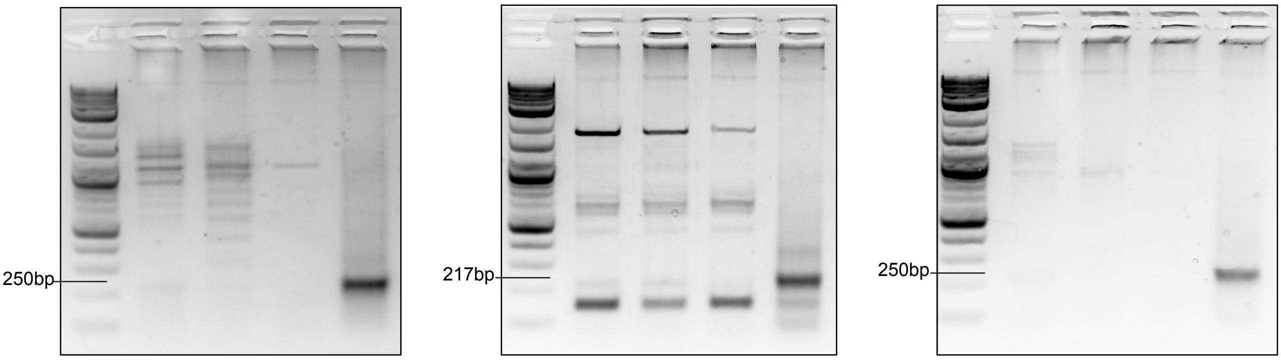

Supplementary Figure 6C

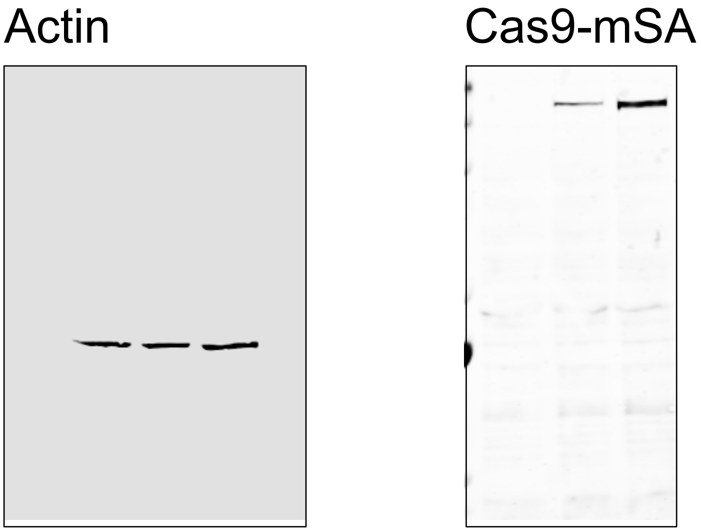

Supplementary Figure 14

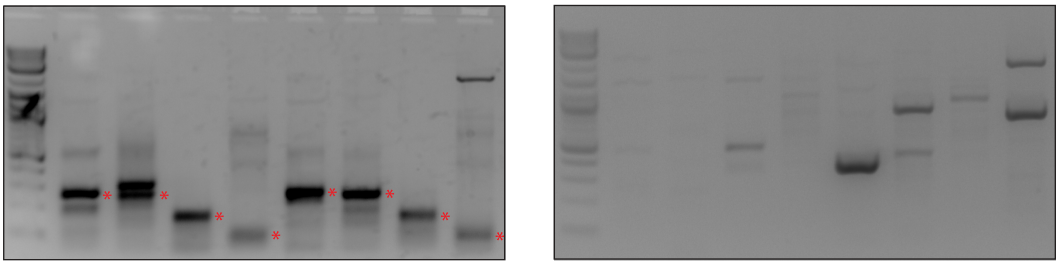

Supplementary Figure 16. Uncropped gel images.

Supplementary Table 1. Sequences of sgRNAs used in this study. All sequences are shown in 5' to 3' orientation.

Target sites	sequence (5'-3')	Cas9	
Actin.3'UTR	CCACCCCCACTCCTAAGAGG	SpyCas9	in vivo
Fah intron 1.2	AATGACTTAATACAATTGCT	SpyCas9	
Fah intron 1.3	GGCCTTGTTCACATATAACT	SpyCas9	in vivo
Fah intron 1.4	TACCCATTAATTTCCTCCCT	SpyCas9	
Fah intron 1.5	CAGTAGGAGGTTCTCCTCTT	SpyCas9	
PCSK9	AGCAGCAGCGGCGGCAACAG	SpyCas9	in vivo
GAPDH	AGCCCCAGCAAGAGCACAAG	SpyCas9	In vitro
sgRNA-tdTom	AAGTAAAACCTCTACAAATG	SpyCas9	In vivo
Actin.3'UTR	CAGGAAGTCCCTCACCCTCCCAA	enAsCas12a	In vitro
LoxP	GTATAATGTATGCTATACGAAGT	enAsCas12a	In vivo
Actin.3'UTR	CGACCATCCTCCTCTTAGGAGT	SauCas9	In vitro

Supplementary Table 2. PCR donors used in this study.

Figure	Donor size	Name	Primer
Figure 1b	1.7Kb	IRES-GFP	1640 and 1764
	1.7Kb	biotin-IRES-GFP	4035 and 3586
Figure 1c	1.7Kb	biotin-IRES-GFP	4035 and 3586
	2.5Kb	biotin-IRES-GFP	4035 and 4036
	3.5Kb	biotin-IRES-GFP	4035 and 4037
	4.5Kb	biotin-IRES-GFP	4035 and 4038
Figure 1d	1.7Kb	IRES-GFP	1640 and 1764
	1.7Kb	biotin-IRES-GFP	4035 and 3586
Figure 1e	1.4Kb	Fah Exon 2-14	3891 and 3892
	1.4Kb	biotin-Fah Exon 2-15	3921 and 3921
Figure 3	42bp	iGUIDE	4412 and 4413
	42bp	biotin-iGUIDE	4404 and 4405
Figure 5a	1.7Kb	IRES-GFP	1640 and 1764
	1.7Kb	biotin-IRES-GFP	4035 and 3586
Figure 5b	42bp	biotin-iGUIDE	4404 and 4405

Supplementary Table 3. Sequences of primers used for DNA amplification and high throughput sequencing. All sequences are shown in 5' to 3' orientation.

Primers used for amplification:

Primer	Sequence	Modification
1640_IRESdonor1700_F	CGCCAGGGTTTTCCCAGTCACGAC	No
1764_IRESdonor1700_R	AGCGGATAACAATTTCACACAGG	No
4035_IRESdonor_F	GCCGCTCTAGAACTAGTGGAT	5'biotin
3586_IRESdonor1700_R	AGCGGATAACAATTTCACACAGG	5'biotin
4036_IRESdonor2500_R	CCAGTGGCGATAAGTCGTGT	5'biotin
4037_IRESdonor3500_R	ACGGATGGCATGACAGTAAGA	5'biotin
4038_IRESdonor4500_R	AAGAGGCCCGCACCGAT	5'biotin

3835 Actin 5junction_F	AATAGTCATTCCAAGTATCC	No
4151 Actin 3junction_F	GCGGTGGGCTCTATGGATAA	No
3837 Actin 5junction_R	GAGGAGCACGCGTCAATTGC	No
3838 Actin 3junction_R	CACAGAAGCAATGCTGTCAC	No
3865_pho_F	CGCCAGGGTTTTCCCAGTCACGAC	5‘phosphorylation
3866_pho_R	AGCGGATAACAATTTCACACAGG	5‘phosphorylation
3891 Fah_F	GGTTGTCTCAAAGGAAACCATGA	No
3892 Fah_R	CTCCCCCTGAACCTGAAACAT	No
3920 bio_Fah_F	GGTTGTCTCAAAGGAAACCATGA	5'biotin
3921 bio_Fah_R	CTCCCCCTGAACCTGAAACAT	5'biotin
3986_FAH_1F	TATTCCAGTGGCCGAGGACT	No
3987_FAH_6R	ACCACAATGGAGGAAGCTCG	No
3859 Fah intron PCR F	CTAGGTACATTTTCCTGGAC	No
3860 Fah intron PCR R	CTGCTTCAAAGATCAGAAAGG	No
3849 Fah 5'junction F	TACCCATTAATTTCCTCCCT	No
4153 Fah 5'junction R	GATGTTTGGAAAGGGCAGGT	No
OT1-5p	TGGTAGAACACCTGCCTGGAATC	No
OT2-5p	AGTAGCAAGATGGGTGTGACTGGAG	No
OT3-5p	TGCCCTACAAACTTGCCTACTATCC	No
OT5-5p	CATTCATCTTGGACAACTCTGGCTT	No
OT7-5p	GGAGGGAGAAAAAGAAGAAGCACA	No
OT4-5p	AAAGGCTCTGGGAAATGCCACTC	No
OT6-5p	CAACAGTTTTGTATCAGAGTAAGCCCAT	No
OT8-5p	GTGGAGCCTTCGTGATGTTGTCT	No
OT9-5p	CCCAGTTATTAGTGTGAGAAGCAAGGT	No
OT10-5p	CTACCTTTATGCTTTTGCGGGG	No
4404_iGUIDE_F	bio/G*C*TCGCGTTTAATTGAGTTGTCATATGTTAATAACGGTATACGC*G*A	5'biotin
4405_iGUIDE_R	bio/T*C*GCGTATACCGTTATTAACATATGACAACTCAATTAAACGCGA*G*C	5'biotin
4412_iGUIDE_F	G*C*TCGCGTTTAATTGAGTTGTCATATGTTAATAACGGTATACGC*G*A	phosphorylation
4413_iGUIDE_R	T*C*GCGTATACCGTTATTAACATATGACAACTCAATTAAACGCGA*G*C	phosphorylation
4426_GUIDE_F	bio/G*T*TTAATTGAGTTGTCATATGTTAATAACGGT*A*T	5'biotin
4427_GUIDE_R	bio/A*T*ACCGTTATTAACATATGACAACTCAATTAA*A*C	5'biotin
4428_GUIDE_F	G*T*TTAATTGAGTTGTCATATGTTAATAACGGT*A*T	phosphorylation
4429_GUIDE_R	A*T*ACCGTTATTAACATATGACAACTCAATTAA*A*C	phosphorylation

*=phosphorothioate; bio/ = biotin

Primers used for high throughput sequencing

Fah_Locus_F	GTGACTGGAGTTCAGACGTGTGCTCTTCCGATCTAGCTGGGTAAGACTACCCATTAATTTCCTCCCT
Fah_Locus_R	GTGACTGGAGTTCAGACGTGTGCTCTTCCGATCTTCAGGAAAGACCCAGAGGCAATC
Fah_Insert_R	GTGACTGGAGTTCAGACGTGTGCTCTTCCGATCTGAAGACATGTTGATGTTTGGAAAGGGCAGGT
Fah_Insert_F	GTGACTGGAGTTCAGACGTGTGCTCTTCCGATCTCATTCATTTTATGTTTCAGGTTCAGGG
i5 Primer	AATGATACGGCGACCACCGAGATCTACAC
4291_Fah_OT1_F	CTACACGACGCTCTTCCGATCTTGGTAGAACACCTGCCTGGAATC
4292_Fah_OT1_R	AGACGTGTGCTCTTCCGATCTCACAACCAAAAGGCTCAGGCTAC
4293_Fah_OT2_F	CTACACGACGCTCTTCCGATCTAGTAGCAAGATGGGTGTGACTGGAG
4294_Fah_OT2_R	AGACGTGTGCTCTTCCGATCTTCCTGCCCACACACTTGCTTC

4295_Fah_OT3_F	CTACACGACGCTCTTCCGATCTTGCCCTACAAACTTGCCTACTATCC
4296_Fah_OT3_R	AGACGTGTGCTCTTCCGATCTCTTCTTGTTCTTGTCCATTGCCTTT
4297_Fah_OT4_F	CTACACGACGCTCTTCCGATCTAACTCTGGCTTGTGTCAAGTTAA
4298_Fah_OT4_R	AGACGTGTGCTCTTCCGATCTCTGTTTCAGAATATAGGGAGCC
4299_Fah_OT5_F	CTACACGACGCTCTTCCGATCTTCTGGGAAATGCCACTCGCTTG
4300_Fah_OT5_R	AGACGTGTGCTCTTCCGATCTTGCCCAGGTCTCATTCCTTCTCC
4301_Fah_OT6_F	CTACACGACGCTCTTCCGATCTGAAGTAAAAAGGGGGAGGGAGAA
4302_Fah_OT6_R	AGACGTGTGCTCTTCCGATCTAACACATTGGAATTGGACAAAACA
4307_Fah_OT7_F	CTACACGACGCTCTTCCGATCTATTTTTATTGACCCAGTTATTAGTGTGA
4308_Fah_OT7_R	AGACGTGTGCTCTTCCGATCTAATGAATATAACCAACAAAATGAGTACAC
4309_Fah_OT8_F	CTACACGACGCTCTTCCGATCTCCAGGTAGGAATAAGTCAGCCGA
4310_Fah_OT8_R	AGACGTGTGCTCTTCCGATCTTAGAGTTGTAAAACGGAAACCCTTG
4311_Fah_OT9_F	CTACACGACGCTCTTCCGATCTTGAAGGGGACTGGGGAGATG
4312_Fah_OT9_R	AGACGTGTGCTCTTCCGATCTATTTTTTGTCTCCCTTGCTTTAGTATTA
4530_Actb_Locus_F	GTGACTGGAGTTCAGACGTGTGCTCTTCCGATCTATCCATGAAATAAGTGGTTACAGGAAGTC
4531_Actb_insert_R	GTGACTGGAGTTCAGACGTGTGCTCTTCCGATCTGGAGAGGAGCACGCGTCAATTGC
4532_Actb_insert_F	GTGACTGGAGTTCAGACGTGTGCTCTTCCGATCTGGGGATGCGGTGGCTCTATGGATAA
4533_Actb_Locus_R	GTGACTGGAGTTCAGACGTGTGCTCTTCCGATCTTTACACAGAAGCAATGCTGTCACCTTCCC
4534_Pcsk9_Locus_F	GTGACTGGAGTTCAGACGTGTGCTCTTCCGATCTGGACGGGAGGGCGAGCATCAG
4535_Pcsk9_Locus_R	GTGACTGGAGTTCAGACGTGTGCTCTTCCGATCTAGGCGTCCATGTCCTTCCCGA
iGUIDE_insert_F	GTGACTGGAGTTCAGACGTGTGCTCTTCCGATCTTTAATTGAGTTGTCATATGTTAATAACGGT
iGUIDE_insert_R	GTGACTGGAGTTCAGACGTGTGCTCTTCCGATCTCCGTTATTAACATATGACAACTCAATTAA
4547_Pcsk9_On_F	CTACACGACGCTCTTCCGATCTTCATCAGCCAGGCCATCCTCCT
4573_Pcsk9_On_R	AGACGTGTGCTCTTCCGATCTGTCCCAGGCGTCCATGTCCTTC
4546_Pcsk9_OT1_F	CTACACGACGCTCTTCCGATCTGGATCTCGGCTTGGTGTGACCC
4572_Pcsk9_OT1_R	AGACGTGTGCTCTTCCGATCTGCTCCGCACGTCGATTGGTACT
4551_Pcsk9_OT2_F	CTACACGACGCTCTTCCGATCTACAACAAAAACCCACCGCAGCG
4577_Pcsk9_OT2_R	AGACGTGTGCTCTTCCGATCTGCGACTCCTCTGCACCTCCTC
4706_Pcsk9_OT3_F	CTACACGACGCTCTTCCGATCTGTTAGAAGGGAAAAAGGGTGTAGACT
4707_Pcsk9_OT3_R	AGACGTGTGCTCTTCCGATCTCAGCCACTAACTGTGATTGTCTCC
4559_Pcsk9_OT4_F	CTACACGACGCTCTTCCGATCTAGGCTACTCACCTCCCCTGCTG
4585_Pcsk9_OT4_R	AGACGTGTGCTCTTCCGATCTCGTCTGCCTGTGGGTCTGTTCA
4565_Pcsk9_OT5_F	CTACACGACGCTCTTCCGATCTCCAGGCCCTCGCATCACTGTAA
4591_Pcsk9_OT5_R	AGACGTGTGCTCTTCCGATCTAGGACACTGGTGGGAGTCTTAGGA
4550_Pcsk9_OT6_F	CTACACGACGCTCTTCCGATCTTCCAACAAACCAGCAAACCAGCA
4576_Pcsk9_OT6_R	AGACGTGTGCTCTTCCGATCTGGAGAGGTGTAATTTTTGCCAGCTGC
4549_Pcsk9_OT7_F	CTACACGACGCTCTTCCGATCTCGGTGGCTTCTCCCTTCGCTG
4575_Pcsk9_OT7_R	AGACGTGTGCTCTTCCGATCTTCTCTTCTGTCCTCCTCGCCAC
4548_Pcsk9_OT8_F	CTACACGACGCTCTTCCGATCTTTCCGGACCCAGAACAAAGCCC
4574_Pcsk9_OT8_R	AGACGTGTGCTCTTCCGATCTGAGACCTGCATGGACGGGCATG
4556_Pcsk9_OT9_F	CTACACGACGCTCTTCCGATCTCACCCCATCACCCGCTCAACTC
4582_Pcsk9_OT9_R	AGACGTGTGCTCTTCCGATCTCCCTTTCCCAACCACGTGCAGA
4552_Pcsk9_OT10_F	CTACACGACGCTCTTCCGATCTCCCAGTAACACCACCAGCAGCA
4578_Pcsk9_OT10_R	AGACGTGTGCTCTTCCGATCTCTAGATCTGGGGCCGCTGTTGC
4563_Pcsk9_OT11_F	CTACACGACGCTCTTCCGATCTGAGGAAGGACAGGGTCGCGG
4589_Pcsk9_OT11_R	AGACGTGTGCTCTTCCGATCTCCGCAAAGATGGAGGAGCCGT
4553_Pcsk9_OT12_F	CTACACGACGCTCTTCCGATCTTCATTTCCCTCCCCTCCTGCCT
4579_Pcsk9_OT12_R	AGACGTGTGCTCTTCCGATCTGGCAATTCTTTATTATCAATTGAAGCCAGC

4558_Pcsk9_OT13_F	CTACACGACGCTCTTCCGATCTCATCCGCGCGTTTTCACCTGTT
4584_Pcsk9_OT13_R	AGACGTGTGCTCTTCCGATCTGAGCCGGACACTTCAGACCCCT
4564_Pcsk9_OT14_F	CTACACGACGCTCTTCCGATCTGCACTTGCACTGTGGGGAAAAGG
4590_Pcsk9_OT14_R	AGACGTGTGCTCTTCCGATCTTGACTACATTCACTGTGCACCCCA
4560_Pcsk9_OT15_F	CTACACGACGCTCTTCCGATCTGGGACGGTTCAAGTGAAGGGGA
4586_Pcsk9_OT15_R	AGACGTGTGCTCTTCCGATCTGTCAGACCCCAACCCACCCAAA
4561_Pcsk9_OT16_F	CTACACGACGCTCTTCCGATCTTTGCCTGCCCTGGTTTCTTGCT
4587_Pcsk9_OT16_R	AGACGTGTGCTCTTCCGATCTGCCCAGAGCACTGTGGCTACTC
4569_Pcsk9_OT17_F	CTACACGACGCTCTTCCGATCTGGGCCTCCAGAACTTGACCAGC
4595_Pcsk9_OT17_R	AGACGTGTGCTCTTCCGATCTAGATATTCCTCGCGGCGGCATC
4555_Pcsk9_OT18_F	CTACACGACGCTCTTCCGATCTTAAGGCTGTGACCCTGCTGTGC
4581_Pcsk9_OT18_R	AGACGTGTGCTCTTCCGATCTCTCCTGGTCCAGCAGTTGTCCC
4554_Pcsk9_OT19_F	CTACACGACGCTCTTCCGATCTAGCTGGATCAAGGCATGGAGGC
4580_Pcsk9_OT19_R	AGACGTGTGCTCTTCCGATCTTGGTGGTTCTAGCTGCTGTGGC
4562_Pcsk9_OT20_F	CTACACGACGCTCTTCCGATCTTGTTTAAAGGCTTTGAAACTCACAGG
4588_Pcsk9_OT20_R	AGACGTGTGCTCTTCCGATCTAGAAAGTCTCAGTGTCTGTTAGTGGA
4708_Pcsk9_OT21_F	CTACACGACGCTCTTCCGATCTGTGGGGACAAAGATGAAATAGAAGC
4709_Pcsk9_OT21_R	AGACGTGTGCTCTTCCGATCTACCATCTTGTGGTGGTTCTAGCTG
4571_Pcsk9_OT22_F	CTACACGACGCTCTTCCGATCTGGTGCTCTTGCTGATGTTCCTGC
4597_Pcsk9_OT22_R	AGACGTGTGCTCTTCCGATCTAGCACGAGCAGTAGCAAAAGCA
4568_Pcsk9_OT24_F	CTACACGACGCTCTTCCGATCTGAGAGGTGGGGAAGAGCTGGGA
4594_Pcsk9_OT24_R	AGACGTGTGCTCTTCCGATCTGTGGCTGAAAGTGAGCTGGGGA
4570_Pcsk9_OT27_F	CTACACGACGCTCTTCCGATCTGGACCACCAGTGTGCTTGGACT
4596_Pcsk9_OT27_R	AGACGTGTGCTCTTCCGATCTTGCAGCAGGTGTTTGATGTGTGC
4710_Pcsk9_OT29_F	CTACACGACGCTCTTCCGATCTGGAGCACAGGAAGTTGGGACT
4711_Pcsk9_OT29_R	AGACGTGTGCTCTTCCGATCTCAAGATGAGCGACGTGAGCC
4712_Pcsk9_OT44_F	CTACACGACGCTCTTCCGATCTGCAGCAAGTCTAATCTTCAATGTCA
4713_Pcsk9_OT44_R	AGACGTGTGCTCTTCCGATCTTCTTGAGAGGGTATGAATGGGAG
4714_Pcsk9_OT46_F	CTACACGACGCTCTTCCGATCTTGTCCTTAGATATTGCTCTGTTCTTCTG
4715_Pcsk9_OT46_R	AGACGTGTGCTCTTCCGATCTGGGAAACCTCAGTGCCAGTGTC
4716_Pcsk9_OT47_F	CTACACGACGCTCTTCCGATCTAACACATAAACAAAAGGTCTGAATCTA
4717_Pcsk9_OT47_R	AGACGTGTGCTCTTCCGATCTCATCTTGTGGTGGTTCTAGTTGC
4718_Pcsk9_OT48_F	CTACACGACGCTCTTCCGATCTTTGGGGCACACACGGGTCA
4719_Pcsk9_OT48_R	AGACGTGTGCTCTTCCGATCTCCAGAATGGTCACGGTCAGGG
4720_Pcsk9_OT49_F	CTACACGACGCTCTTCCGATCTTCAGCCGCTCCTCACCTGG
4721_Pcsk9_OT49_R	AGACGTGTGCTCTTCCGATCTGAAAAGCCCGTGAAACAAGAGGA
4722_Pcsk9_OT50_F	CTACACGACGCTCTTCCGATCTTTGGCTCCCTGCCTCCTAATCC
4723_Pcsk9_OT50_R	AGACGTGTGCTCTTCCGATCTTGGTGGCTGCTTCTGGGAGG
4724_Pcsk9_OT51_F	CTACACGACGCTCTTCCGATCTTGGAGCCAGAACCAGGGAGA
4725_Pcsk9_OT51_R	AGACGTGTGCTCTTCCGATCTTTGCTCCTTCCTTGGCTACTGG
4726_Pcsk9_OT52_F	CTACACGACGCTCTTCCGATCTCTGCGAACATTGCCTGGG
4727_Pcsk9_OT52_R	AGACGTGTGCTCTTCCGATCTTGCTGGGCCTAGAGAATCACAC
4728_Pcsk9_OT53_F	CTACACGACGCTCTTCCGATCTCGATAAGACGGCTATAAAATCCC
4729_Pcsk9_OT53_R	AGACGTGTGCTCTTCCGATCTCTCTGAGAAGTTTCCGCTAAGTTGT
4730_Pcsk9_OT54_F	CTACACGACGCTCTTCCGATCTTTGTTGAGGGGGACGGAGGAC
4731_Pcsk9_OT54_R	AGACGTGTGCTCTTCCGATCTAATGAAAGGAAAAGCAGGGTCTGTG
4732_Pcsk9_OT56_F	CTACACGACGCTCTTCCGATCTGAGTGAGTTCCAGGACAGCCAG
4733_Pcsk9_OT56_R	AGACGTGTGCTCTTCCGATCTTAAGAGAGGCTGGGGTCAATAATG

4734_Pcsk9_OT57_F	CTACACGACGCTCTTCCGATCTCGGTAGCAAGTGGTTTGGCAG
4735_Pcsk9_OT57_R	AGACGTGTGCTCTTCCGATCTACCTTCCTGTGGTAGCTCCAGCT
4736_Pcsk9_OT59_F	CTACACGACGCTCTTCCGATCTTGTATGAGGAGCCAGTGACATGA
4737_Pcsk9_OT59_R	AGACGTGTGCTCTTCCGATCTGGGTGGGAACAAAGGAGGTG
4738_Pcsk9_OT63_F	CTACACGACGCTCTTCCGATCTAGCCTTTAGGAGCTGTCCTTCAG
4739_Pcsk9_OT63_R	AGACGTGTGCTCTTCCGATCTCAGGGGGAAGAAAGTGGATTGTA
4740_Pcsk9_OT65_F	CTACACGACGCTCTTCCGATCTCACCTCCTAAACTCCTTCCCCTAAC
4741_Pcsk9_OT65_R	AGACGTGTGCTCTTCCGATCTAAGCAGGAGTGGGTGGGTTG
4742_Pcsk9_OT66_F	CTACACGACGCTCTTCCGATCTGGCTGCCCTGCTGGTGTCTG
4743_Pcsk9_OT66_R	AGACGTGTGCTCTTCCGATCTGGATGGGTCCAGTTCCTTTAGCAG
4744_Pcsk9_OT67_F	CTACACGACGCTCTTCCGATCTTGGCTGTCCAGTGGTTTGTG
4745_Pcsk9_OT67_R	AGACGTGTGCTCTTCCGATCTCTTTGGCAATCAGTATGCGAGT
4746_Pcsk9_OT69_F	CTACACGACGCTCTTCCGATCTTTGAGGATCTCCTCCACTGTGCC
4747_Pcsk9_OT69_R	AGACGTGTGCTCTTCCGATCTGCGGTCCAGTCCTGCCCC
4748_Pcsk9_OT75_F	CTACACGACGCTCTTCCGATCTATGGTTGACAGCAGTTGGAGATG
4749_Pcsk9_OT75_R	AGACGTGTGCTCTTCCGATCTGCTCCTGTTGTGGCTCTGTCTG
4750_Pcsk9_OT79_F	CTACACGACGCTCTTCCGATCTTTACCAAGATCTCTGTCCACTCTCTT
4751_Pcsk9_OT79_R	AGACGTGTGCTCTTCCGATCTCTAGAGTCATATTCTGAGGAAGGGACA
4752_Pcsk9_OT87_F	CTACACGACGCTCTTCCGATCTCATCTTGGTGGCAGCATTTGTG
4753_Pcsk9_OT87_R	AGACGTGTGCTCTTCCGATCTGCACAGGCTTATGGAACACAATCA
4754_Pcsk9_OT96_F	CTACACGACGCTCTTCCGATCTAGGTGTCAAGGGACTCTGTGCTC
4755_Pcsk9_OT96_R	AGACGTGTGCTCTTCCGATCTAGGACATAATCTGCTACCTACTGACATC
4756_Pcsk9_OT104_F	CTACACGACGCTCTTCCGATCTCTACCTACATTTTCATCTGATTCCTCTG
4757_Pcsk9_OT104_R	AGACGTGTGCTCTTCCGATCTTTTGATGTGTGTATCTGTCGGTCTG
4764_Pcsk9_OT108_F	CTACACGACGCTCTTCCGATCTCACCTGTTTTCTGGTATTGGCTTGTC
4765_Pcsk9_OT108_R	AGACGTGTGCTCTTCCGATCTCCAGACACACAAAACAAGGCTTACAAT
4766_Pcsk9_OT112_F	CTACACGACGCTCTTCCGATCTACTCTGAAAAAGACAGTTTAGTGGCA
4767_Pcsk9_OT112_R	AGACGTGTGCTCTTCCGATCTGAAGGAAATCACATTTAAAGAGACAGCT
4768_Pcsk9_OT120_F	CTACACGACGCTCTTCCGATCTAGAGATGCTGCCTTTCCCCACT
4769_Pcsk9_OT120_R	AGACGTGTGCTCTTCCGATCTTTTCACCACACACTTTTGGACCC
4758_Pcsk9_OT143_F	CTACACGACGCTCTTCCGATCTAAGGGGAAATGAGTGTAAAAGGGAG
4759_Pcsk9_OT143_R	AGACGTGTGCTCTTCCGATCTAGGGACCGACAGGGCTTGAG
4760_Pcsk9_OT170_F	CTACACGACGCTCTTCCGATCTACTAAAGGGCAGAACTCCATCAGC
4761_Pcsk9_OT170_R	AGACGTGTGCTCTTCCGATCTCTCTACCTGCTTGCCTCTCCTGG
4762_Pcsk9_OT177_F	CTACACGACGCTCTTCCGATCTAAGGGTGGCTTTTGACTCTAAGTCC
4763_Pcsk9_OT177_R	AGACGTGTGCTCTTCCGATCTTCCTGATTGCTGGAGTGTTACCC
4762_Pcsk9_OT234_F	CTACACGACGCTCTTCCGATCTAACTGGTAGCACCTTCTATGCCTCT
4763_Pcsk9_OT234_R	AGACGTGTGCTCTTCCGATCTACCATCTCTAAAGCTCACCACAGG
4762_Pcsk9_OT235_F	CTACACGACGCTCTTCCGATCTGGACTCCTTTAATTACAGGGTCAATG
4763_Pcsk9_OT235_R	AGACGTGTGCTCTTCCGATCTTTTCTCCTCCAAGCCCACCA
5098_Actb_on_F	CTACACGACGCTCTTCCGATCTCCAAGTATCCATGAAATAAGTGGTTACAGG
5099_Actb_on_R	AGACGTGTGCTCTTCCGATCTCTTCATACATCAAGTTGGGGGGACA
5100_Actb_OT1_F	CTACACGACGCTCTTCCGATCTAAATGAAGCATTAAGGCGGAAGA

5101_Actb_OT1_R	AGACGTGTGCTCTTCCGATCTTGGAACGGCGAAGGCAAC
5102_Actb_OT2_F	CTACACGACGCTCTTCCGATCTATAAAAGTGAAGAAGTATTAAGGTGGAAG
5103_Actb_OT2_R	AGACGTGTGCTCTTCCGATCTGGTGGCTGAGGATTTGGATTG
5104_Actb_OT3_F	CTACACGACGCTCTTCCGATCTCACCCTCCGCCTGCTCATAG
5105_Actb_OT3_R	AGACGTGTGCTCTTCCGATCTGGGCTCATCTGCCAGACCAC
5106_Actb_OT4_F	CTACACGACGCTCTTCCGATCTTGGGCTGAGGCAGGAATGAG
5107_Actb_OT4_R	AGACGTGTGCTCTTCCGATCTGGCTCAGAGTCTTTCCGTCAGG
5108_Actb_OT5_F	CTACACGACGCTCTTCCGATCTAAAGTTCTACAATGTGGCTGAGGAC
5109_Actb_OT5_R	AGACGTGTGCTCTTCCGATCTACAGAAGCAATGCTGTCACCTTC
5110_Actb_OT6_F	CTACACGACGCTCTTCCGATCTAAAAATGAGGAAGTATTACTGTGGAAG
5111_Actb_OT6_R	AGACGTGTGCTCTTCCGATCTGTGAAGCGGTCGGTTGGAG
5112_Actb_OT7_F	CTACACGACGCTCTTCCGATCTCAAGGACAAGGAAGTGGTGGAAC
5113_Actb_OT7_R	AGACGTGTGCTCTTCCGATCTCTGTCCCAGGCTGAGCACTTG
5114_Actb_OT8_F	CTACACGACGCTCTTCCGATCTGAGTTATCACCTCCCTTCATAGTCTTC
5115_Actb_OT8_R	AGACGTGTGCTCTTCCGATCTAGATGCCTCTAACCAAACCAATG
5116_Actb_OT9_F	CTACACGACGCTCTTCCGATCTGTCTACATTATGAAAAACCCATTACTGC
5117_Actb_OT9_R	AGACGTGTGCTCTTCCGATCTACACAGCCAGGCACCAGATG
5118_Actb_OT10_F	CTACACGACGCTCTTCCGATCTTCATCAAGTATGATTGGAAGGGT
5119_Actb_OT10_R	AGACGTGTGCTCTTCCGATCTCTCCTGAAGAAGTAAGCAATAAAGC
5120_Actb_OT11_F	CTACACGACGCTCTTCCGATCTGCAATGCTGTCATCTCTTTGGTGTAG
5121_Actb_OT11_R	AGACGTGTGCTCTTCCGATCTAGTCAGTTGTAGCAAGCATCCCC
5122_Actb_OT12_F	CTACACGACGCTCTTCCGATCTCTCTGTGACTAGCCAACTGCCTG
5123_Actb_OT12_R	AGACGTGTGCTCTTCCGATCTTGGATGTAGGGGTCTTCTAATGAGTA

Adapter oligo sequences used to anneal and complexing with the Tn5: Oligos were ordered from Integrated DNA Technologies (IDT)

Blue: i5 Barcode sequence
Red: hyperactive mosaic end for Tn5 transposase recognition

Oligo Name	sequence 5'-3'		
Tn5-A bottom	[Phos]CTGTCTCTTATACA[ddC]	**i5 Barcode Sequence**	**i5 Barcode READ (Tx10 for UMI)**
i5_N501_UMI	AATGATACGGCGACCACCGAGATCTACACTAGATCGCNNNNNNNNNNTCGTCGGCAGCGTCAGATGTGTATAAGAGACAG	TAGATCGC	TAGATCGCTTTTTTTTTT
i5_N502_UMI	AATGATACGGCGACCACCGAGATCTACACCTCTCTATNNNNNNNNNNTCGTCGGCAGCGTCAGATGTGTATAAGAGACAG	CTCTCTAT	CTCTCTATTTTTTTTTTT
i5_N503_UMI	AATGATACGGCGACCACCGAGATCTACACTATCCTCTNNNNNNNNNNTCGTCGGCAGCGTCAGATGTGTATAAGAGACAG	TATCCTCT	TATCCTCTTTTTTTTTTT
i5_N504_UMI	AATGATACGGCGACCACCGAGATCTACACAGAGTAGANNNNNNNNNNTCGTCGGCAGCGTCAGATGTGTATAAGAGACAG	AGAGTAGA	AGAGTAGATTTTTTTTTT
i5_N505_UMI	AATGATACGGCGACCACCGAGATCTACACGTAAGGAGNNNNNNNNNNTCGTCGGCAGCGTCAGATGTGTATAAGAGACAG	GTAAGGAG	GTAAGGAGTTTTTTTTTT
i5_N506_UMI	AATGATACGGCGACCACCGAGATCTACACACTGCATANNNNNNNNNNTCGTCGGCAGCGTCAGATGTGTATAAGAGACAG	ACTGCATA	ACTGCATATTTTTTTTTT
i5_N507_UMI	AATGATACGGCGACCACCGAGATCTACACAAGGAGTANNNNNNNNNNTCGTCGGCAGCGTCAGATGTGTATAAGAGACAG	AAGGAGTA	AAGGAGTATTTTTTTTTT
i5_N508_UMI	AATGATACGGCGACCACCGAGATCTACACCTAAGCCTNNNNNNNNNNTCGTCGGCAGCGTCAGATGTGTATAAGAGACAG	CTAAGCCT	CTAAGCCTTTTTTTTTTT

i5 oligo sequence for Round 1 and 2 PCR:

Oligo Name	sequence 5'-3'
i5	AATGATACGGCGACCACCGAGATCTACAC

i7 Barcoded oligo sequences for Round 2 PCR:

Oligo Name	Sequence (5'-3')	i7 Barcode Sequence	i7 BC READ
i7_N701	CAAGCAGAAGACGGCATACGAGATAGCGGAATGTGACTGGAG TTCAGACGTGT	AGCGGAAT	ATTCCG CT
i7_N702	CAAGCAGAAGACGGCATACGAGATGATCATGCGTGACTGGAG TTCAGACGTGT	GATCATGC	GCATGA TG
i7_N703	CAAGCAGAAGACGGCATACGAGATAAGACGGAGTGACTGGA GTTCAGACGTGT	AAGACGGA	TCCGTC TT
i7_N704	CAAGCAGAAGACGGCATACGAGATCGAGTCCTGTGACTGGAG TTCAGACGTGT	CGAGTCCT	AGGACT CG
i7_N705	CAAGCAGAAGACGGCATACGAGATTCCTCAGGGTGACTGGAG TTCAGACGTGT	TCCTCAGG	CCTGAG GA
i7_N706	CAAGCAGAAGACGGCATACGAGATGTACGGATGTGACTGGAG TTCAGACGTGT	GTACGGAT	ATCCGT AC
i7_N707	CAAGCAGAAGACGGCATACGAGATCATCTCTCGTGACTGGAG TTCAGACGTGT	CATCTCTC	GAGAGA TG
i7_N710	CAAGCAGAAGACGGCATACGAGATGTCGGAGCGTGACTGGAG TTCAGACGTGT	GTCGGAGC	GCTCCG AC
i7_N711	CAAGCAGAAGACGGCATACGAGATACGGAGAAGTGACTGGA GTTCAGACGTGT	ACGGAGAA	TTCTCC GT
i7_N712	CAAGCAGAAGACGGCATACGAGATAGGAGATGGTGACTGGA GTTCAGACGTGT	AGGAGATG	CATCTC CT
i7_N714	CAAGCAGAAGACGGCATACGAGATAGTACTCGGTGACTGGAG TTCAGACGTGT	AGTACTCG	CGAGTA CT
i7_N715	CAAGCAGAAGACGGCATACGAGATGGACTCTAGTGACTGGAG TTCAGACGTGT	GGACTCTA	TAGAGT CC

Supplementary Table 4. *In vivo* on target analysis at Fah site by UdiTaS.

	Total		Indels		Forward_insertion		Reverse_insertion	
**Fah_Locus_F**	UMI	Total	UMI	Total	UMI	Total	UMI	Total
Cas9-mSA+biotin Fah exon2-14_D34_R1	11659	536301	348	15987	1573	72371	102	4711
Cas9-mSA+biotin Fah exon2-14_D34_R2	8545	393081	214	9833	982	45149	79	3623
Cas9-mSA+biotin Fah exon2-14_D34_R3	7442	342337	152	7010	726	33379	54	2477
Cas9-mSA+biotin Fah exon2-14_D0_R1	9944	457415	661	30407	301	13861	261	12014
Cas9-mSA+biotin Fah exon2-14_D0_R2	12401	570434	1067	49063	362	16652	264	12158
Cas9-mSA+biotin Fah exon2-14_D0_R3	13852	637179	1121	51562	347	15956	278	12801
biotin Fah exon2-14_D0_R1	8189	376692	4	17	2	11	6	28
biotin Fah exon2-14_D0_R2	14337	659524	6	29	3	13	4	20
biotin Fah exon2-14_D0_R3	12283	564999	6	25	2	9	0	0

	Total		Indels		Forward_insertion		Reverse_insertion	
**Fah_Locus_R**	UMI	Total	UMI	Total	UMI	Total	UMI	Total
Cas9-mSA+biotin Fah exon2-14_D34_R1	3848	158788	135	3781	545	23229	42	*23229*
Cas9-mSA+biotin Fah exon2-14_D34_R2	2314	106091	62	2321	252	9151	31	9151
Cas9-mSA+biotin Fah exon2-14_D34_R3	2904	137508	66	4552	280	14856	33	14856
Cas9-mSA+biotin Fah exon2-14_D0_R1	3235	138303	226	9965	94	3153	71	3153
Cas9-mSA+biotin Fah exon2-14_D0_R2	4026	179875	293	16159	124	4754	89	4754
Cas9-mSA+biotin Fah exon2-14_D0_R3	4677	189455	349	17497	135	5037	112	5037
biotin Fah exon2-14_D0_R1	2704	122660	1	3	1	3	2	3
biotin Fah exon2-14_D0_R2	4415	215671	2	8	1	4	1	4
biotin Fah exon2-14_D0_R3	4001	161822	2	7	1	2	0	0

	Forward_insertion		Imprecise		precise		Imprecise %	precise %
**Fah_Locus_F**	UMI	Total	UMI	Total	UMI	Total		
Cas9-mSA+biotin Fah exon2-14_D34_R1	1573	72371	1509	70658	64	1713	95.93%	4.07%
Cas9-mSA+biotin Fah exon2-14_D34_R2	982	45149	955	44080	27	1069	97.25%	2.75%
Cas9-mSA+biotin Fah exon2-14_D34_R3	726	33379	697	32589	29	790	96.01%	3.99%
Cas9-mSA+biotin Fah exon2-14_D0_R1	301	13861	293	13533	8	328	97.34%	2.66%
Cas9-mSA+biotin Fah exon2-14_D0_R2	362	16652	356	16258	6	394	98.34%	1.66%
Cas9-mSA+biotin Fah exon2-14_D0_R3	347	15956	339	15578	8	378	97.69%	2.31%

	Forward_insertion		Imprecise		precise		Imprecise %	precise %
**Fah_Locus_R**	UMI	Total	UMI	Total	UMI	Total		
Cas9-mSA+biotin Fah exon2-14_D34_R1	545	23229	524	23182	21	47	96.15%	3.85%
Cas9-mSA+biotin Fah exon2-14_D34_R2	252	9151	243	8546	9	605	96.43%	3.57%
Cas9-mSA+biotin Fah exon2-14_D34_R3	280	14856	271	14510	9	346	96.79%	3.21%
Cas9-mSA+biotin Fah exon2-14_D0_R1	94	3153	93	2914	1	239	98.94%	1.06%
Cas9-mSA+biotin Fah exon2-14_D0_R2	124	4754	121	4237	3	517	97.58%	2.42%
Cas9-mSA+biotin Fah exon2-14_D0_R3	135	5037	132	4599	3	438	97.78%	2.22%

Supplementary Sequences 1. Sequences of donors used in this study

biotin-iGUIDE donor
bio/G*C*TCGCGTTTAATTGAGTTGTCATATGTTAATAACGGTATACGC*G*A

phospho-iGUIDE donor
p-G*C*TCGCGTTTAATTGAGTTGTCATATGTTAATAACGGTATACGC*G*A

biotin-GUIDE donor
bio/G*T*TTAATTGAGTTGTCATATGTTAATAACGGT*A*T

phospho-GUIDE donor
p-G*T*TTAATTGAGTTGTCATATGTTAATAACGGT*A*T

IRES-GFP-pA (1.7Kb)
GCCGCTCTAGAACTAGTGGATCCGAGATCGAGTGCCGCATCACCGGCAATTGACGCGTGCTCCTCTCCCTCCCCCCCCCCT
AACGTTACTGGCCGAAGCCGCTTGGAATAAGGCCGGTGTGCGTTTGTCTATATGTTATTTTCCACCATATTGCCGTCTTTT
GGCAATGTGAGGGCCCGGAAACCTGGCCCTGTCTTCTTGACGAGCATTCCTAGGGGTCTTTCCCCTCTCGCCAAAGGAATG
CAAGGTCTGTTGAATGTCGTGAAGGAAGCAGTTCCTCTGGAAGCTTCTTGAAGACAAACAACGTCTGTAGCGACCCTTTGC
AGGCAGCGGAACCCCCCACCTGGCGACAGGTGCCTCTGCGGCCAAAAGCCACGTGTATAAGATACACCTGCAAAGGCGGCA
CAACCCCAGTGCCACGTTGTGAGTTGGATAGTTGTGGAAAGAGTCAAATGGCTCTCCTCAAGCGTATTCAACAAGGGGCTG
AAGGATGCCCAGAAGGTACCCCATTGTATGGGATCTGATCTGGGGCCTCGGTACACATGCTTTACATGTGTTTAGTCGAGG
TTAAAAAAACGTCTAGGCCCCCCGAACCACGGGGACGTGGTTTTCCTTTGAAAAACACGATGATAATATGGCCACAATGGT
GAGCAAGGGCGAGGAGCTGTTCACCGGGGTGGTGCCCATCCTGGTCGAGCTGGACGGCGACGTAAACGGCCACAAGTTCAG
CGTGTCCGGCGAGGGCGAGGGCGATGCCACCTACGGCAAGCTGACCCTGAAGTTCATCTGCACCACCGGCAAGCTGCCCGT
GCCCTGGCCCACCCTCGTGACCACCCTGACCTACGGCGTGCAGTGCTTCAGCCGCTACCCCGACCACATGAAGCAGCACGA
CTTCTTCAAGTCCGCCATGCCCGAAGGCTACGTCCAGGAGCGCACCATCTTCTTCAAGGACGACGGCAACTACAAGACCCG
CGCCGAGGTGAAGTTCGAGGGCGACACCCTGGTGAACCGCATCGAGCTGAAGGGCATCGACTTCAAGGAGGACGGCAACAT
CCTGGGGCACAAGCTGGAGTACAACTACAACAGCCACAACGTCTATATCATGGCCGACAAGCAGAAGAACGGCATCAAGGT
GAACTTCAAGATCCGCCACAACATCGAGGACGGCAGCGTGCAGCTCGCCGACCACTACCAGCAGAACACCCCCATCGGCGA
CGGCCCCGTGCTGCTGCCCGACAACCACTACCTGAGCACCCAGTCCGCCCTGAGCAAAGACCCCAACGAGAAGCGCGATCA
CATGGTCCTGCTGGAGTTCGTGACCGCCGCCGGGATCACTCTCGGCATGGACGAGCTGTACAAGTAAATGCATCGATGATC
TAGAGCTCGCTGATCAGCCTCGACTGTGCCTTCTAGTTGCCAGCTATCTGTTGTTTGCCCCTCCCCCGTGCCTTCCTTGAC
CCTGGAAGGTGCCACTCCCACTGTCCTTTCCTAATAAAATGAGGAAATTGCATCGCATTGTCTGAGTAGGTGTCATTCTAT
TCTGGGGGGTGGGGTGGGGCAGGACAGCAAGGGGGAGGATTGGGAAGACAATAGCAGGCATGCTGGGGATGCGGTGGGCTC
TATGGATAACTTCGTATAGCATACATTATACGAAGTTATCGGAACCCTTAATGTCGAGGGGGGGCCCGGTACCAGCTTTTG
TTCCCTTTAGTGAGGGTTAATTGCGCGCTTGGCGTAATCATGGTCATAGCTGTTTCCTGTGTGAAATTGTTATCCGCT

IRES-GFP-pA (2.5Kb)

GCCGCTCTAGAACTAGTGGATCCGAGATCGAGTGCCGCATCACCGGCAATTGACGCGTGCTCCTCTCCCTCCCCCCCCCCT
AACGTTACTGGCCGAAGCCGCTTGGAATAAGGCCGGTGTGCGTTTGTCTATATGTTATTTTCCACCATATTGCCGTCTTTT
GGCAATGTGAGGGCCCGGAAACCTGGCCCTGTCTTCTTGACGAGCATTCCTAGGGGTCTTTCCCCTCTCGCCAAAGGAATG
CAAGGTCTGTTGAATGTCGTGAAGGAAGCAGTTCCTCTGGAAGCTTCTTGAAGACAAACAACGTCTGTAGCGACCCTTTGC
AGGCAGCGGAACCCCCCACCTGGCGACAGGTGCCTCTGCGGCCAAAAGCCACGTGTATAAGATACACCTGCAAAGGCGGCA
CAACCCCAGTGCCACGTTGTGAGTTGGATAGTTGTGGAAAGAGTCAAATGGCTCTCCTCAAGCGTATTCAACAAGGGGCTG
AAGGATGCCCAGAAGGTACCCCATTGTATGGGATCTGATCTGGGGCCTCGGTACACATGCTTTACATGTGTTTAGTCGAGG
TTAAAAAAACGTCTAGGCCCCCCGAACCACGGGGACGTGGTTTTCCTTTGAAAAACACGATGATAATATGGCCACAACCAT
GGTGAGCAAGGGCGAGGAGCTGTTCACCGGGGTGGTGCCCATCCTGGTCGAGCTGGACGGCGACGTAAACGGCCACAAGTT
CAGCGTGTCCGGCGAGGGCGAGGGCGATGCCACCTACGGCAAGCTGACCCTGAAGTTCATCTGCACCACCGGCAAGCTGCC
CGTGCCCTGGCCCACCCTCGTGACCACCCTGACCTACGGCGTGCAGTGCTTCAGCCGCTACCCCGACCACATGAAGCAGCA
CGACTTCTTCAAGTCCGCCATGCCCGAAGGCTACGTCCAGGAGCGCACCATCTTCTTCAAGGACGACGGCAACTACAAGAC
CCGCGCCGAGGTGAAGTTCGAGGGCGACACCCTGGTGAACCGCATCGAGCTGAAGGGCATCGACTTCAAGGAGGACGGCAA
CATCCTGGGGCACAAGCTGGAGTACAACTACAACAGCCACAACGTCTATATCATGGCCGACAAGCAGAAGAACGGCATCAA
GGTGAACTTCAAGATCCGCCACAACATCGAGGACGGCAGCGTGCAGCTCGCCGACCACTACCAGCAGAACACCCCCATCGG
CGACGGCCCCGTGCTGCTGCCCGACAACCACTACCTGAGCACCCAGTCCGCCCTGAGCAAAGACCCCAACGAGAAGCGCGA
TCACATGGTCCTGCTGGAGTTCGTGACCGCCGCCGGGATCACTCTCGGCATGGACGAGCTGTACAAGTAAATGCATCGATG

ATCTAGAGCTCGCTGATCAGCCTCGACTGTGCCTTCTAGTTGCCAGCTATCTGTTGTTTGCCCCTCCCCCGTGCCTTCCTT
GACCCTGGAAGGTGCCACTCCCACTGTCCTTTCCTAATAAAATGAGGAAATTGCATCGCATTGTCTGAGTAGGTGTCATTC
TATTCTGGGGGGTGGGGTGGGGCAGGACAGCAAGGGGGAGGATTGGGAAGACAATAGCAGGCATGCTGGGGATGCGGTGGG
CTCTATGGATAACTTCGTATAGCATACATTATACGAAGTTATCGGAACCCTTAATGTCGAGGGGGGGCCCGGTACCAGCTT
TTGTTCCCTTTAGTGAGGGTTAATTGCGCGCTTGGCGTAATCATGGTCATAGCTGTTTCCTGTGTGAAATTGTTATCCGCT
CACAATTCCACACAACATACGAGCCGGAAGCATAAAGTGTAAAGCCTGGGGTGCCTAATGAGTGAGCTAACTCACATTAAT
TGCGTTGCGCTCACTGCCCGCTTTCCAGTCGGGAAACCTGTCGTGCCAGCTGCATTAATGAATCGGCCAACGCGCGGGGAG
AGGCGGTTTGCGTATTGGGCGCTCTTCCGCTTCCTCGCTCACTGACTCGCTGCGCTCGGTCGTTCGGCTGCGGCGAGCGGT
ATCAGCTCACTCAAAGGCGGTAATACGGTTATCCACAGAATCAGGGGATAACGCAGGAAAGAACATGTGAGCAAAAGGCCA
GCAAAAGGCCAGGAACCGTAAAAAGGCCGCGTTGCTGGCGTTTTTCCATAGGCTCCGCCCCCCTGACGAGCATCACAAAAA
TCGACGCTCAAGTCAGAGGTGGCGAAACCCGACAGGACTATAAAGATACCAGGCGTTTCCCCCTGGAAGCTCCCTCGTGCG
CTCTCCTGTTCCGACCCTGCCGCTTACCGGATACCTGTCCGCCTTTCTCCCTTCGGGAAGCGTGGCGCTTTCTCATAGCTC
ACGCTGTAGGTATCTCAGTTCGGTGTAGGTCGTTCGCTCCAAGCTGGGCTGTGTGCACGAACCCCCCGTTCAGCCCGACCG
CTGCGCCTTATCCGGTAACTATCGTCTTGAGTCCAACCCGGTAAGACACGACTTATCGCCACTGG

IRES-GFP-pA (3.5Kb)

GCCGCTCTAGAACTAGTGGATCCGAGATCGAGTGCCGCATCACCGGCAATTGACGCGTGCTCCTCTCCCTCCCCCCCCCCT
AACGTTACTGGCCGAAGCCGCTTGGAATAAGGCCGGTGTGCGTTTGTCTATATGTTATTTTCCACCATATTGCCGTCTTTT
GGCAATGTGAGGGCCCGGAAACCTGGCCCTGTCTTCTTGACGAGCATTCCTAGGGGTCTTTCCCCTCTCGCCAAAGGAATG
CAAGGTCTGTTGAATGTCGTGAAGGAAGCAGTTCCTCTGGAAGCTTCTTGAAGACAAACAACGTCTGTAGCGACCCTTTGC
AGGCAGCGGAACCCCCCACCTGGCGACAGGTGCCTCTGCGGCCAAAAGCCACGTGTATAAGATACACCTGCAAAGGCGGCA
CAACCCCAGTGCCACGTTGTGAGTTGGATAGTTGTGGAAAGAGTCAAATGGCTCTCCTCAAGCGTATTCAACAAGGGGCTG
AAGGATGCCCAGAAGGTACCCCATTGTATGGGATCTGATCTGGGGCCTCGGTACACATGCTTTACATGTGTTTAGTCGAGG
TTAAAAAAACGTCTAGGCCCCCCGAACCACGGGGACGTGGTTTTCCTTTGAAAAACACGATGATAATATGGCCACAACCAT
GGTGAGCAAGGGCGAGGAGCTGTTCACCGGGGTGGTGCCCATCCTGGTCGAGCTGGACGGCGACGTAAACGGCCACAAGTT
CAGCGTGTCCGGCGAGGGCGAGGGCGATGCCACCTACGGCAAGCTGACCCTGAAGTTCATCTGCACCACCGGCAAGCTGCC
CGTGCCCTGGCCCACCCTCGTGACCACCCTGACCTACGGCGTGCAGTGCTTCAGCCGCTACCCCGACCACATGAAGCAGCA
CGACTTCTTCAAGTCCGCCATGCCCGAAGGCTACGTCCAGGAGCGCACCATCTTCTTCAAGGACGACGGCAACTACAAGAC
CCGCGCCGAGGTGAAGTTCGAGGGCGACACCCTGGTGAACCGCATCGAGCTGAAGGGCATCGACTTCAAGGAGGACGGCAA
CATCCTGGGGCACAAGCTGGAGTACAACTACAACAGCCACAACGTCTATATCATGGCCGACAAGCAGAAGAACGGCATCAA
GGTGAACTTCAAGATCCGCCACAACATCGAGGACGGCAGCGTGCAGCTCGCCGACCACTACCAGCAGAACACCCCCATCGG
CGACGGCCCCGTGCTGCTGCCCGACAACCACTACCTGAGCACCCAGTCCGCCCTGAGCAAAGACCCCAACGAGAAGCGCGA
TCACATGGTCCTGCTGGAGTTCGTGACCGCCGCCGGGATCACTCTCGGCATGGACGAGCTGTACAAGTAAATGCATCGATG
ATCTAGAGCTCGCTGATCAGCCTCGACTGTGCCTTCTAGTTGCCAGCTATCTGTTGTTTGCCCCTCCCCCGTGCCTTCCTT
GACCCTGGAAGGTGCCACTCCCACTGTCCTTTCCTAATAAAATGAGGAAATTGCATCGCATTGTCTGAGTAGGTGTCATTC
TATTCTGGGGGGTGGGGTGGGGCAGGACAGCAAGGGGGAGGATTGGGAAGACAATAGCAGGCATGCTGGGGATGCGGTGGG
CTCTATGGATAACTTCGTATAGCATACATTATACGAAGTTATCGGAACCCTTAATGTCGAGGGGGGGCCCGGTACCAGCTT
TTGTTCCCTTTAGTGAGGGTTAATTGCGCGCTTGGCGTAATCATGGTCATAGCTGTTTCCTGTGTGAAATTGTTATCCGCT
CACAATTCCACACAACATACGAGCCGGAAGCATAAAGTGTAAAGCCTGGGGTGCCTAATGAGTGAGCTAACTCACATTAAT
TGCGTTGCGCTCACTGCCCGCTTTCCAGTCGGGAAACCTGTCGTGCCAGCTGCATTAATGAATCGGCCAACGCGCGGGGAG
AGGCGGTTTGCGTATTGGGCGCTCTTCCGCTTCCTCGCTCACTGACTCGCTGCGCTCGGTCGTTCGGCTGCGGCGAGCGGT
ATCAGCTCACTCAAAGGCGGTAATACGGTTATCCACAGAATCAGGGGATAACGCAGGAAAGAACATGTGAGCAAAAGGCCA
GCAAAAGGCCAGGAACCGTAAAAAGGCCGCGTTGCTGGCGTTTTTCCATAGGCTCCGCCCCCCTGACGAGCATCACAAAAA
TCGACGCTCAAGTCAGAGGTGGCGAAACCCGACAGGACTATAAAGATACCAGGCGTTTCCCCCTGGAAGCTCCCTCGTGCG
CTCTCCTGTTCCGACCCTGCCGCTTACCGGATACCTGTCCGCCTTTCTCCCTTCGGGAAGCGTGGCGCTTTCTCATAGCTC
ACGCTGTAGGTATCTCAGTTCGGTGTAGGTCGTTCGCTCCAAGCTGGGCTGTGTGCACGAACCCCCCGTTCAGCCCGACCG
CTGCGCCTTATCCGGTAACTATCGTCTTGAGTCCAACCCGGTAAGACACGACTTATCGCCACTGGCAGCAGCCACTGGTAA
CAGGATTAGCAGAGCGAGGTATGTAGGCGGTGCTACAGAGTTCTTGAAGTGGTGGCCTAACTACGGCTACACTAGAAGGAC
AGTATTTGGTATCTGCGCTCTGCTGAAGCCAGTTACCTTCGGAAAAAGAGTTGGTAGCTCTTGATCCGGCAAACAAACCAC
CGCTGGTAGCGGTGGTTTTTTTGTTTGCAAGCAGCAGATTACGCGCAGAAAAAAAGGATCTCAAGAAGATCCTTTGATCTT
TTCTACGGGGTCTGACGCTCAGTGGAACGAAAACTCACGTTAAGGGATTTTGGTCATGAGATTATCAAAAAGGATCTTCAC
CTAGATCCTTTTAAATTAAAAATGAAGTTTTAAATCAATCTAAAGTATATATGAGTAAACTTGGTCTGACAGTTACCAATG
CTTAATCAGTGAGGCACCTATCTCAGCGATCTGTCTATTTCGTTCATCCATAGTTGCCTGACTCCCCGTCGTGTAGATAAC
TACGATACGGGAGGGCTTACCATCTGGCCCCAGTGCTGCAATGATACCGCGAGACCCACGCTCACCGGCTCCAGATTTATC
AGCAATAAACCAGCCAGCCGGAAGGGCCGAGCGCAGAAGTGGTCCTGCAACTTTATCCGCCTCCATCCAGTCTATTAATTG
TTGCCGGGAAGCTAGAGTAAGTAGTTCGCCAGTTAATAGTTTGCGCAACGTTGTTGCCATTGCTACAGGCATCGTGGTGTC
ACGCTCGTCGTTTGGTATGGCTTCATTCAGCTCCGGTTCCCAACGATCAAGGCGAGTTACATGATCCCCCATGTTGTGCAA

AAAAGCGGTTAGCTCCTTCGGTCCTCCGATCGTTGTCAGAAGTAAGTTGGCCGCAGTGTTATCACTCATGGTTATGGCAGC
ACTGCATAATTCTCTTACTGTCATGCCATCCGT

IRES-GFP-pA (4.5Kb)

GCCGCTCTAGAACTAGTGGATCCGAGATCGAGTGCCGCATCACCGGCAATTGACGCGTGCTCCTCTCCCTCCCCCCCCCCT
AACGTTACTGGCCGAAGCCGCTTGGAATAAGGCCGGTGTGCGTTTGTCTATATGTTATTTTCCACCATATTGCCGTCTTTT
GGCAATGTGAGGGCCCGGAAACCTGGCCCTGTCTTCTTGACGAGCATTCCTAGGGGTCTTTCCCCTCTCGCCAAAGGAATG
CAAGGTCTGTTGAATGTCGTGAAGGAAGCAGTTCCTCTGGAAGCTTCTTGAAGACAAACAACGTCTGTAGCGACCCTTTGC
AGGCAGCGGAACCCCCCACCTGGCGACAGGTGCCTCTGCGGCCAAAAGCCACGTGTATAAGATACACCTGCAAAGGCGGCA
CAACCCCAGTGCCACGTTGTGAGTTGGATAGTTGTGGAAAGAGTCAAATGGCTCTCCTCAAGCGTATTCAACAAGGGGCTG
AAGGATGCCCAGAAGGTACCCCATTGTATGGGATCTGATCTGGGGCCTCGGTACACATGCTTTACATGTGTTTAGTCGAGG
TTAAAAAAACGTCTAGGCCCCCCGAACCACGGGGACGTGGTTTTCCTTTGAAAAACACGATGATAATATGGCCACAACCAT
GGTGAGCAAGGGCGAGGAGCTGTTCACCGGGGTGGTGCCCATCCTGGTCGAGCTGGACGGCGACGTAAACGGCCACAAGTT
CAGCGTGTCCGGCGAGGGCGAGGGCGATGCCACCTACGGCAAGCTGACCCTGAAGTTCATCTGCACCACCGGCAAGCTGCC
CGTGCCCTGGCCCACCCTCGTGACCACCCTGACCTACGGCGTGCAGTGCTTCAGCCGCTACCCCGACCACATGAAGCAGCA
CGACTTCTTCAAGTCCGCCATGCCCGAAGGCTACGTCCAGGAGCGCACCATCTTCTTCAAGGACGACGGCAACTACAAGAC
CCGCGCCGAGGTGAAGTTCGAGGGCGACACCCTGGTGAACCGCATCGAGCTGAAGGGCATCGACTTCAAGGAGGACGGCAA
CATCCTGGGGCACAAGCTGGAGTACAACTACAACAGCCACAACGTCTATATCATGGCCGACAAGCAGAAGAACGGCATCAA
GGTGAACTTCAAGATCCGCCACAACATCGAGGACGGCAGCGTGCAGCTCGCCGACCACTACCAGCAGAACACCCCCATCGG
CGACGGCCCCGTGCTGCTGCCCGACAACCACTACCTGAGCACCCAGTCCGCCCTGAGCAAAGACCCCAACGAGAAGCGCGA
TCACATGGTCCTGCTGGAGTTCGTGACCGCCGCCGGGATCACTCTCGGCATGGACGAGCTGTACAAGTAAATGCATCGATG
ATCTAGAGCTCGCTGATCAGCCTCGACTGTGCCTTCTAGTTGCCAGCTATCTGTTGTTTGCCCCTCCCCCGTGCCTTCCTT
GACCCTGGAAGGTGCCACTCCCACTGTCCTTTCCTAATAAAATGAGGAAATTGCATCGCATTGTCTGAGTAGGTGTCATTC
TATTCTGGGGGGTGGGGTGGGGCAGGACAGCAAGGGGGAGGATTGGGAAGACAATAGCAGGCATGCTGGGGATGCGGTGGG
CTCTATGGATAACTTCGTATAGCATACATTATACGAAGTTATCGGAACCCTTAATGTCGAGGGGGGGCCCGGTACCAGCTT
TTGTTCCCTTTAGTGAGGGTTAATTGCGCGCTTGGCGTAATCATGGTCATAGCTGTTTCCTGTGTGAAATTGTTATCCGCT
CACAATTCCACACAACATACGAGCCGGAAGCATAAAGTGTAAAGCCTGGGGTGCCTAATGAGTGAGCTAACTCACATTAAT
TGCGTTGCGCTCACTGCCCGCTTTCCAGTCGGGAAACCTGTCGTGCCAGCTGCATTAATGAATCGGCCAACGCGCGGGGAG
AGGCGGTTTGCGTATTGGGCGCTCTTCCGCTTCCTCGCTCACTGACTCGCTGCGCTCGGTCGTTCGGCTGCGGCGAGCGGT
ATCAGCTCACTCAAAGGCGGTAATACGGTTATCCACAGAATCAGGGGATAACGCAGGAAAGAACATGTGAGCAAAAGGCCA
GCAAAAGGCCAGGAACCGTAAAAAGGCCGCGTTGCTGGCGTTTTTCCATAGGCTCCGCCCCCCTGACGAGCATCACAAAAA
TCGACGCTCAAGTCAGAGGTGGCGAAACCCGACAGGACTATAAAGATACCAGGCGTTTCCCCCTGGAAGCTCCCTCGTGCG
CTCTCCTGTTCCGACCCTGCCGCTTACCGGATACCTGTCCGCCTTTCTCCCTTCGGGAAGCGTGGCGCTTTCTCATAGCTC
ACGCTGTAGGTATCTCAGTTCGGTGTAGGTCGTTCGCTCCAAGCTGGGCTGTGTGCACGAACCCCCCGTTCAGCCCGACCG
CTGCGCCTTATCCGGTAACTATCGTCTTGAGTCCAACCCGGTAAGACACGACTTATCGCCACTGGCAGCAGCCACTGGTAA
CAGGATTAGCAGAGCGAGGTATGTAGGCGGTGCTACAGAGTTCTTGAAGTGGTGGCCTAACTACGGCTACACTAGAAGGAC
AGTATTTGGTATCTGCGCTCTGCTGAAGCCAGTTACCTTCGGAAAAAGAGTTGGTAGCTCTTGATCCGGCAAACAAACCAC
CGCTGGTAGCGGTGGTTTTTTTGTTTGCAAGCAGCAGATTACGCGCAGAAAAAAAGGATCTCAAGAAGATCCTTTGATCTT
TTCTACGGGGTCTGACGCTCAGTGGAACGAAAACTCACGTTAAGGGATTTTGGTCATGAGATTATCAAAAAGGATCTTCAC
CTAGATCCTTTTAAATTAAAAATGAAGTTTTAAATCAATCTAAAGTATATATGAGTAAACTTGGTCTGACAGTTACCAATG
CTTAATCAGTGAGGCACCTATCTCAGCGATCTGTCTATTTCGTTCATCCATAGTTGCCTGACTCCCCGTCGTGTAGATAAC
TACGATACGGGAGGGCTTACCATCTGGCCCCAGTGCTGCAATGATACCGCGAGACCCACGCTCACCGGCTCCAGATTTATC
AGCAATAAACCAGCCAGCCGGAAGGGCCGAGCGCAGAAGTGGTCCTGCAACTTTATCCGCCTCCATCCAGTCTATTAATTG
TTGCCGGGAAGCTAGAGTAAGTAGTTCGCCAGTTAATAGTTTGCGCAACGTTGTTGCCATTGCTACAGGCATCGTGGTGTC
ACGCTCGTCGTTTGGTATGGCTTCATTCAGCTCCGGTTCCCAACGATCAAGGCGAGTTACATGATCCCCCATGTTGTGCAA
AAAAGCGGTTAGCTCCTTCGGTCCTCCGATCGTTGTCAGAAGTAAGTTGGCCGCAGTGTTATCACTCATGGTTATGGCAGC
ACTGCATAATTCTCTTACTGTCATGCCATCCGTAAGATGCTTTTCTGTGACTGGTGAGTACTCAACCAAGTCATTCTGAGA
ATAGTGTATGCGGCGACCGAGTTGCTCTTGCCCGGCGTCAATACGGGATAATACCGCGCCACATAGCAGAACTTTAAAAGT
GCTCATCATTGGAAAACGTTCTTCGGGGCGAAAACTCTCAAGGATCTTACCGCTGTTGAGATCCAGTTCGATGTAACCCAC
TCGTGCACCCAACTGATCTTCAGCATCTTTTACTTTCACCAGCGTTTCTGGGTGAGCAAAAACAGGAAGGCAAAATGCCGC
AAAAAAGGGAATAAGGGCGACACGGAAATGTTGAATACTCATACTCTTCCTTTTTCAATATTATTGAAGCATTTATCAGGG
TTATTGTCTCATGAGCGGATACATATTTGAATGTATTTAGAAAAATAAACAAATAGGGGTTCCGCGCACATTTCCCCGAAA
AGTGCCACCTAAATTGTAAGCGTTAATATTTTGTTAAAATTCGCGTTAAATTTTTGTTAAATCAGCTCATTTTTTAACCAA
TAGGCCGAAATCGGCAAAATCCCTTATAAATCAAAAGAATAGACCGAGATAGGGTTGAGTGTTGTTCCAGTTTGGAACAAG
AGTCCACTATTAAAGAACGTGGACTCCAACGTCAAAGGGCGAAAAACCGTCTATCAGGGCGATGGCCCACTACGTGAACCA
TCACCCTAATCAAGTTTTTTGGGGTCGAGGTGCCGTAAAGCACTAAATCGGAACCCTAAAGGGAGCCCCCGATTTAGAGCT
TGACGGGGAAAGCCGGCGAACGTGGCGAGAAAGGAAGGGAAGAAAGCGAAAGGAGCGGGCGCTAGGGCGCTGGCAAGTGTA

GCGGTCACGCTGCGCGTAACCACCACACCCGCCGCGCTTAATGCGCCGCTACAGGGCGCGTCCCATTCGCCATTCAGGCTG
CGCAACTGTTGGGAAGGGCGATCGGTGCGGGCCTCTT

Fah-exon 2-14: SA-Exon2-14-pA

TTTATAAGGGTTGTCTCAAAGGAAACCATGAGTCCCTAAGTATTTGCTGTTATAGTAAATGTGCTGTGCCTGTCAGAACTG
GCTGTCATTCTTCTCCTAGCCAAAGCCACGGATTGGTGTAGCCATCGGTGACCAGATCTTGGACCTGAGTGTCATTAAACA
CCTCTTTACCGGACCTGCCCTTTCCAAACATCAACATGTCTTCGATGAGACAACTCTCAATAACTTCATGGGTCTGGGTCA
AGCTGCATGGAAGGAGGCAAGAGCATCCTTACAGAACTTACTGTCTGCCAGCCAAGCCCGGCTCAGAGATGACAAGGAGCT
TCGGCAGCGTGCATTCACCTCCCAGGCTTCTGCGACAATGCACCTTCCTGCTACCATAGGAGACTACACGGACTTCTACTC
TTCTCGGCAGCATGCCACCAATGTTGGCATTATGTTCAGAGGCAAGGAGAATGCGCTGTTGCCAAATTGGCTCCACTTACC
TGTGGGATACCATGGCCGAGCTTCCTCCATTGTGGTATCTGGAACCCCGATTCGAAGACCCATGGGGCAGATGAGACCTGA
TAACTCAAAGCCTCCTGTGTATGGTGCCTGCAGACTCTTAGACATGGAGTTGGAAATGGCTTTCTTCGTAGGCCCTGGGAA
CAGATTCGGAGAGCCAATCCCCATTTCCAAAGCCCATGAACACATTTTCGGGATGGTCCTCATGAACGACTGGAGCGCACG
AGACATCCAGCAATGGGAGTACGTCCCACTTGGGCCATTCCTGGGGAAAAGCTTTGGAACCACAATCTCCCCGTGGGTGGT
GCCTATGGATGCCCTCATGCCCTTTGTGGTGCCAAACCCAAAGCAGGACCCCAAGCCCTTGCCATATCTCTGCCACAGCCA
GCCCTACACATTTGATATCAACCTGTCTGTCTCTTTGAAAGGAGAAGGAATGAGCCAGGCGGCTACCATCTGCAGGTCTAA
CTTTAAGCACATGTACTGGACCATGCTGCAGCAACTCACACACCACTCTGTTAATGGATGCAACCTGAGACCTGGGGACCT
CTTGGCTTCTGGAACCATCAGTGGATCAGACCCTGAAAGCTTTGGCTCCATGCTGGAACTGTCCTGGAAGGGAACAAAGGC
CATCGATGTGGAGCAGGGGCAGACCAGGACCTTCCTGCTGGACGGCGATGAAGTCATCATAACAGGTCACTGCCAGGGGGA
CGGCTACCGTGTTGGCTTTGGCCAGTGTGCTGGGAAAGTGCTGCCTGCCCTTTCACCAGCCTGAGCGGCCCTAGAGTCGGG
GCGGCCGGCCGCTTCGAGCAGACATGATAAGATACATTGATGAGTTTGGACAAACCACAACTAGAATGCAGTGAAAAAAAT
GCTTTATTTGTGAAATTTGTGATGCTATTGCTTTATTTGTAACCATTATAAGCTGCAATAAACAAGTTAACAACAACAATT
GCATTCATTTTATGTTTCAGGTTCAGGGGGAGGTGTGGGAGGTTTTTTAAAGCAAGTAAAACCTCTACAAATGTGGTAAAA
TCGATAAGGATCT

Supplementary Sequences 2. Protein Sequences of Cas9-mSA and Cas9-mSA* used in this study

SpyCas9-mSA: FLAG-SV40_NLS-SpyCas9-Igk leader-mSA

MDYKDHDGDYKDHDIDYKDDDDKMAPKKKRKVGIHGVPAADKKYSIGLDIGTNSVGWAVITDEYKVPSKKFKVLGNTDRHS
IKKNLIGALLFDSGETAEATRLKRTARRRYTRRKNRICYLQEIFSNEMAKVDDSFFHRLEESFLVEEDKKHERHPIFGNIV
DEVAYHEKYPTIYHLRKKLVDSTDKADLRLIYLALAHMIKFRGHFLIEGDLNPDNSDVDKLFIQLVQTYNQLFEENPINAS
GVDAKAILSARLSKSRRLENLIAQLPGEKKNGLFGNLIALSLGLTPNFKSNFDLAEDAKLQLSKDTYDDDLDNLLAQIGDQ
YADLFLAAKNLSDAILLSDILRVNTEITKAPLSASMIKRYDEHHQDLTLLKALVRQQLPEKYKEIFFDQSKNGYAGYIDGG
ASQEEFYKFIKPILEKMDGTEELLVKLNREDLLRKQRTFDNGSIPHQIHLGELHAILRRQEDFYPFLKDNREKIEKILTFR
IPYYVGPLARGNSRFAWMTRKSEETITPWNFEEVVDKGASAQSFIERMTNFDKNLPNEKVLPKHSLLYEYFTVYNELTKVK
YVTEGMRKPAFLSGEQKKAIVDLLFKTNRKVTVKQLKEDYFKKIECFDSVEISGVEDRFNASLGTYHDLLKIIKDKDFLDN
EENEDILEDIVLTLTLFEDREMIEERLKTYAHLFDDKVMKQLKRRRYTGWGRLSRKLINGIRDKQSGKTILDFLKSDGFAN
RNFMQLIHDDSLTFKEDIQKAQVSGQGDSLHEHIANLAGSPAIKKGILQTVKVVDELVKVMGRHKPENIVIEMARENQTTQ
KGQKNSRERMKRIEEGIKELGSQILKEHPVENTQLQNEKLYLYYLQNGRDMYVDQELDINRLSDYDVDHIVPQSFLKDDSI
DNKVLTRSDKNRGKSDNVPSEEVVKKMKNYWRQLLNAKLITQRKFDNLTKAERGGLSELDKAGFIKRQLVETRQITKHVAQ
ILDSRMNTKYDENDKLIREVKVITLKSKLVSDFRKDFQFYKVREINNYHHAHDAYLNAVVGTALIKKYPKLESEFVYGDYK
VYDVRKMIAKSEQEIGKATAKYFFYSNIMNFFKTEITLANGEIRKRPLIETNGETGEIVWDKGRDFATVRKVLSMPQVNIV
KKTEVQTGGFSKESILPKRNSDKLIARKKDWDPKKYGGFDSPTVAYSVLVVAKVEKGKSKKLKSVKELLGITIMERSSFEK
NPIDFLEAKGYKEVKKDLIIKLPKYSLFELENGRKRMLASAGELQKGNELALPSKYVNFLYLASHYEKLKGSPEDNEQKQL
FVEQHKHYLDEIIEQISEFSKRVILADANLDKVLSAYNKHRDKPIREQAENIIHLFTLTNLGAPAAFKYFDTTIDRKRYTS
TKEVLDATLIHQSITGLYETRIDLSQLGGDKRPAATKKAGQAKKKKYEFKASRSGSETPGTSESATPESMETDTLLLWVLL
LWVPGSTGDYPYDVPDYAGAQPARSMAEAGITGTWYNQSGSTFTVTAGADGNLTGQYENRAQGTGCQNSPYTLTGRYNGTK
LEWRVEWNNSTENCHSRTEWRGQYQGGAEARINTQWNLTYEGGSGPATEQGQDTFTKVKPSAASGS*

SpyCas9-mSA*: cMYC-NLS-SpyCas9-HA-mSA

MASPAAKRVKLDGGSGGGSGGGSGPAAKRVKLDGGSGGGSGGGSGPLEPAAKRVKLDDKKYSIGLDIGTNSVGWAVITDEY
KVPSKKFKVLGNTDRHSIKKNLIGALLFDSGETAEATRLKRTARRRYTRRKNRICYLQEIFSNEMAKVDDSFFHRLEESFL
VEEDKKHERHPIFGNIVDEVAYHEKYPTIYHLRKKLVDSTDKADLRLIYLALAHMIKFRGHFLIEGDLNPDNSDVDKLFIQ
LVQTYNQLFEENPINASGVDAKAILSARLSKSRRLENLIAQLPGEKKNGLFGNLIALSLGLTPNFKSNFDLAEDAKLQLSK
DTYDDDLDNLLAQIGDQYADLFLAAKNLSDAILLSDILRVNTEITKAPLSASMIKRYDEHHQDLTLLKALVRQQLPEKYKE
IFFDQSKNGYAGYIDGGASQEEFYKFIKPILEKMDGTEELLVKLNREDLLRKQRTFDNGSIPHQIHLGELHAILRRQEDFY
PFLKDNREKIEKILTFRIPYYVGPLARGNSRFAWMTRKSEETITPWNFEEVVDKGASAQSFIERMTNFDKNLPNEKVLPKH
SLLYEYFTVYNELTKVKYVTEGMRKPAFLSGEQKKAIVDLLFKTNRKVTVKQLKEDYFKKIECFDSVEISGVEDRFNASLG
TYHDLLKIIKDKDFLDNEENEDILEDIVLTLTLFEDREMIEERLKTYAHLFDDKVMKQLKRRRYTGWGRLSRKLINGIRDK
QSGKTILDFLKSDGFANRNFMQLIHDDSLTFKEDIQKAQVSGQGDSLHEHIANLAGSPAIKKGILQTVKVVDELVKVMGRH
KPENIVIEMARENQTTQKGQKNSRERMKRIEEGIKELGSQILKEHPVENTQLQNEKLYLYYLQNGRDMYVDQELDINRLSD
YDVDHIVPQSFLKDDSIDNKVLTRSDKNRGKSDNVPSEEVVKKMKNYWRQLLNAKLITQRKFDNLTKAERGGLSELDKAGF
IKRQLVETRQITKHVAQILDSRMNTKYDENDKLIREVKVITLKSKLVSDFRKDFQFYKVREINNYHHAHDAYLNAVVGTAL
IKKYPKLESEFVYGDYKVYDVRKMIAKSEQEIGKATAKYFFYSNIMNFFKTEITLANGEIRKRPLIETNGETGEIVWDKGR
DFATVRKVLSMPQVNIVKKTEVQTGGFSKESILPKRNSDKLIARKKDWDPKKYGGFDSPTVAYSVLVVAKVEKGKSKKLKS
VKELLGITIMERSSFEKNPIDFLEAKGYKEVKKDLIIKLPKYSLFELENGRKRMLASAGELQKGNELALPSKYVNFLYLAS
HYEKLKGSPEDNEQKQLFVEQHKHYLDEIIEQISEFSKRVILADANLDKVLSAYNKHRDKPIREQAENIIHLFTLTNLGAP
AAFKYFDTTIDRKRYTSTKEVLDATLIHQSITGLYETRIDLSQLGGDGTGGPKKKRKVYPYDVPDYAGYPYDVPDYAGSYP
YDVPDYAGSAAPAAKKKKLDFESGEFGAQPARSMAEAGITGTWYNQSGSTFTVTAGADGNLTGQYENRAQGTGCQNSPYTL
TGRYNGTKLEWRVEWNNSTENCHSRTEWRGQYQGGAEARINTQWNLTYEGGSGPATEQGQDTFTKVKPSAASGSPGGSTSS
RGSAAPAAKRVKLDGGSGGGSGGGSGSGPAAKRVKLD*

enAsCas12a-mSA: cMYC-NLS-Cas12a-HA-mSA

MASPAAKRVKLDGGSGGGSGGGSGPAAKRVKLDGGSGGGSGGGSGPLETQFEGFTNLYQVSKTLRFELIPQGKTLKHIQEQ
GFIEEDKARNDHYKELKPIIDRIYKTYADQCLQLVQLDWENLSAAIDSYRKEKTEETRNALIEEQATYRNAIHDYFIGRTD
NLTDAINKRHAEIYKGLFKAELFNGKVLKQLGTVTTTEHENALLRSFDKFTTYFSGFYRNRKNVFSAEDISTAIPHRIVQD
NFPKFKENCHIFTRLITAVPSLREHFENVKKAIGIFVSTSIEEVFSFPFYNQLLTQTQIDLYNQLLGGISREAGTEKIKGL
NEVLNLAIQKNDETAHIIASLPHRFIPLFKQILSDRNTLSFILEEFKSDEEVIQSFCKYKTLLRNENVLETAEALFNELNS
IDLTHIFISHKKLETISSALCDHWDTLRNALYERRISELTGKITKSAKEKVQRSLKHEDINLQEIISAAGKELSEAFKQKT
SEILSHAHAALDQPLPTTLKKQEEKEILKSQLDSLLGLYHLLDWFAVDESNEVDPEFSARLTGIKLEMEPSLSFYNKARNY
ATKKPYSVEKFKLNFQMPTLARGWDVNREKNNGAILFVKNGLYYLGIMPKQKGRYKALSFEPTEKTSEGFDKMYYDYFPDA
AKMIPKCSTQLKAVTAHFQTHTTPILLSNNFIEPLEITKEIYDLNNPEKEPKKFQTAYAKKTGDQKGYREALCKWIDFTRD
FLSKYTKTTSIDLSSLRPSSQYKDLGEYYAELNPLLYHISFQRIAEKEIMDAVETGKLYLFQIYNKDFAKGHHGKPNLHTL
YWTGLFSPENLAKTSIKLNGQAELFYRPKSRMKRMAHRLGEKMLNKKLKDQKTPIPDTLYQELYDYVNHRLSHDLSDEARA
LLPNVITKEVSHEIIKDRRFTSDKFFFHVPITLNYQAANSPSKFNQRVNAYLKEHPETPIIGIDRGERNLIYITVIDSTGK
ILEQRSLNTIQQFDYQKKLDNREKERVAARQAWSVVGTIKDLKQGYLSQVIHEIVDLMIHYQAVVVLENLNFGFKSKRTGI
AEKAVYQQFEKMLIDKLNCLVLKDYPAEKVGGVLNPYQLTDQFTSFAKMGTQSGFLFYVPAPYTSKIDPLTGFVDPFVWKT
IKNHESRKHFLEGFDFLHYDVKTGDFILHFKMNRNLSFQRGLPGFMPAWDIVFEKNETQFDAKGTPFIAGKRIVPVIENHR
FTGRYRDLYPANELIALLEEKGIVFRDGSNILPKLLENDDSHAIDTMVALIRSVLQMRNSNAATGEDYINSPVRDLNGVCF
DSRFQNPEWPMDADANGAYHIALKGQLLLNHLKESKDLKLQNGISNQDWLAYIQELRNGTGGPKKKRKVYPYDVPDYAGYP
YDVPDYAGSYPYDVPDYAGSAAPAAKKKKLDFESGEFGAQPARSMAEAGITGTWYNQSGSTFTVTAGADGNLTGQYENRAQ
GTGCQNSPYTLTGRYNGTKLEWRVEWNNSTENCHSRTEWRGQYQGGAEARINTQWNLTYEGGSGPATEQGQDTFTKVKPSA
ASGSPGGSTSSRGSAAPAAKRVKLDGGSGGGSGGGSGSGPAAKRVKLD*

SauCas9-mSA: cMYC-NLS-SauCas9-HA-mSA

MASPAAKRVKLDGGSGGGSGGGSGPAAKRVKLDGGSGGGSGGGSGPLEIHGVPAAKRNYILGLDIGITSVGYGIIDYETRD
VIDAGVRLFKEANVENNEGRRSKRGARRLKRRRRHRIQRVKKLLFDYNLLTDHSELSGINPYEARVKGLSQKLSEEEFSAA
LLHLAKRRGVHNVNEVEEDTGNELSTKEQISRNSKALEEKYVAELQLERLKKDGEVRGSINRFKTSDYVKEAKQLLKVQKA
YHQLDQSFIDTYIDLLETRRTYYEGPGEGSPFGWKDIKEWYEMLMGHCTYFPEELRSVKYAYNADLYNALNDLNNLVITRD
ENEKLEYYEKFQIIENVFKQKKKPTLKQIAKEILVNEEDIKGYRVTSTGKPEFTNLKVYHDIKDITARKEIIENAELLDQI
AKILTIYQSSEDIQEELTNLNSELTQEEIEQISNLKGYTGTHNLSLKAINLILDELWHTNDNQIAIFNRLKLVPKKVDLSQ
QKEIPTTLVDDFILSPVVKRSFIQSIKVINAIIKKYGLPNDIIIELAREKNSKDAQKMINEMQKRNRQTNERIEEIIRTTG
KENAKYLIEKIKLHDMQEGKCLYSLEAIPLEDLLNNPFNYEVDHIIPRSVSFDNSFNNKVLVKQEENSKKGNRTPFQYLSS
SDSKISYETFKKHILNLAKGKGRISKTKKEYLLEERDINRFSVQKDFINRNLVDTRYATRGLMNLLRSYFRVNNLDVKVKS
INGGFTSFLRRKWKFKKERNKGYKHHAEDALIIANADFIFKEWKKLDKAKKVMENQMFEEKQAESMPEIETEQEYKEIFIT

PHQIKHIKDFKDYKYSHRVDKKPNRELINDTLYSTRKDDKGNTLIVNNLNGLYDKDNDKLKKLINKSPEKLLMYHHDPQTY
QKLKLIMEQYGDEKNPLYKYYEETGNYLTKYSKKDNGPVIKKIKYYGNKLNAHLDITDDYPNSRNKVVKLSLKPYRFDVYL
DNGVYKFVTVKNLDVIKKENYYEVNSKCYEEAKKLKKISNQAEFIASFYNNDLIKINGELYRVIGVNNDLLNRIEVNMIDI
TYREYLENMNDKRPPRIIKTIASKTQSIKKYSTDILGNLYEVKSKKHPQIIKKGGTGGPKKKRKVYPYDVPDYAGYPYDVP
DYAGSYPYDVPDYAGSAAPAAKKKKLDFESGEFGAQPARSMAEAGITGTWYNQSGSTFTVTAGADGNLTGQYENRAQGTGC
QNSPYTLTGRYNGTKLEWRVEWNNSTENCHSRTEWRGQYQGGAEARINTQWNLTYEGGSGPATEQGQDTFTKVKPSAASGS
PGGSTSSRGSAAPAAKRVKLDGGSGGGSGGGSGSGPAAKRVKLD*

Supplementary Note 1. FACS gating examples for GFP-positive cells.

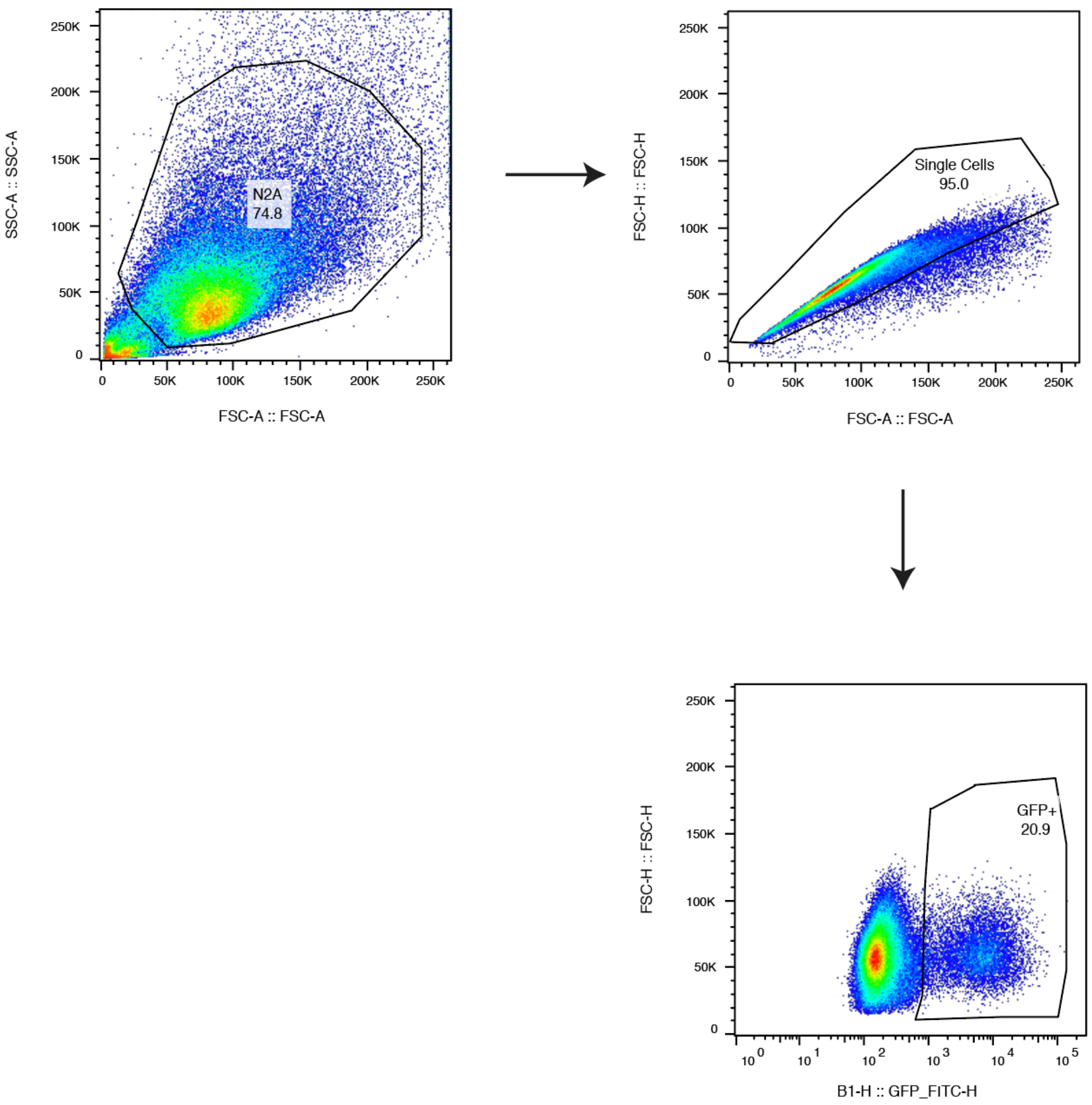

FACS gating examples for tdTomato-positive cells from mouse lung.

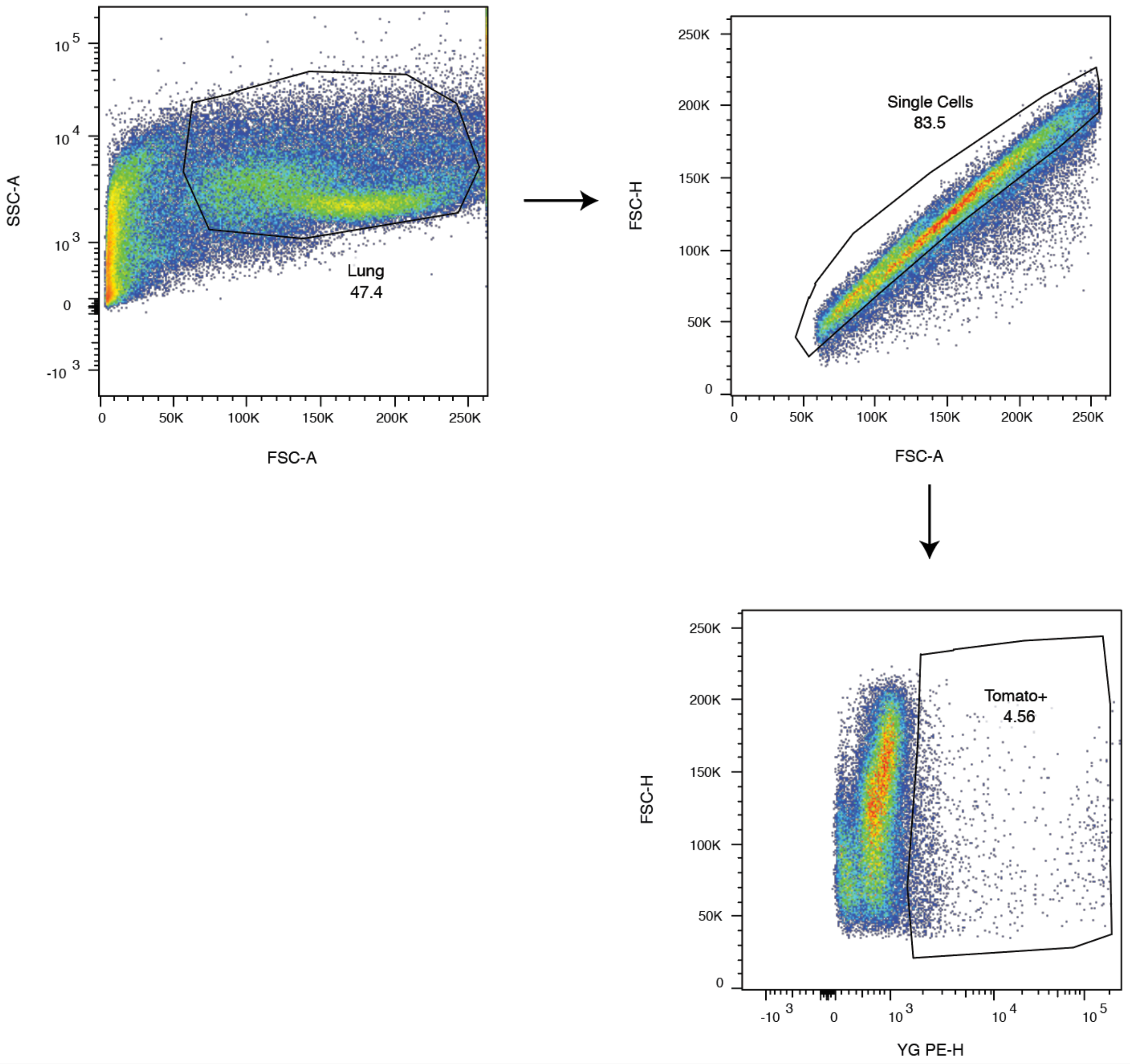

Supplement: Supplementary file 1 — Supplementary information [file 41467_2022_28135_MOESM1_ESM.pdf]
