# Supplementary material for: Genome-wide detection of CRISPR editing in vivo using GUIDE-tag
Source: Nat Commun. 2022 Jan 21;13:437. doi: 10.1038/s41467-022-28135-9 (PMC8782884; doi:10.1038/s41467-022-28135-9)
Supplement: Supplementary file 2 — Reporting Summary [file 41467_2022_28135_MOESM2_ESM.pdf]

Corresponding author(s): Scot A. Wolfe, Wen Xue

Last updated by author(s): Jan 4, 2022

Reporting Summary

Nature Research wishes to improve the reproducibility of the work that we publish. This form provides structure for consistency and transparency in reporting. For further information on Nature Research policies, see our Editorial Policies and the Editorial Policy Checklist.

Statistics

For all statistical analyses, confirm that the following items are present in the figure legend, table legend, main text, or Methods section.

n/a	Confirmed	
☐	☒	The exact sample size (*n*) for each experimental group/condition, given as a discrete number and unit of measurement
☐	☒	A statement on whether measurements were taken from distinct samples or whether the same sample was measured repeatedly
☐	☒	The statistical test(s) used AND whether they are one- or two-sided<br>*Only common tests should be described solely by name; describe more complex techniques in the Methods section.*
☒	☐	A description of all covariates tested
☒	☐	A description of any assumptions or corrections, such as tests of normality and adjustment for multiple comparisons
☐	☒	A full description of the statistical parameters including central tendency (e.g. means) or other basic estimates (e.g. regression coefficient) AND variation (e.g. standard deviation) or associated estimates of uncertainty (e.g. confidence intervals)
☐	☒	For null hypothesis testing, the test statistic (e.g. *F*, *t*, *r*) with confidence intervals, effect sizes, degrees of freedom and *P* value noted<br>*Give P values as exact values whenever suitable.*
☒	☐	For Bayesian analysis, information on the choice of priors and Markov chain Monte Carlo settings
☒	☐	For hierarchical and complex designs, identification of the appropriate level for tests and full reporting of outcomes
☐	☒	Estimates of effect sizes (e.g. Cohen's *d*, Pearson's *r*), indicating how they were calculated

Our web collection on statistics for biologists contains articles on many of the points above.

Software and code

Policy information about availability of computer code

Data collection	MiniSeq SY-420-1001, FlowJo 10.8.0, Prism 9 for macOS Version 9.1.1, RStudio Version 1.2.5033
Data analysis	See Methods

For manuscripts utilizing custom algorithms or software that are central to the research but not yet described in published literature, software must be made available to editors and reviewers. We strongly encourage code deposition in a community repository (e.g. GitHub). See the Nature Research guidelines for submitting code & software for further information.

Data

Policy information about availability of data

All manuscripts must include a data availability statement. This statement should provide the following information, where applicable:
- Accession codes, unique identifiers, or web links for publicly available datasets
- A list of figures that have associated raw data
- A description of any restrictions on data availability

The next-generation sequencing data have been deposited in the NCBI Sequence Read Archive database under the bioProject accession code PRJNA726835. All the databases/datasets used in the study along with appropriately accessible links/accession-codes in the manuscript under the “Data availability” section.

Field-specific reporting

Life sciences study design

All studies must disclose on these points even when the disclosure is negative.

Sample size	Sample size was not pre-determined by statistical methods, but rather, based on preliminary data.
Data exclusions	No data was excluded.
Replication	All attempts (n=3) at replication were successful, and standard deviations were in the expected ranges.
Randomization	Group allocation for animal study was performed randomly. The same cell passages were used for the biological replicates, the results were confirmed by different cells passages.
Blinding	Not blinded. Animal groups were labeled with card.

Reporting for specific materials, systems and methods

We require information from authors about some types of materials, experimental systems and methods used in many studies. Here, indicate whether each material, system or method listed is relevant to your study. If you are not sure if a list item applies to your research, read the appropriate section before selecting a response.

Materials & experimental systems

n/a	Involved in the study	
☐	☒	Antibodies
☐	☒	Eukaryotic cell lines
☒	☐	Palaeontology and archaeology
☐	☒	Animals and other organisms
☒	☐	Human research participants
☒	☐	Clinical data
☒	☐	Dual use research of concern

Methods

n/a	Involved in the study	
☒	☐	ChIP-seq
☐	☒	Flow cytometry
☒	☐	MRI-based neuroimaging

Antibodies

Antibodies used	Anti-streptavidin antibody (1:100, Vector, Cat. # BA-0500-.5), Fah antibody (1:100, Abcam, Cat. #83770), anti-GFP (1:200, CST, Cat. #2956)
Validation	The specificity of the Fah antibody has previously been confirmed (Yin et al, NBT, 2016). The specificity of the Streptavidin and GFP antibody has been confirmed in the manuscript.

Eukaryotic cell lines

Policy information about cell lines

Cell line source(s)	HEK293T (ATCC), Neuro 2A (ATCC), HepA1-6 (ATCC)
Authentication	Cell lines were authenticated by STR by University of Arizona Genetic core and using PCR assays with species-specific primers
Mycoplasma contamination	Cell lines were tested negative for mycoplasma
Commonly misidentified lines (See ICLAC register)	No

Animals and other organisms

Policy information about studies involving animals; ARRIVE guidelines recommended for reporting animal research

Laboratory animals	6-8 weeks female transgenic Fah(deltaExon5) mice, C57BL/6J mice and FVB/NJ mice were used.
Wild animals	*Provide details on animals observed in or captured in the field; report species, sex and age where possible. Describe how animals were caught and transported and what happened to captive animals after the study (if killed, explain why and describe method; if released, say where and when) OR state that the study did not involve wild animals.*
Field-collected samples	No field collected samples were used in the study.
Ethics oversight	All animal study protocols were approved by the UMass IACUC.

Note that full information on the approval of the study protocol must also be provided in the manuscript.

Flow Cytometry

Plots

Confirm that:

- [x] The axis labels state the marker and fluorochrome used (e.g. CD4-FITC).
- [x] The axis scales are clearly visible. Include numbers along axes only for bottom left plot of group (a 'group' is an analysis of identical markers).
- [x] All plots are contour plots with outliers or pseudocolor plots.
- [x] A numerical value for number of cells or percentage (with statistics) is provided.

Methodology

Sample preparation	Cell lines were detached and performed Flow cytometry analysis immediately.
Instrument	LSRII and MACSQuant
Software	FlowJo
Cell population abundance	No purfication
Gating strategy	Cell populations were analyzed for GFP or tdTomato expression

- [x] Tick this box to confirm that a figure exemplifying the gating strategy is provided in the Supplementary Information.
