# Supplementary material for: Genome-wide detection of CRISPR editing in vivo using GUIDE-tag
Source: Nat Commun. 2022 Jan 21;13:437. doi: 10.1038/s41467-022-28135-9 (PMC8782884; doi:10.1038/s41467-022-28135-9)
Supplement: Supplementary file 3 — Description of Additional Supplementary Files [file 41467_2022_28135_MOESM3_ESM.pdf]

Description of Additional Supplementary Files

Title: Supplementary Data 1.
Description: *In vivo* off target analysis of sgFah by GUIDE-tag. Sheet 1, UMI number of GUIDE-tag and indel frequency in Fah mice determined by deep sequencing. **Sheet 2**, Statistical analysis of targeted amplicon deep sequencing. p-values were calculated by two-tailed Student's t-test. To adjust for multiple comparisons p-values were adjusted using the Benjamini-Hochberg (BH) method.

Title: Supplementary Data 2.
Description: Potential off-target sites predicted with the Fah sgRNA by CRISPRseek.

Title: Supplementary Data 3.
Description: *In vivo* on target analysis at Actin site by UdiTaS. UMI number of UdiTaS in FVB mice determined by deep sequencing.

Title: Supplementary Data 4.
Description: *In vivo* off target analysis of sgActin by GUIDE-tag. Sheet 1, Indel frequency in FVB mice determined by deep sequencing. **Sheet 2**, Statistical analysis of targeted amplicon deep sequencing. p-values were calculated by two-tailed Student's t-test. To adjust for multiple comparisons p-values were adjusted using the Benjamini-Hochberg (BH) method.

Title: Supplementary Data 5.
Description: *In vivo* off target analysis of sgPcsk9 by GUIDE-tag in mouse liver. Sheet 1, RAW UMI number of GUIDE-tag in B6 mice determined by deep sequencing. **Sheet 2,** UMI number (Filter 1) of GUIDE-tag in B6 mice determined by deep sequencing. **Sheet 3,** UMI number (Filter 2) of GUIDE-tag in B6 mice determined by deep sequencing. **Sheet 4,** Indel frequency in B6 mice determined by deep sequencing. **Sheet 5**, Statistical analysis of targeted amplicon deep sequencing. p-values were calculated by two-tailed Student's t-test. To adjust for multiple comparisons p-values were adjusted using the Benjamini-Hochberg (BH) method

Title: Supplementary Data 6.
Description: *In vivo* off target analysis of sgPCSK9 by GUIDE-tag in mouse liver with GUIDE donor or iGUIDE donor. Sheet 1, RAW UMI number of GUIDE-tag in B6 mice determined by deep sequencing. **Sheet 2,** UMI number (Filter 1) of GUIDE-tag in B6 mice determined by deep sequencing. **Sheet 3,** Indel frequency in B6 mice determined by deep sequencing.

Title: Supplementary Data 7.
Description: *In vivo* translocation and large deletion analysis by UdiTaS. Sheet 1, UMI number of Translocations in Fah, FVB and B6 mice determined by deep sequencing. **Sheet 2**, Statistical analysis of translocations. p-values were calculated by two-tailed Student's t-test. To adjust for multiple comparisons p-values were adjusted using the Benjamini-Hochberg (BH) method.

Title: Supplementary Data 8.
Description: *In vivo* off target analysis of sgPCSK9 by GUIDE-tag in mouse lung. Sheet 1, UMI number of GUIDE-tag in Ai9 mice determined by deep sequencing. **Sheet 2**, Indel frequency in Ai9 mice determined by deep sequencing.
